# Can Simple Psychological Interventions Increase Preventive Health Investment?

**DOI:** 10.1093/jeea/jvab052

**Published:** 2021-11-30

**Authors:** Anett John, Kate Orkin

**Affiliations:** Department of Economics, University of Birmingham, UK; Blavatnik School of Government and Centre for Study of African Economies, University of Oxford, UK

## Abstract

Behavioral constraints may explain part of the low demand for preventive health products. We test the effects of two light-touch psychological interventions on water chlorination and related health and economic outcomes using a randomized controlled trial among 3,750 women in rural Kenya. One intervention encourages participants to visualize alternative realizations of the future, and the other builds participants’ ability to make concrete plans. After 12 weeks, visualization increases objectively measured chlorination, reduces diarrhea episodes among children, and increases savings. Effects on chlorination and savings persist after almost 3 years. Effects of the planning intervention are weaker and largely insignificant. Analysis of mechanisms suggests both interventions increase self-efficacy—beliefs about one’s ability to achieve desired outcomes. Visualization also increases participants’ skill in forecasting their future utility. The interventions do not differentially affect beliefs and knowledge about chlorination. Results suggest simple psychological interventions can increase future-oriented behaviors, including use of preventive health technologies.

**Teaching Slides**

A set of Teaching Slides to accompany this article are available online as Supplementary Data.

## 1. Introduction

Individuals often fail to invest in preventive healthcare, even when such investments cost little and individuals are aware of their benefits.^1^ A prominent example is chlorination of drinking water, which is highly effective in reducing prevalence of diarrhea, particularly among young children (Arnold and Colford 2007). Diarrhea is the second leading cause of death worldwide among children aged 1–5, contributing to nearly half a million deaths in 2015 (Wang et al. 2016). It is a leading cause of morbidity and stunts healthy growth in children through enteric dysfunction (Richard et al. 2013). In many settings, chlorine for water is readily and cheaply available, but infrequently used by individuals without access to clean water. In our study areas in Kenya, only 3% of households used chlorine before any intervention (Null et al. 2018), although a month’s supply costs only 25 Kenyan shillings (KES, USD 0.25). Interventions that provide chlorine for free, often in combination with information or marketing campaigns, increase usage to between 23 and 60%, but take-up remains far below complete (Dupas et al. 2016, 2020; Luoto et al. 2014; Null et al. 2018).

A growing body of evidence suggests that behavioral or psychological constraints may explain some of the low demand for preventive health products. For example, demand for commitment products suggests a role for present bias in health decisions (Bai et al. 2021; DellaVigna and Malmendier 2006; Giné, Karlan, and Zinman 2010; Schilbach 2019). The success of planning interventions suggests that planning skills and limited attention may be a factor (Milkman et al. 2011; Stadler, Oettingen, and Gollwitzer 2009). Most recently, economists have explored the role of beliefs about oneself—from self-image to agency and sense of control over one’s life—in determining health behavior (Ghosal et al. 2020).

In this paper, we present evidence from a field experiment in rural Kenya that studies the role of behavioral constraints in limiting the use of chlorine to treat drinking water by targeting these constraints directly with simple psychological and informational interventions. We allocate 3,750 young women to four groups. The first group received a two-session group intervention where participants visualized alternative realizations of the future, depending on their behavior in the present (“Visualization” or “V”). The intervention aimed to increase patient behavior by making future outcomes more vivid and tangible in participants’ minds, thus increasing their perceived value relative to the more immediate costs required to attain them. The second group received a two-session intervention that aimed to improve planning skills, helping participants to undertake activities that they were struggling to do regularly by making specific plans and establishing routines (“Planning” or “P”). Both interventions required participants to think about how their current behavior affects their future outcomes. A third, active control group (“Active Control” or “AC”) also gathered as a group for workshops of the same format, but to discuss topics likely to be psychologically inactive (birds in Kenya). In addition, all three groups received a short information module about the benefits of chlorination (“Information” or “INF” intervention), to hold beliefs about chlorination constant across groups. Finally, we compare these treatments to a fourth, pure control group (“Pure Control” or “PC”), who were simply surveyed at endline. Thus, our groups are V+INF, P+INF, AC+INF, and PC. The comparison between the two psychological treatment groups and the AC+INF group, on which we focus, tests the effect of interventions targeting the ability to visualize the future or to make and execute plans, respectively, over and above those targeting lack of information. This comparison is of primary academic interest, as it best isolates the mechanisms at work. The comparison to the pure control group gives the total effect of providing interventions such as ours in similar settings. We measure health, economic, and psychological effects of our interventions using in-person surveys and choice tasks after 10 weeks, and also conduct chemical tests of chlorine levels in water in unannounced household visits after 12 weeks. We follow up after 30–36 months using phone surveys, given pandemic-related safety concerns in 2020.

First, we report economically large and statistically significant effects of Visualization on our main outcome of interest, objectively measured chlorination of household drinking water. Specifically, we find a significant increase of 22% (5 percentage points) in the share of households whose drinking water contains chlorine, relative to the active control group, 12 weeks after the interventions. Self-reports confirm this finding. We show suggestive evidence that this effect persists over time: After almost 3 years, when asked an unprompted question about how they treat their water, participants in the Visualization group are 5 percentage points more likely to state that they chlorinate it. The number of diarrhea episodes per child under 15 decreases by 46% (0.12 episodes) relative to the active control after 10 weeks. This effect does not persist over time, although we show this may be due to differences in season between rounds. In contrast, the Planning intervention has positive but largely insignificant effects on health outcomes: Chlorination does not increase in either round. Diarrhea episodes decrease somewhat (potentially driven by an increase in boiling water), but neither effect persists over time.

The Visualization intervention is highly cost-effective, even under conservative assumptions. Delivery of the interventions costs approximately USD 4 per household. Considering only benefits to children under 5, the cost per Disability-Adjusted Life Year (DALY) saved is USD 248.^2^ The WHO classifies an intervention as “cost-effective” for a cost per DALY saved below USD 4,525, and “highly cost-effective” below USD 1,508.

Second, while our primary interest is in preventive health investments, our interventions are domain-general in nature, and may thus reduce barriers to forward-looking behavior in general. Indeed, we observe that the Visualization intervention led to significant increases in all four pre-specified savings indicators (amount saved per week, likelihood of saving at all, whether participants joined new rotating savings and credit associations (ROSCAs), and likelihood of saving for productive investments). These effects persist after almost 3 years, allowing the Visualization group to accumulate 41% higher savings balances. The long-term effects are observed during the 2020 global pandemic, which decreased real income in rural Kenya by 7.9% (Nechifor et al. 2020), suggesting that participants who learned visualization techniques in 2017 benefited from an increased financial buffer in 2020.

Third, we test if our psychological interventions have larger effects in villages where infrastructure lowers the monetary and time costs of chlorination. We conducted our intervention in the study sites of a previous trial, the “WASH Benefits” study (Null et al. 2018). In this study, villages were randomly assigned to receive chlorine dispensers placed at the water source, and dispensers have been maintained since the trial. Our treatment effects on chlorination and diarrhea are very similar in both types of villages. Our results suggest that relieving psychological constraints is effective, whether or not cost and access barriers remain.

Fourth, we use lab-in-the-field methods and psychological questionnaires to study effects on pre-specified mechanisms, which were targeted by the interventions. We hypothesized that Visualization may affect time preferences and self-efficacy, while Planning may affect planning skills and self-efficacy.^3^ We find that conventional laboratory measures of time preferences—both an incentivized real-effort task and multiple price lists (MPLs) over money—do not respond to our interventions. The interventions also have no measurable impact on self-reported planning behavior, nor on underlying cognitive functions linked to planning. In contrast, we show large, significant effects of both the Visualization and Planning treatments on self-efficacy. After 10 weeks, self-efficacy increases by 0.15 and 0.11 standard deviations, respectively. In the long-term follow-up, participants in the Visualization group maintain significantly higher levels of self-efficacy than those in the Planning group, consistent with the differential long-term effects on chlorination.

While conventional measures of time preferences did not respond to our Visualization intervention, we find suggestive evidence of improved *utility forecasting*, a conceptualization of intertemporal choice recently proposed by Gabaix and Laibson (2017). In this framework, decision-makers simulate future utility by combining priors with noisy, unbiased signals. Imperfect foresight of future utility results in choices that are biased towards the present, with choice patterns resembling those predicted by hyperbolic discounting models. The framework implies that interventions that reduce forecasting noise will lead to more patient behavior without changing the underlying preference parameters. Consistent with this prediction, we find that participants in the Visualization group report significantly more clear and vivid mental forecasts of the future after almost 3 years.^4^ There is no such effect in the Planning group, which may explain the differential effects on both chlorination and savings. Summarizing our results on mechanisms, our results suggest that deeper underlying preferences and cognitive functions may not respond strongly to light-touch interventions. In contrast, beliefs about the self (such as self-efficacy), as well as the ability to imagine the future, seem to be both malleable in the short and long term and potentially powerful drivers of human behavior.

Finally, we test a range of alternative plausible psychological channels. First, we show that differences in behavior are not explained merely by differences in beliefs about the efficacy of chlorine or increased knowledge about chlorination. All three “active” treatment groups received the information treatment and showed similar improvements in their belief that chlorination can prevent diarrhea and in their knowledge about using chlorine. Second, our interventions do not affect lab measures of risk preferences. Third, using a “salience” task, we find that participants’ attention is more focused on chlorination in both psychological treatment groups than in the information treatment alone, even 10 weeks after the interventions. In the Visualization group, this effect appears to grow *larger* after almost 3 years. We cannot disentangle whether increased salience causes or is caused by increased use of chlorine. However, given the long-term nature of the salience effects and the fact that the salience of savings does not increase but savings do increase, the evidence is more consistent with reverse causality than with salience as a driver of treatment effects.

Although we cannot completely rule out that participants were influenced by social desirability bias in answering questions, it is unlikely that such effects account for our findings. Most importantly, we observe increases in objectively measured chlorine content of stored household drinking water during unannounced household visits after 12 weeks. In the long-run survey, where we only measure self-reported chlorination, we also conduct explicit experimenter demand treatments (following de Quidt, Haushofer, and Roth 2018, and orthogonal to our psychological treatments). We find that explicitly telling the respondents the experimenter’s hypothesis has no effect on self-reported chlorination, suggesting social desirability bias is limited.

Our paper contributes to existing work in behavioral and development economics on psychological factors that affect investment decisions. First, and beyond the health domain, to the best of our knowledge, our paper is the first to show the effects of a visualization-style intervention on real-world behavior of adults, in multiple domains. We understand visualization as the simulation of *expected* future utility and thus as distinct from goal-setting (Gollwitzer and Sheeran 2006). Psychologists have long been interested in interventions that fall into this category. Typically, interventions are administered *while* the outcome behavior is observed in a laboratory setting: In “Episodic Future Thinking (EFT)”, participants’ mental focus is shifted to unrelated future events, such as attending a birthday party next month (Daniel, Stanton, and Epstein 2013). Vividness interventions aim to make the future self more vivid, typically by showing age-progressed photos or avatars of the decisionmaker (Hershfield, John, and Reiff 2018; Hershfield et al. 2011). Sample sizes are usually around or below 50. Alan and Ertac (2018) take visualization techniques to the field, and show that an eight-session intervention in Turkish primary schools leads children to make more patient decisions in incentivized choice tasks almost 3 years later. We innovate on this literature by showing that even light-touch visualization interventions can affect real-world behavior of adults, 12 weeks and 3 years after the intervention. We also elicit comprehensive measures of the mechanisms through which the intervention may work. Our work is also distinct from studies testing light-touch interventions to increase aspirations (Baranov, Haushofer, and Jang 2020; Bernard et al. 2019; Orkin et al. 2020). A key difference is that such interventions assume decision-makers with reference-dependent preferences and aim to enhance effort and investment by raising people’s reference points (Genicot and Ray 2020). Instead, we present suggestive evidence that visualization increases patient behavior both by strengthening the belief that current behaviors affect future outcomes (self-efficacy),^5^ and by improving participants’ mental forecasts of the future (Gabaix and Laibson 2017).^6^

Second, we compare our Visualization intervention against another intervention aimed to increase future-oriented behavior: Planning. This comparison of two interventions that target different psychological mechanisms is novel and allows us to explore which psychological targets are the most malleable to intervention and which most affect behavior. The Planning intervention was adapted from a psychotherapy approach called Behavioral Activation (BA), which teaches people simple, structured planning skills to help them undertake activities that they have identified as important but are struggling to do regularly (Ekers et al. 2014; Lejuez et al. 2011). To the best of our knowledge, the effects of BA on economic behavior or preventive health choices have not been shown.^7^ Economists have examined effects of a related but distinct therapy, Cognitive Behavioral Therapy (CBT) (Baranov et al. 2020; Blattman, Jamison, and Sheridan 2017). Relative to this work, we work with a general population, rather than those suffering from depression or who are engaged in criminal behavior. We also study a different therapy: CBT interventions are typically bundled, containing multiple modules teaching different skills and cognitions. They are usually longer (typically seven to eight sessions) and require skilled personnel, while BA is shorter and facilitators require little training (Patel et al. 2017; Richards et al. 2016). Our Planning intervention is also distinct from “implementation intentions” interventions (Duckworth et al. 2013; Gollwitzer and Sheeran 2006), as well as from planning prompts (Milkman et al. 2011; Stadler, Oettingen, and Gollwitzer 2009). These identify a specific domain of behavior where participants are encouraged to make changes, while in the Planning intervention, participants identified areas where they aimed to change behavior themselves.

Third, we contribute to the question of how to increase preventive health investment in low-income countries. Within this literature, to the best of our knowledge, we are the first to study a domain-general psychological intervention, which is light-touch and easy to scale. A large literature has studied the impact of information (Jalan and Somanathan 2008; Madajewicz et al. 2007), price (Ashraf, Berry, and Shapiro 2010; Kremer et al. 2011b), combinations of information and subsidies (Ashraf, Jack, and Kamenica 2010b; Kremer et al. 2011a; Luoto et al. 2011; Null et al. 2018), and non-monetary costs (Dupas et al. 2016, 2020) on the demand for preventive healthcare.^8^ Following the hypothesis that the remaining barriers to adoption may be behavioral, some studies use “nudges” to complement information or free provision. These have included verbal commitments (Dupas 2009; Kremer and Miguel 2007) or marketing messages (Dupas 2009; Kremer et al. 2011a). They have also shown limited effectiveness, except when they are highly personalized (e.g. Luoto et al. 2014 prints posters using photos of the respondent) and thus potentially difficult to scale. Nudges are also targeted to specific, momentary behaviors, and do not attempt to change fundamental preferences or constraints. We contribute to this literature by showing that domain-general psychological interventions can be a low-cost and scalable tool to increase preventive health investments. In combination with subsidy or information campaigns, they may be used to deliver clean water to the 2 billion people who currently use drinking water contaminated with feces.^9^

The paper proceeds as follows. Section 2 describes the study design. Section 3 describes the interventions. Section 4 describes the outcome variables. Section 5 describes the estimation approach. Section 6 reports results. Section 7 discusses potential mechanisms. Section 8 concludes.

## 2. Experimental Design

### 2.1. Sampling and Randomization

Our study areas are Bungoma and Kakamega counties in rural Western Kenya. These counties were included in the WASH Benefits study (henceforth, WASH), a cluster-randomized controlled trial conducted from 2012 to 2014 in 1,226 villages (Null et al. 2018). Villages were randomized to eight arms testing household-level water, sanitation, handwashing, and nutrition interventions, either in isolation or in combination.^10^

We sampled 205 villages from the WASH study and recruited 3,750 women aged 18–35 between October 2017 and January 2018 in these villages. Women are primarily responsible for collecting water and, thus, for chlorination. We recruited women aged 18–35 as they are the most likely to have small children, who are the most vulnerable to water-borne illnesses. Enumerators visited all households in each village and conducted a census to determine household eligibility. Screening criteria included: (i) the woman was aged 18–35 inclusive, (ii) within this age range, the woman was the most senior woman in their household, and (iii) the woman's household did not participate in the WASH study. The target sample of 3,750 women represents all eligible women in the 205 villages. As shown in Table 1, the women in our sample are on average 27 years old and have an average 6 years of education. A total of 89% are married or cohabiting. We split our sample into three “active” treatment groups and one pure control group. We randomly assign 992 participants to the Visualization (V+INF) group, 991 to the Planning (P+INF) group, 992 to the Active Control (AC+INF) group, and 775 to the Pure Control group. We stratify the randomization on village of residence and a wealth index.^11^

**Table 1.** Experimental integrity.

	Comparison with active control (AC+INF)					Comparison with pure control (PC)				
	(1)	(2)	(3)	(4)	(5)	(6)	(7)	(8)	(9)	(10)
	Active control group mean (SD)	Visualization treatment effect	Planning treatment effect	Column 2 versus column 3 *p*-value	*N*	Pure control mean (SD)	V+INF treatment effect	P+INF treatment effect	AC+INF treatment effect	*N*
*Balance on recruitment census variables*										
Age	26.57	-0.02	0.00	0.93	2,337	26.62	-0.42	-0.36	-0.31	3,750
	(4.52)	(0.22)	(0.22)			(4.69)	(0.22)$^{*}$	(0.22)	(0.22)	
Married or cohabiting	0.89	-0.00	0.00	0.76	2,337	0.90	-0.02	-0.01	-0.02	3,750
	(0.31)	(0.02)	(0.02)			(0.30)	(0.02)	(0.01)	(0.02)	
Education level	5.85	0.01	0.08	0.19	2,337	5.93	-0.08	0.00	-0.05	3,750
	(1.22)	(0.06)	(0.06)			(1.08)	(0.05)	(0.05)	(0.05)	
High wealth index	0.55	-0.02	-0.03	0.67	2,337	0.52	0.00	-0.01	0.02	3,750
	(0.50)	(0.02)	(0.02)			(0.50)	(0.02)	(0.02)	(0.02)	
Village of residence	87.59	0.46	0.42	0.99	2,337	83.31	0.80	-0.24	-0.07	3,750
	(54.51)	(6.11)	(6.05)			(56.43)	(4.18)	(4.14)	(3.97)	
*Survey participation*										
Did not participate in endline (10 w)	0.08	0.02	0.03	0.39	2,337	0.24	-0.06	-0.04	-0.06	3,750
	(0.27)	(0.02)	(0.02)$^{*}$			(0.43)	(0.02)$^{***}$	(0.02)$^{*}$	(0.02)$^{***}$	
Did not participate in chlorine test (12 w)	0.12	0.01	0.03	0.33	2,337	0.26	-0.04	-0.02	-0.04	3,750
	(0.33)	(0.02)	(0.02)			(0.44)	(0.02)$^{*}$	(0.02)	(0.02)$^{**}$	
Did not participate in follow-up (33 m)	0.10	0.02	0.02	0.85	2,337	0.17	-0.01	-0.02	-0.01	3,750
	(0.31)	(0.02)	(0.02)			(0.37)	(0.02)	(0.02)	(0.02)	
*Delay variables*										
Days between endline and baseline	67.73	0.62	1.80	0.23	2,116	68.73	1.82	2.12	1.23	2,984
	(20.63)	(1.00)	(0.93)$^{*}$			(24.07)	(1.06)$^{*}$	(1.01)$^{**}$	(1.00)	
Days between chlorine test and baseline	79.19	0.73	2.62	0.16	2,007	81.58	0.75	1.57	-0.01	2,832
	(26.42)	(1.35)	(1.31)$^{**}$			(27.27)	(1.21)	(1.17)	(1.16)	
Chlorine test was conducted on first day in village	0.68	0.01	-0.00	0.69	2,009	0.67	-0.01	-0.01	-0.01	2,839
	(0.47)	(0.02)	(0.02)			(0.47)	(0.02)	(0.02)	(0.02)	
Days between follow-up and baseline	980.47	2.76	2.02	0.70	2,066	987.06	-1.16	-2.35	-4.36	2,828
	(33.29)	(1.98)	(1.86)			(36.04)	(1.91)	(1.82)	(1.84)$^{**}$	
*Compliance*										
Completed baseline and both interventions	0.74	0.01	–0.02	0.35	2,975	–	–	–	–	–
	(0.44)	(0.02)	(0.02)			–	–	–	–	–
Completed baseline and first intervention	0.78	0.01	0.01	0.85	2,975	–	–	–	–	–
	(0.41)	(0.02)	(0.02)			–	–	–	–	–

Notes: OLS estimates of balance across treatment groups. For each variable, columns (1) and (6) report the mean and standard deviation of the control group. Columns (2) and (3) and columns (7) and (8) report the coefficients of interest and standard errors in parentheses, respectively. Survey participation, delay, and compliance specifications control for a vector of observed characteristics; randomization balance specifications do not. All specifications cluster standard errors at the level of the intervention cohort. In the active control comparison, the sample is restricted to those who completed baseline and the first intervention session, since these are the samples used for the primary analyses. The exceptions are the “compliance” regressions, where baseline and intervention attendance are the outcomes of interest. Survey participation can only be interpreted as attrition in the active control comparison: In the pure control comparison, we cannot condition on baseline participation, and thus include all participants from the recruitment census. Balance variables were collected during the recruitment census in the villages. “High wealth index” denotes participants with an above-median value of assets from a limited list of common household assets.

*denotes significance at the 10%, ** at the 5%, and *** at the 1% levels.

We run our study in WASH sample villages because there is random variation between WASH treatment arms in levels of access to chlorine dispensers and hence in the monetary and time costs households face to chlorinate. We cross-cut this village-level variation with our household-level randomization to psychological treatments. We analyze the effects of psychological interventions on take-up of preventive health products when take-up is more or less costly.

We randomly selected 90 villages from the WASH arms that receive chlorine dispensers (henceforth, “dispenser villages”). First, we randomly selected 67 villages from the “Water Quality” treatment arm of the WASH study. In this treatment, chlorine dispensers were installed at an average of five community water points per village cluster, and community promoters encouraged their use. The NGO *Evidence Action* still maintains these dispensers, ensures they are filled with chlorine, and retains a local promoter in each community. In these villages, the cost of chlorinating is lower. Second, we sample 23 villages who received the “Water Quality” intervention in combination with other interventions, detailed in Online Appendix C. All other interventions (sanitation, handwashing, nutrition, or a combination thereof) took place at the household level and finished three to four years before our study. Our sampling excludes households that participated directly in the original WASH study. As a result, we simply classify all 90 villages as dispenser villages.

We also randomly selected 105 “non-dispenser” WASH villages. Of these villages, 67 were in the WASH “Passive Comparison” arm, which received no interventions. The other 48 villages were in WASH treatment arms which received no dispensers but did receive other household-level interventions. As above, former WASH beneficiary households are excluded from our study. In the 105 non-dispenser villages, the monetary and time costs of chlorinating are higher. The randomization has remained intact, so no dispensers have been provided by *Evidence Action* in non-dispenser villages.

### 2.2. Design and Timeline

**Figure 1.**
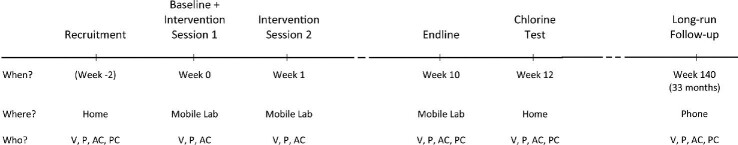
Timeline of the study.

Participants in the three active treatment groups participated in four sessions: a combined baseline and first intervention session, a second intervention session 1 week later, an endline 10 weeks after the first session, and a long-term follow-up after 30–36 months. Participants in the pure control group did not participate in the baseline or the interventions. Households in all four groups received an unannounced visit 12 weeks after the first session, where enumerators collected a drinking water sample. Figure 1 shows the study timeline.

We conducted the baseline survey, treatment interventions, and 10-week endline survey in “mobile labs” operated by the Busara Center. The baseline and endline surveys lasted about two hours each. Behavioral tasks and psychological questionnaires were self-administered using touch screen computers and color-coded response buttons in zTree (Fischbacher 2007). Enumerators read out instructions in Kiswahili to maximize comprehension, though all participants were literate. Economic and health questions were administered one-on-one by enumerators.

For the intervention, participants were split into the same cohorts of five for both sessions. No participant was invited for the second session without having already participated in the first session. Each intervention session lasted about two hours. Interventions were run by locally-trained female facilitators.

Between July and December 2020, an average of 33 months after the interventions, we conducted a long-term follow-up survey. Given public health restrictions in 2020, this survey was conducted by phone and lasted about 30 minutes. To minimize attrition, enumerators went back to the villages of all respondents whose phone numbers were no longer operational (about 22%) during an intensive tracking stage, to collect new phone numbers. To ensure consistency of surveying method and respondent safety, surveys were conducted by phone even in these cases.

Participants received participation fees of KES 250 (USD 2.50) for the baseline and each intervention session, KES 350 for the 10-week endline survey, and KES 100 for the long-term follow-up.^12^ Participants were reimbursed for their transport costs to the mobile laboratory (typically 30 minutes of travel time). They also received payments from the experimental tasks, as described in Section 4. Typical total study payments (excluding transport) were around KES 1,700 in the active treatment groups, and around KES 850 in the pure control group.^13^ All recruited participants were invited to attend the endline and follow-up surveys, regardless of whether they attended the baseline or intervention sessions. Online Appendix Table F.2 shows the number of participants at each stage of the study.

### 2.3. Background on Chlorination Use

Child diarrhea is relatively high in our study area, as it is in many parts of the developing world. In the original WASH study control group, diarrhea prevalence in the past 7 days was 27% among children aged 1 and 2 (Null et al. 2018); in our sample at baseline, diarrhea prevalence was 16% in the past two weeks among children under age 3. Fecal contamination of drinking water is a likely cause of these episodes. Most of the population relies on communal water sources, usually wells with pumps or springs (Null et al. 2018). Women and children collect drinking water in plastic jerry cans and store it in jerry cans or pottery jars. Water can be (re)contaminated easily if storage containers are left open or when water is removed from storage pots.

Chlorinating water kills many of the pathogens that cause diarrhea.^14^ Absent point-of-collection chlorine dispensers, households can purchase dilute chlorine. The main brand is *WaterGuard*, which has been distributed and heavily marketed by the NGO *Population Services International* (PSI) in Kenya since 2003. *WaterGuard* is available in most local shops in the study area, and costs KES 25 (USD 0.25) per 150 ml bottle, about 0.2% of average monthly earnings. Each bottle treats 1,000 l of water (approximately one month to 50 days of household drinking water) and comes with instructions in Kiswahili and in pictures. There is widespread awareness of this product: even at baseline of the WASH study in 2012, 89% of the sample had heard of *WaterGuard*, and 29% had used it at least once (Null et al. 2018). Households also believe that chlorination reduces diarrhea.^15^

However, take-up of chlorination is low. In our sample during the unannounced home visits, 18% of pure control households had detectable free chlorine in their water and 22% had detectable total chlorine.^16^ A total of 66% of households report chlorination as one of their main methods of treating water (which may be true even if their current water is not chlorinated, or the chlorine present has decayed, see Section 4.1).

Chlorination may have monetary, time, attention, and disutility costs, in both dispenser and non-dispenser villages. Dispensers are not present at every water source, so people might need to plan to go to the correct water source. Once at the water source, they may face an attention cost, for example, when chatting with fellow villagers while fetching water. They might need to make alternative arrangements if the dispenser is not filled, or remind children fetching water to chlorinate it. In non-dispenser villages, women need to purchase *WaterGuard*, paying both time and monetary costs, and remember to add it to each batch of water.

Finally, taste has been mentioned as a potential barrier to chlorination (Ashraf, Berry, and Shapiro 2010; Dupas et al. 2016, 2020). One can taste if water has been chlorinated: In a blind taste test among staff of the Busara Center, participants correctly identified chlorinated drinking water 75% of the time. We find no statistically significant differences in the taste rating participants gave to chlorinated versus unchlorinated water.^17^ However, habit formation plays a large role in taste (Atkin 2013; Loewenstein, Price, and Volpp 2016). Taste habituation could be modeled as a standard investment choice, where people need to pay a fixed disutility cost to get used to the unfamiliar taste of chlorinated water. In this sense, taste could be seen as a cost barrier, rather than as an innate, fixed preference.

## 3. Interventions and Theory of Change

Here, we discuss the structure of interventions and the mechanisms they target. The structure of each session was held constant across treatment groups: each included a short lecture, followed by a story of a woman like them, reflections of how the themes relate to participants’ own lives, and finally exercises and activities. In all three active arms, the first intervention session concluded with the information module described in Section 3.3.

### 3.1. Treatment 1: Visualization + Information Module (“V+INF”)

The core of our Visualization intervention was to encourage participants to (a) connect their present behavior to outcomes in the future, (b) visualize alternative realizations of the future, depending on their current behavior, and (c) put themselves in the shoes of their future selves, imagine how they feel, and “talk” to them. The approach was deliberately visual and emotional, with participants asked to close their eyes repeatedly for several minutes, and to imagine their future selves in as much graphic detail as possible.

In the first intervention session, participants were given an interactive lecture on thinking about the future. Participants were asked to think of examples of small everyday behaviors (such as spending their leftover budget on snacks) that could affect future outcomes. The intervention carefully avoided changing participants’ beliefs about which present behavior would entail which future outcome—it merely encouraged them to make the connection themselves. Instead of prescribing a list of everyday behaviors, facilitators largely relied on examples brought up by the participants. The session then moved on to several silent visualization exercises, with prompts including “Close your eyes for one minute. Imagine the person you will be in one year. Imagine your family in one year,” and “Imagine that your future self can now talk to you. How does she feel? What does *she* want you to do?” In the second part of the session, participants listened to a story about a woman whose daily life is full of tasks and worries, and who consequently focuses only on what is necessary right now. Using examples of water chlorination and antenatal care visits, she learns over time that thinking about the future in her everyday actions helps her and her family to have a better life. The story was followed by a group discussion on behaviors from the participants’ own lives. The session concluded with participants visualizing and drawing an alternative realization of their own future, depending on present behavior.^18^ In the second session, participants revisited all concepts from the first session and repeated the visualization exercises, applied to different situations in their lives. Particular emphasis was put on how they could use future visualizations in their everyday life in order to overcome any temptations they may encounter. Unlike the Planning intervention, Visualization focused on high-level behaviors and outcomes without implementation details.

From a theoretical perspective, the Visualization intervention is based on the idea that present utility is easier to imagine than future utility. A substantial body of evidence in psychology shows that people imagine future events in much less detail than immediately upcoming events, focusing on abstract qualities rather than details of execution (Gilbert and Wilson 2009; Kahneman et al. 2004). In a recent theoretical contribution, Gabaix and Laibson (2017) formalize the idea of *as-if discounting*, which results from a perfectly patient decision-maker who forms beliefs about the utility from a future reward $u_{t}\sim \mathcal {N}(\mu ,\, \sigma _{u}^{2})$ by combining her prior *μ* with mental simulations of the reward $s_{t}$. Simulations are noisy, unbiased signals $s_{t}=u_{t}+\epsilon _{t}$ with $\epsilon _{t}\sim \mathcal {N}(0,\, \sigma _{\epsilon _{t}}^{2})$. Importantly, the simulation noise $var(\epsilon _{t})=\sigma _{\epsilon _{t}}^{2}$ increases in the time horizon *t*. Assuming for simplicity that $\mu =0$ (this can be relaxed), the average posterior belief of $u_{t}$ becomes $D(t)u_{t}$, where $D(t)=1/(1+{{\sigma _{\epsilon _{t}}^{2}}/{\sigma _{u}^{2}})}$ is the *as-if* discounting function. $D(t)$ decreases in *t*, and takes a hyperbolic shape when the simulation noise is linear in the time horizon ($\sigma _{\epsilon _{t}}^{2}=\sigma _{\epsilon }^{2}t$), thus generating present-biased behavior with preference reversals. The model implies that interventions that improve forecasting ability (and thus reduce simulation noise $\sigma _{\epsilon _{t}}^{2}$) will lead to more patient behavior. Gabaix and Laibson (2017) themselves note that forecasting precision is likely to improve with more time spent thinking about a trade-off, higher intelligence or imaginative capacity, and more effort spent forecasting.^19^ These predictions are matched by empirical evidence that patience in discounting tasks can be increased by introducing a deliberation period before subjects can make a choice (Imas, Kuhn, and Mironova 2022), and by letting subjects interact with age-progressed computer renderings of themselves while they choose (Hershfield et al. 2011).

We hypothesize that this intervention works by teaching participants to generate more precise utility forecasts when making intertemporal trade-offs in their daily lives, and thus act more patiently.^20^ In addition, we hypothesize that visualization exercises will strengthen the mapping between present behavior and future outcomes (self-efficacy). Applied (but not restricted) to the context of chlorination, this may mean that participants connect chlorinating in the present more to health benefits in the future, have a more precise image of these benefits, and thus weigh them more heavily against small present costs in terms of money, attention, or taste habituation.

### 3.2. Treatment 2: Planning + Information Module (“P+INF”)

The Planning intervention taught participants skills to help them undertake activities that they had identified as important but were struggling to do regularly. It is based on planning skills taught during a psychotherapy called “Behavioral Activation” (Ekers et al. 2014; Patel et al. 2017). Our exercises drew on an existing manual for implementing this approach (Richards and Whyte 2011).

BA draws on literature on instrumental reinforcement and motivation (Lejuez et al. 2011), which finds that people often avoid necessary tasks and even rewarding activities when mildly depressed or feeling demotivated. They thus lose out on the sense of achievement of accomplishing what they intended to do. Avoidance exacerbates negative moods, which further increases avoidance and rumination, creating a cycle of inactivity. BA teaches simple, structured skills to help people “get going” and re-engage with meaningful activities. Through simple diaries and planning exercises, they learn to set short-term goals, establish routines and reduce avoidance. Compared to other planning interventions, BA is distinctive in how it teaches people to structure plans to make them very easy to implement. For example, people are taught to start with the easiest tasks, so they quickly earn a sense of accomplishment, which improves mood and increases the likelihood they persist with the task. People are also taught to reward themselves (e.g. plan a fun activity) upon accomplishing a task.

The first session began with an interactive lecture of how anyone can get stuck in cycles of inactivity. Participants then listened to a story of a woman similar to them who was overwhelmed by basic chores, fell out of the habit of doing routine tasks, and struggled to find the motivation to start again. This had negative consequences for herself and her family. Fetching water and chlorinating it were used as examples. Participants could then share their experiences of similar situations.

Participants then did a series of worksheets. They identified activities in their daily lives that they felt were important, but where they were struggling to “get going”. They wrote two lists of activities they could do in the next week: one set they enjoyed doing, and one set that were necessary. They ranked the tasks on each list from most to least difficult, picked the easiest activities on each list, broke the tasks down into steps, and scheduled them in a weekly diary. In the second session, participants discussed barriers they had faced and brainstormed ways to overcome these barriers. Similar to the Visualization intervention, facilitators avoided prescribing what activities participants should do, but merely aimed to teach skills to help them achieve their priorities.

We hypothesize that the planning intervention helps participants to make and implement plans for daily necessary tasks (including but not restricted to chlorination). We also hypothesize that concrete experiences of executing one’s plans and achieving a desired goal—“mastery experiences”—will increase self-efficacy, consistent with the psychological literature (Bandura 1997; Lorig et al. 2014).

### 3.3. Treatment 3: Active Control Exercise + Information Module (“AC+INF”)

The “active control” intervention controls for any effects of simply attending a session and interacting with women from neighboring villages. The sessions followed the format of the two treatment interventions. The content of these sessions centered on the birds and plants of Kenya, topics that did not capture any of the psychological elements contained in the Visualization or Planning modules. In the first session, participants listened to a short lecture on different kinds of birds that live in Kenya, followed by a short story about the daily routine of a woman similar to them. Participants discussed the birds they see in their village. They wrote a list of all the birds they could think of, and then made some drawings of birds. The second session, centered on plants in Kenya, followed an identical structure, except that it did not include another short story about a woman, in line with the second sessions of the other treatments.

All three intervention groups concluded with an information module about the benefits of chlorination. Participants were read information on chlorination, as well as on antenatal care and postnatal care (ANC/PNC). The chlorination message stated that only water properly treated with chlorine or boiled water is safe to drink, while unsafe water can cause illness. It noted that children are the most affected by diarrhea, which causes dehydration and can impede growth. It highlighted that people could avoid one out of three episodes of diarrhea by chlorinating with products like *WaterGuard* or dispenser chlorine. The message gave instructions on chlorination dosage and the importance of covering water containers. It highlighted that the smell of chlorine is not harmful and reduces over time, and that chlorinating is cheaper than the costs of firewood for boiling.

## 4. Outcome Measures

We have two families of outcomes, behaviors and psychological mechanisms. Within each family, we pre-specified primary, secondary, or exploratory hypotheses, and the variables used to test each hypothesis, outlined in Online Appendix Table F.1. We use this ranking in correcting for multiple testing, as described in Section 5.

### 4.1. Health-Related Outcomes

Our primary hypothesis is that the interventions affect whether households chlorinate water. In the short-term, we conduct objective tests of the household’s drinking water in unannounced household visits as well as self-reported measures of how households treat water in the 10-week survey. In the 33-month survey, we repeat the self-reported measure.

In the short-term, our primary outcome is an indicator for whether the Total Chlorine Residual (TCR) is above 0.2 mg/l in household drinking water. This captures whether households have chlorinated water in the past 24–72 hours. The measure is a lower bound on whether households have chlorinated this water at any point: TCR decays and disappears, at different rates depending on the type of container and how much organic material is present in the water, and thus may not be present in water that has been chlorinated more than 24 hours ago (Murphy et al. 2016; Null and Lantagne 2012).^21^ We additionally measure whether the Free Chlorine Residual (FCR) level is above 0.2 mg/l. Put simply, FCR is an alternative, slightly more stringent measure for water safety (WHO 2011). Like some other studies in economics (Dupas et al. 2016; Kremer et al. 2011a), we focus on TCR. In the health literature, TCR is increasingly preferred to FCR because it is more strongly correlated with the presence of *E. coli* (Murphy et al. 2016), the bacteria that causes diarrhea.

In surveys, we asked households whether and how they generally treat water and recorded up to two treatments. An indicator for answering “chlorine” is our primary outcome at 33 months. We minimize demand effects through question administration. In the baseline and 10-week survey, the module is self-administered on a tablet, so enumerators do not see answers. Water treatment options are presented as buttons on a tablet screen and include ineffective options like straining or letting it settle. In the 33-month survey, this question is asked in open format on the phone, without prompting any options. Enumerators coded participants’ answers. In all surveys, this was the first mention of water treatment.

We view the objective measure as a lower bound on the use of chlorine and the self-report as an upper bound. Importantly, they are not directly comparable: The objective measure captures whether current water is treated, while the self-report captures general behavior. Both measures have strengths and weaknesses, so many health studies measure both (Murphy et al. 2016). The objective measure minimizes experimenter demand effects, but may not capture households who have added chlorine some time ago and may still have safe water. The self-reported measure is better suited to capture broader chlorination behavior and also covers boiling of water. However, it is more susceptible to experimenter demand effects. We discuss such concerns further in Section 4.4.

We find TCR present in current drinking water for 23% of active control households and FCR present for 21% at the 12-week chlorine test. A total of 73% of households report generally using chlorine. Other studies also find gaps between self-reports of whether water is *currently* chlorinated and water tests: Blanton et al. (2010) find self-reported chlorine use in current water of 39% and positive tests of 21% in Kenya; Gupta et al. (2007) find 81% and 50% in Indonesia.

We test whether interventions affect health outcomes related to chlorination and other preventive health behaviors. We examine self-reported child diarrhea as an exploratory outcome in the 10-week survey and a primary outcome in the 30–36-month survey. For each child in the household, we ask about the number of independent episodes of diarrhea over the last three months. We also measure other preventive health behaviors, including vaccinations and health check-ups.

### 4.2. Savings, Labor Supply and Other Economic Outcomes

The ability to visualize future benefits and planning skills are relevant for many behaviors. Our secondary hypothesis for behavioral variables is that our interventions (which were domain-general) affect other investment behaviors, in particular savings, education, and labor supply. During both short-run and long-run surveys, participants therefore completed several modules on economic behaviors. We pre-specified secondary outcomes measuring savings and labor supply in both rounds, as well as an index of investment in children’s education in the 10-week follow-up.

### 4.3. Targeted Psychological Channels

We hypothesized three mechanisms for the effect of our interventions on behavior: The Visualization intervention targeted time preferences and self-efficacy, while the Planning intervention targeted planning skills and self-efficacy. We use incentivized choice tasks or validated psychological scales to measure these constructs.^22^

#### Planning Skills.

Our first primary hypothesis in relation to mechanisms is that the intervention alters whether people make plans and follow through on them. The primary outcome is a short form of the Behavioral Activation for Depression Scale (BADS) (Kanter et al. 2007; Manos, Kanter, and Luo 2011), capturing agreement with items about setting and following through on goals (e.g. “I was an active person and accomplished the goals I set out to do”). As a secondary outcome, we conduct an incentivized Tower of London (ToL) task, which measures a participant’s higher-order cognitive ability to plan ahead in sequential strategies (Phillips et al. 2001; Shallice 1982). Online Appendix G.1 provides further details.

#### Time Preferences and Utility Forecasting.

Our second primary hypothesis is that the intervention affects time preferences. In the 10-week survey, we estimate time preferences in the effort domain, following recent innovations in the elicitation of time preferences (Andreoni and Sprenger 2012; Augenblick, Niederle, and Sprenger 2015), and noting that water chlorination is an effortful task. We use the methodology of Augenblick (2017), and implement it with a newly developed effort task, adapted to a field setting without computer or smartphone access. Each effort task consisted of sending a 30-digit random number string by SMS to a toll-free number.^23^ Participants choose how many SMS they want to send at a time *t* for a piece rate *w*, where *t* is 0, 1, 7, or 8 days from today, and the piece rate *w* is KES 2, KES 6, or KES 10. We structurally identify a present bias parameter ($\beta$), the primary outcome, as well as an impatience parameter ($\delta$), assuming quasi-linear utility and a power cost of effort function (Augenblick 2017; DellaVigna and Pope 2017). In addition to the effort discounting task, we include monetary discounting parameters from a conventional MPL (Andreoni and Sprenger 2012) as secondary outcomes. Delays included today, 4 weeks, and 8 weeks. Online Appendices G.2 and G.3 provide full details on the estimation. In the long-run survey, we administer a shorter form of an MPL using the “staircase procedure” and a qualitative question on patience from the Global Preference Survey (GPS) (Falk et al. 2018).

In the long-run follow-up, we designed a measure to capture how well people can form a precise mental image of their future, adapted from the Plymouth Sensory Imagery Questionnaire (Andrade et al. 2014). We asked respondents to form an image in their mind of themselves and their family in one year, and then rate the clarity and vividness of the image. This measure proxies for the utility forecasting noise $\sigma _{\epsilon _{t}}^{2}$ in Gabaix and Laibson (2017) (see Section 3.1).^24^ It is our primary psychological outcome in the long-run survey (along with self-efficacy, below). We discuss how our measures of intertemporal choice relate to each other in Section 7.

#### Self-Efficacy.

Our final potential mechanism is self-efficacy, the belief that one is able to achieve desired outcomes (see footnote 3). We use a widely used psychological scale, the Generalized Self-Efficacy (GSE) scale (Schwarzer and Jerusalem 2010). Participants rate their agreement (on a scale from 1 to 5) with 12 statements such as “I always manage to solve difficult problems if I try hard enough” and “It is easy for me to stick to my aims and accomplish my goals.” Responses are aggregated into a summary score and *z*-scored.

### 4.4. Alternative Mechanisms

We also measure alternative mechanisms and potential confounds. First, we measure beliefs and knowledge about chlorination. The addition of the information module to all active arms aims to ensure these are held constant. We verify this using multiple-choice questions referring to the information module. We measure participants’ beliefs about the effectiveness of chlorination in preventing pediatric diarrhea. We also measure knowledge about how to properly use chlorine with questions about how much chlorine to add to water and how much time needs to pass after adding chlorine until the water is safe to drink.^25^

Second, we test whether our interventions differentially focus participants’ attention on chlorination. We measure the salience of three future-oriented behaviors (chlorination, savings, and farm investment) compared to non-future oriented behaviors. Enumerators read out three lists of nine words each to every participant, and then asked her to recall as many words as possible after reading each list (participants were paid KES 5 for every word they remembered). Each list contained three categories of future-related words (chlorine, savings, and farm investment), as well as non-future related filler words (see Online Appendix Table G.2). The task captures whether a concept is “top-of-mind” and is conceptually similar to the audio word search task in Lichand and Mani (2020). We test whether our treatments differentially affect the probability to recall chlorine words, *conditional* on the total number of words remembered. See Online Appendix G for full specification details.

Third, we measure risk preferences using a modified Eckel–Grossman task, which offered a choice between one of three 50/50 lotteries, represented as bets on a coin flip (Charness, Gneezy, and Imas 2013). One participant per 25 people was paid their choice.

## 5. Econometric Approach

### 5.1. Main Specification

Our main specification compares the two “psychological” treatment groups, V+INF and P+INF, to the active control group, AC+INF. We employ the following specification, separately for data collected 10 weeks and 33 months after the first intervention session:

(1) \begin{equation*} y_{i1}=\alpha _{0}+\alpha _{1}T_{1i}+\alpha _{2}T_{2i}+\phi X_{i}+\delta y_{i0}+\gamma _{v}+\varphi _{w}+\rho _{d}+\theta _{a}+\eta _{i}, \end{equation*}

where $y_{i1}$ is the outcome of interest for respondent *i* at time of endline. $T_{1i}$ and $T_{2i}$ are dummies equal to one if the respondent is assigned to the “Visualization” or “Planning” group, respectively. $X_{i}$ is a vector of participant controls (year of birth, employment status, marital status, and education level). $\gamma _{v}$ are village fixed effects. $\varphi _{w}$ and $\rho _{d}$ are fixed effects for the survey week and weekday.^26^$\theta _{a}$ is an indicator for household asset wealth greater than the sample median (used for stratification). Standard errors are clustered by intervention cohort (five participants) to account for within-group dynamics. For variables collected at baseline (Online Appendix Table F.3), we include $y_{i0}$, the outcome at baseline.^27^ We winsorize outcome variables with no theoretical lower and upper bounds at the 1st and 99th percentiles. As pre-specified, the sample includes all participants who completed both the relevant survey (after 10 weeks or 33 months) and the combined baseline and first intervention session, and thus excludes the pure control group. Selection into the sample based on treatment is not possible because the nature of the intervention was not revealed before the first intervention session.

### 5.2. Multiple Hypothesis Testing

We calculate two sets of *p*-values: “per-comparison” *p*-values, which are appropriate for readers interested in a specific outcome, and sharpened “*q*-values” (Benjamini, Krieger, and Yekutieli 2006), which adjust *p*-values for the false discovery rate (FDR) among groups of outcomes. We adjust for multiple hypothesis testing within outcome groups (behaviors and psychological mechanisms) and hierarchical outcome categories (primary, secondary, and exploratory), but not across them. We consider the effects of our two active interventions to be theoretically distinct and therefore do not correct across them. Online Appendix Table F.1 shows the hypothesis under which a given variable falls in each survey round. Indices are constructed following Anderson (2008).

### 5.3. Comparison with the Pure Control Group

In Online Appendix E, we also report results from comparing the three active arms (V+INF, P+INF, and AC+INF) to a pure control group (PC). As pre-specified, the sample includes all recruited participants who completed the respective survey (10 weeks or 33 months), including “non-compliers” who were assigned to the active treatment groups, but chose not to participate in the baseline survey or the interventions. The specification is identical to that in equation (1), except that there is a third treatment indicator $T_{3i}$ for the active control group, the pure control group is used as the reference category, and the estimation does not control for the baseline outcome $y_{i0}$. This comparison gives the total effect on targeted behaviors of providing interventions such as ours in other, similar settings. In contrast, the active control comparison isolates the effects of the psychologically active elements of Visualization and Planning. The information module, exposure to field staff and other participants, and experimental payments are held constant across the three active arms and the restriction to baseline survey participants holds constant any practice effects on tasks.

## 6. Results

### 6.1. Experimental Integrity

Table 1 shows randomization balance, differential survey participation, intervals between surveys, and compliance with treatment. The first panel shows that demographic variables are balanced across treatments, with only one out of 25 coefficients reaching statistical significance at the 10% level. Online Appendix Table F.3 shows that baseline outcomes are balanced prior to interventions.

The second panel shows participation in each round. Defining attrition as a failure to complete the 10-week or 33-month surveys among those who attended baseline (the standard definition of attrition for most experiments), average attrition in the active control group was 8% in the 10-week endline, 12% in the chlorine test, and 10% in the long-run follow-up. Attrition is balanced across the active control, Planning and Visualization groups. Demographic variables predict attrition from all survey rounds, but not in interaction with treatment status, suggesting that the composition of the sample remains similar between groups in all rounds (Online Appendix Tables F.5 and F.6).

We put more weight on the active control comparison, where attrition by treatment group is balanced, than the pure control comparison. In the pure control comparison, there is more drop-out and there are some differences in participation in the endline and the chlorine test across groups. In the pure control group, 24% of people *in the recruitment census* did not complete the endline, 26% did not complete the chlorine test, and 17% did not complete the follow-up. Non-participation in surveys is higher as we cannot condition on baseline attendance in this sample, and thus include all participants from the recruitment census. The difference is important: The recruitment census was conducted door-to-door in the villages and includes all women who meet our sampling criteria (Section 2.1). The baseline and 10-week survey were conducted in mobile labs, typically 30 minutes from participants’ homes. Thus, the 24% of the pure control group who fail to participate in the endline are not “attriters” in the conventional sense that they choose to drop out of the study—they are individuals who choose not to participate in the first place (perhaps because the mobile lab is too far or they do not have childcare).

The third panel in Table 1 shows some small imbalances in the delay between the date of the baseline (and first intervention) to the subsequent rounds. Relative to the active control group, the Planning group completed the endline survey and chlorine test about 2 days later. We include a fixed effect for the survey week and survey weekday, for all outcomes in the short- and long-run survey (see equation (1)). We present estimates without these fixed effects in Online Appendix Tables H.3 and H.4, and the results are robust.

The final panel in Table 1 shows compliance rates, which are balanced across the treatment groups. After the census, all respondents in the active arms were invited to the baseline and first intervention session, which were held on the same day. A total of 78% of respondents attended the baseline and completed the first session, and 74% of completed both sessions.

### 6.2. Health-Related Outcomes

Table 2 shows results on behavioral outcomes, with health outcomes shown in the top panel. The column “Multiple Hypothesis Test (MHT) level” indicates whether variables were pre-specified as primary (1), secondary (2), or exploratory outcomes (3), or were not pre-specified (np). The first number relates to the outcome pre-specification in the 10-week survey, and the second number relates to the outcome pre-specification in the 30–36-month survey. The ranking of variables is given in Online Appendix Table F.1.

**Table 2.** Behavioral outcomes.

	Endline (10–12 weeks)					Follow-up (30–36 months)					
		(1)	(2)	(3)	(4)	(5)	(6)	(7)	(8)	(9)	(10)
	MHT level	Active control group mean (SD)	Visualization treatment effect	Planning treatment effect	Column 2 versus Column 3 *p*-value	*N*	Active control group mean (SD)	Visualization treatment effect	Planning treatment effect	Column 2 versus Column 3 *p*-value	*N*
*Health outcomes*											
Objective measure: chlorine present in water (TCR)	1/-	0.23	0.05	0.02	0.15	2,012					
		(0.42)	(0.02)$^{**}$	(0.02)							
Objective measure: chlorine sufficient to be safe (FCR)	3/-	0.21	0.04	0.01	0.16	2,012					
		(0.40)	(0.02)$^{**}$	(0.02)							
			[0.04]$^{**}$	[1.00]							
Main treatment: chlorine (self-report)	np/1	0.73	0.07	-0.00	0.00$^{***}$	2,116	0.85	0.05	0.02	0.15	2,073
		(0.45)	(0.02)$^{***}$	(0.02)			(0.35)	(0.02)$^{***}$	(0.02)		
			[0.00]$^{***}$	[1.00]				[0.03]$^{**}$	[0.39]		
Main treatment: boil (self-report)	np/np	0.35	0.07	0.05	0.46	2,116	0.63	0.03	-0.01	0.19	2,073
		(0.48)	(0.03)$^{***}$	(0.03)$^{*}$			(0.48)	(0.03)	(0.03)		
			[0.01]$^{**}$	[0.39]				[1.00]	[1.00]		
Diarrhea incidences per child u15, last 3 months	3/1	0.26	-0.12	-0.06	0.09$^{*}$	2,004	0.27	0.00	-0.04	0.26	2,045
		(0.69)	(0.03)$^{***}$	(0.03)$^{*}$			(0.73)	(0.04)	(0.03)		
			[0.00]$^{***}$	[0.39]				[0.32]	[0.39]		
Diarrhea incidences per child u5, last 3 months	np/3	0.34	-0.16	-0.06	0.02$^{**}$	1,682	0.39	0.01	-0.01	0.70	1,612
		(0.86)	(0.05)$^{***}$	(0.05)			(1.04)	(0.07)	(0.06)		
			[0.00]$^{***}$	[1.00]				[1.00]	[1.00]		
Proportion of children taken for healthcare check-up	3/3	0.21	-0.04	-0.02	0.34	1,995	0.36	-0.02	-0.01	0.88	2,045
		(0.34)	(0.02)$^{**}$	(0.02)			(0.37)	(0.02)	(0.02)		
			[0.03]$^{**}$	[1.00]				[1.00]	[1.00]		
Proportion of children u15 vaccinated, last 3 months	3/-	0.22	0.00	-0.01	0.49	1,990					
		(0.35)	(0.02)	(0.02)							
			[0.38]	[1.00]							
Number of ANC visits, last 3 months (if pregnant)	3/-	1.26	-0.10	0.21	0.45	200					
		(1.19)	(0.49)	(0.36)							
			[0.38]	[1.00]							
*Savings outcomes*											
Amount saved regularly (per week, KES)	2/2	93.96	24.37	3.58	0.12	2,108	407.50	56.90	23.66	0.34	2,073
		(230.26)	(12.38)$^{**}$	(12.55)			(605.28)	(33.78)$^{*}$	(33.29)		
			[0.18]	[1.00]				[0.12]	[0.58]		
Indicator: amount saved regularly is positive	3/3	0.36	0.13	-0.02	0.00$^{***}$	2,108	0.78	0.05	0.02	0.13	2,073
		(0.48)	(0.03)$^{***}$	(0.03)			(0.42)	(0.02)$^{**}$	(0.02)		
			[0.00]$^{***}$	[1.00]				[0.20]	[1.00]		
Number of ROSCAs [joined in last 3 months/total]	3/3	0.17	0.04	0.01	0.24	2,108	1.08	0.12	0.10	0.75	2,073
		(0.44)	(0.03)	(0.02)			(1.05)	(0.06)$^{*}$	(0.06)		
			[0.08]$^{*}$	[1.00]				[0.29]	[1.00]		
Weekly ROSCA savings	np/np	205.93	32.44	11.22	0.17	2,108	246.07	41.36	33.23	0.72	2,073
		(304.72)	(15.73)$^{**}$	(16.35)			(363.71)	(22.13)$^{*}$	(21.21)		
			[0.04]$^{**}$	[1.00]				[0.12]	[0.47]		
Indicator: saves for productive investments	3/3	0.17	0.11	-0.01	0.00$^{***}$	2,108	0.62	0.02	0.01	0.84	2,073
		(0.38)	(0.02)$^{***}$	(0.02)			(0.49)	(0.02)	(0.03)		
			[0.00]$^{***}$	[1.00]				[1.00]	[1.00]		
Total savings balance (KES)	-/3						2542.85	1066.69	39.07	0.01$^{***}$	2,073
							(6378.76)	(428.62)$^{**}$	(404.17)		
								[0.20]	[1.00]		
*Labor outcomes*											
Total hours of work [last 3 months/ last 7 days]	2/2	106.11	-5.38	-23.39	0.05$^{*}$	2,108	13.25	2.36	0.89	0.09$^{*}$	2,073
		(174.61)	(9.54)	(8.93)$^{***}$			(15.41)	(0.88)$^{***}$	(0.87)		
			[0.85]	[0.03]$^{**}$				[0.06]$^{*}$	[0.47]		
Total days of work, last 3 months	3/-	21.22	-0.31	-3.70	0.04$^{**}$	2,108					
		(30.09)	(1.62)	(1.58$^{**}$							
			[0.38]	[0.37]							
Earnings, cash and in-kind [monthly/last 7 days]	3/3	1094.50	0.17	10.16	0.94	2,108	677.26	104.62	101.91	0.97	2,073
		(2865.35)	(147.14)	(163.57)			(1343.83)	(77.27)	(80.34)		
			[0.40]	[1.00]				[0.94]	[1.00]		
*Other behavioral outcomes*											
Index of investment in children’s education (*z*-score)	2/-	0.00	-0.02	0.00	0.68	1,420					
		(1.00)	(0.06)	(0.07)							
			[0.85]	[1.00]							

Notes: OLS estimates of treatment effects. For each variable, columns (1) and (6) report the mean and standard deviation of the control group. Columns (2) and (3) and columns (7) and (8) report the coefficients of interest and standard errors in parentheses, respectively. Square brackets contain *p*-values corrected for MHT using the FDR. The column ``MHT level'' indicates the MHT level: whether variables were pre-specified as primary (1), secondary (2), or exploratory outcomes (3), or were not pre-specified (np). The first number relates to pre-specification in the 10-week survey; the second relates to the 30–36-month survey. All regressions include village-level fixed effects, controls for individual characteristics, week and day-of-week fixed effects and standard errors clustered at the level of the intervention cohort. The sample in all regressions is restricted to participants in active treatment groups who attended the baseline survey. Where available, we control for the baseline outcome of the dependent variable. Outcome measures are described in Section 4.

*denotes significance at the 10%, ^**^at the 5%, and ^***^at the 1% levels.

#### Chlorination.

After 12 weeks, the Visualization intervention led to a significant increase in our primary behavioral outcome measure, the presence of chlorine in household drinking water, measured during unannounced household visits. Among the Visualization group, there is a 5 percentage point (22%) increase in the presence of TCR, significant at the 5% level, relative to an active control mean of 23%. There is a small, statistically insignificant increase on chlorination in the Planning group. We cannot reject that the Visualization and Planning interventions have the same effect ($p=0.15$). The results for FCR are similar in magnitude and statistical significance. Both results are indicative of increased safety of household drinking water.

We see a similar pattern of treatment effects on a self-reported measure of whether households chlorinate water after 10 weeks and 30–36 months. We asked households whether and how they generally treat water and recorded up to two treatments (see Section 4.1). In the Visualization arm, the percentage of respondents who report generally chlorinating water increases by 7 percentage points after 10 weeks and by 5 percentage points after 30–36 months. Both effects are significant at the 1% level. We see no significant effects in the Planning arm in either round. We can reject that the Visualization and Planning interventions have the same effect in the 10-week survey, but not in the long-run follow-up ($p=0.15$). Our findings are consistent with studies of long-term effects of point-of-use chlorination, which find chlorine use becomes a habit once households have learned to chlorinate and, potentially, become accustomed to taste (Luby et al. 2006, 2004).

We also find that our treatments increase boiling of water after 10 weeks, which suggests that increased chlorination did not crowd out other effective methods of water treatment. (Boiling was mentioned in our information module, see Section 3.3.)^28^ We find little evidence that the 2020 pandemic affected water treatment practices: A total of 97% of households report treating their water in the same way than they did before March 2020.

#### Other Health Outcomes.

The Visualization and Planning treatments both generate large and statistically significant reductions in the incidence of diarrhea among children after 10 weeks. Relative to the active control group, we find a 46% (0.12 episodes) reduction in diarrhea episodes per child under 15 in the last three months for Visualization and a 23% (0.06 episodes) reduction for Planning.^29^ However, only the effect of Visualization survives multiple test corrections. We can reject that the Visualization and Planning treatments are equally effective in reducing diarrhea ($p=0.09$). We find similar effects on a non-prespecified outcome, diarrhea per child under 5, although only the Visualization effect is statistically significant. We find no effects on other health outcomes, including vaccinations and pre-natal care visits, with the exception of a small decrease in the number of children under 15 who visited a healthcare provider in the last three months in the Visualization group, potentially due to reduced diarrhea incidence.

After almost 3 years, there is little persistence in the effects on diarrhea. One possible reason is that the short-run effects on boiling do not persist in either group. We also find suggestive evidence that the difference in short and long-run diarrhea results is linked to the seasonality of diarrhea in Kenya (Chao et al. 2019) and the fact that the surveys are in different seasons. Water chlorination is more likely to affect diarrhea in the rainy season than in the dry season.^30^ The 10-week survey was conducted in February and March 2018, during rainy season. The 33-month survey ran from July to December 2020, with the majority of surveys conducted during dry season. Online Appendix Table D.1 shows treatment effect heterogeneity by whether the survey is conducted during rainy season (November–December 2020) and by whether the household’s water source is protected.^31^ We find no effects of our interventions during the dry season, but some effects during rainy season. During the rainy season, Visualization decreases the probability that any child under 15 in the household had diarrhea in the last 7 days by 10 percentage points, though we lack power to detect significance. This effect increases to 34 percentage points ($p<0.05$) for those whose water source is not protected.

The short-run treatment effects of our interventions on diarrhea are rather large in relation to the effects on chlorination; with an effect on diarrhea of $-46$% and an effect on the presence of any chlorine of $+22$% in the Visualization arm. A back-of-the-envelope calculation suggests an instrumental variable estimate of $-2.09$%, that is, a 1% increase in chlorination leads to a 2.09% reduction in diarrhea episodes. This estimate is within the confidence interval of a recent meta-analysis, which considers effects of dispensers or distribution of free chlorine (Arnold and Colford 2007).^32^ There are a few possible reasons for our relatively large effects. First, our interventions affect both chlorination and boiling of water. Second, they are domain-general trainings that may affect other behaviors related to child diarrhea, such as handwashing, washing fruit and vegetables, open defecation and general hygiene. Third, there could be dependencies with the treatment effects on non-health outcomes, such as the observed increases in savings (described below).

#### Concerns About Demand Effects in Chlorination Measures.

Our objective chlorination measures, obtained during unannounced household visits two weeks after the 10-week survey, leave little room for experimenter demand effects, such as social desirability bias in responding to survey questions. It was also difficult for households to anticipate our visits for chlorine tests. Visits were concentrated in time within each village: 68% of chlorine tests were conducted on the first day that our team visited a given village (the remaining tests were spread out, with a median within-village range of 7 days). Online Appendix Table H.2 shows that the estimated treatment effects on chlorine are unaffected by including a dummy for being tested on the first day within one’s village, or the number of days elapsed since the first chlorination tests were conducted in the village. When we include interactions, we find that participants in the Visualization group are (insignificantly) *more* likely to have chlorine in their water if tested on the first day in their village—the opposite of what would be expected if knowledge about chlorination visits spreads. Finally, we see effects on child diarrhea in the 10-week endline survey, two weeks before the chlorine test, suggesting increases in chlorination had already occurred. For the same reason, it is unlikely that our treatment effects are driven by the 10-week survey itself acting as a reminder to chlorinate by the 12-week test. First, we see effects on diarrhea and self-reported chlorination in the 10-week survey. Second, questions about water treatment are included in surveys to all four groups. Third, we do not see an effect on the active control group, suggesting the survey does not remind participants of our information module on chlorination (Online Appendix Table E.1).

Concerns about demand effects may be more plausible in our self-reported measures of chlorination. Treatment effects across the objective and self-reported measures are strikingly consistent, which is suggestive evidence that households are reporting truthful answers. To further investigate the possibility of experimenter demand effects in self-reports, we include randomized demand treatments (de Quidt, Haushofer, and Roth 2018) in the follow-up survey (at the end, orthogonal to our psychological treatments), which reveal the objective of the experimenter to the participants.^33^ This has a precisely estimated zero effect on self-reported chlorination behavior (difference of means $-0.006$ SD, $p=0.92$, see Online Appendix Table H.1). Thus, while we do not rule out demand effects in self-reports, we do not find any evidence for them.

### 6.3. Savings, Labor Supply and Other Economic Outcomes

The second panel of Table 2 shows effects on savings-related outcomes. After 10 weeks, the Visualization treatment leads to a significant increase in our main savings-related outcome variable, the amount of money saved regularly (converted to savings contributions per week). This effect corresponds to a 26% (KES 24) increase relative to the active control group, significant at the 5% level. After 3 years, the treatment effect on weekly savings is 14% (KES 57).^34^

In addition, we find large and significant effects on all pre-specified savings outcomes in the Visualization group, relative to the active control group. Savings on the extensive margin increase, with the share of respondents who save regularly increasing by 13 percentage points (36%) after 10 weeks and by 5 percentage points (7%) after 3 years.^35^ Similarly, we find increases in the Visualization group on an indicator for whether the respondent saves for productive investments (education, business, agriculture) after 10 weeks, although this effect does not persist. Visualization participants have joined more ROSCAs in both rounds. Their weekly savings contributions to ROSCAs increase by KES 32 (16%) after 10 weeks and KES 41 (19%) after 3 years (see footnote 34 on how this relates to overall savings).

After almost 3 years, the Visualization group has accumulated 41% higher savings balances. The long-term survey occurred during the 2020 global pandemic, an economic shock which reduced real income in rural Kenya by 7.9% (Nechifor et al. 2020). Our results may suggest that participants who learned visualization techniques in 2017 benefited from an increased financial buffer in 2020.^36^ There are no significant effects of the Planning treatment on savings. Together, these results show that the Visualization treatment strongly affected savings behavior and effects persist over time.

The third panel of Table 2 reports effects on labor-related outcomes. Somewhat surprisingly, we find a reduction of 23 hours (22%) in the total number of hours worked in the last three months in the Planning group, and a similar effect on the number of days worked. The Planning group may have improved their daily schedule of activities and been more efficient. Respondents work mainly in subsistence farming or self-employment, and thus their income is determined largely by their output rather than their work hours. Indeed, we find no significant change in monthly earnings, despite the shortened work hours. However, the effect is temporary and completely disappears by the 3 year follow-up. In contrast, we find a highly significant positive effect of Visualization on hours worked in the long run, of 2.4 hours worked (18%) in the last 7 days. The Visualization intervention focused on the future in one year, and some participants may have used this to develop longer-term career visions, including applying for salaried jobs. However, these longer-term visions may not have affected labor supply in the short term. We see no effects on an index of children’s education investment after 10 weeks and did not measure it in the long-term follow-up.

Our findings on non-health behaviors have two important implications. First, our Visualization intervention strongly affected future-oriented behaviors across different domains, in both short- and long-run. We discuss evidence on potential mechanisms in Section 7. Second, it provides further evidence against experimenter demand effects: While the interventions mentioned chlorination and health-related topics, savings and work were discussed only to the extent that participants brought them up themselves in the discussions. The treatment effects we report here are thus more likely to result from an increased valuation of the future rather than from a simple desire to please the experimenters.

### 6.4. Treatment Effects When the Costs of Chlorination Vary

We examine whether our treatment effects differ by whether or not the village in which the interventions took place was randomly allocated chlorine dispensers in the WASH Benefits study.^37^ Dispensers reduce the monetary and non-monetary costs of chlorination, by providing free chlorine at specific water sources, and by reminding people to chlorinate when collecting water. Chlorination rates are slightly higher in dispenser than in non-dispenser villages, both in objective and self-reported data, and use of boiling is slightly lower (Table 3). For young children, rates of diarrhea are slightly higher in non-dispenser than in dispenser villages.^38^

**Table 3.** Chlorine-related outcomes in dispenser versus non-dispenser villages.

	Village has no chlorine dispenser				Village has chlorine dispenser				Comparison	
	(1)	(2)	(3)	(4)	(5)	(6)	(7)	(8)	(9)	(10)
	Active control mean (SD)	Visualization treatment effect	Planning treatment effect	*N*	Active control mean (SD)	Visualization treatment effect	Planning treatment effect	*N*	Visualization interaction *p*-value	Planning interaction *p*-value
**Endline: 10–12 weeks**										
Objective measure: chlorine present in water (TCR)	0.19	0.05	0.04	1,082	0.29	0.04	-0.00	930	0.87	0.34
	(0.39)	(0.03)$^{*}$	(0.03)		(0.45)	(0.03)	(0.03)			
Objective measure: chlorine sufficient to be safe (FCR)	0.16	0.05	0.03	1,082	0.27	0.03	-0.01	930	0.60	0.32
	(0.36)	(0.03)$^{**}$	(0.03)		(0.44)	(0.03)	(0.03)			
Main Treatment: chlorine (self-report)	0.65	0.09	0.02	1,129	0.82	0.06	-0.01	987	0.34	0.49
	(0.48)	(0.03)$^{***}$	(0.03)		(0.39)	(0.03)$^{*}$	(0.03)			
Main Treatment: boiling (self-report)	0.37	0.06	0.04	1,129	0.33	0.09	0.07	987	0.52	0.56
	(0.48)	(0.04)	(0.04)		(0.47)	(0.04)$^{**}$	(0.04)$^{*}$			
Diarrhea incidences per child u15, last 3 months	0.26	-0.10	-0.04	1,066	0.25	-0.13	-0.09	938	0.69	0.37
	(0.67)	(0.05)$^{**}$	(0.05)		(0.71)	(0.05)$^{**}$	(0.05)$^{*}$			
Diarrhea incidences per child u5, last 3 months	0.37	-0.15	-0.05	908	0.30	-0.15	-0.06	774	0.97	0.93
	(0.88)	(0.06)$^{**}$	(0.07)		(0.83)	(0.06)$^{**}$	(0.07)			
**Follow-up: 30–36 months**										
Main treatment: chlorine (self-report)	0.81	0.05	0.02	1,103	0.90	0.04	0.03	970	0.78	0.78
	(0.39)	(0.03)$^{*}$	(0.03)		(0.30)	(0.02)$^{**}$	(0.02)			
Main treatment: boiling (self-report)	0.63	0.04	0.02	1,103	0.62	0.02	-0.03	970	0.67	0.37
	(0.48)	(0.04)	(0.04)		(0.49)	(0.04)	(0.04)			
Diarrhea incidences per child u15, last 3 months	0.29	0.01	-0.10	1,088	0.25	-0.04	-0.02	957	0.48	0.23
	(0.71)	(0.06)	(0.05)$^{**}$		(0.76)	(0.06)	(0.05)			
Diarrhea incidences per child u5, last 3 months	0.46	-0.02	-0.09	867	0.32	0.02	0.06	745	0.74	0.15
	(1.12)	(0.09)	(0.09)		(0.93)	(0.09)	(0.08)			
Chlorine source: bottle	0.47	0.06	0.04	1,103	0.31	0.02	0.06	970	0.48	0.77
	(0.50)	(0.04)	(0.04)		(0.46)	(0.04)	(0.04)			
Chlorine source: dispenser	0.21	-0.04	-0.04	1,103	0.54	-0.01	-0.05	970	0.57	0.93
	(0.41)	(0.03)	(0.03)		(0.50)	(0.04)	(0.04)			
Reports working dispenser at main source	0.34	-0.03	0.01	1,103	0.70	0.03	-0.04	970	0.17	0.33
	(0.47)	(0.04)	(0.04)		(0.46)	(0.03)	(0.03)			
Reports working dispenser within 30 minutes walk	0.49	-0.01	-0.02	1,103	0.82	0.01	-0.01	970	0.57	0.77
	(0.50)	(0.04)	(0.04)		(0.38)	(0.03)	(0.03)			

Notes: OLS estimates of treatment effects. Columns (1) and (5) report the mean and standard deviation of the control group. Columns (2) and (3) and columns (6) and (7) report the coefficients of interest and standard errors in parentheses, respectively. The analysis repeats that in Table 2, separately for villages which randomly received a chlorine dispenser in the WASH study and for villages which did not receive chlorine dispensers. We use seemingly unrelated regression (SUR) to compare treatment coefficients across models. For each variable, we report the mean of the comparison group, the coefficients of interest, and standard errors in parentheses. All columns include village-level fixed effects, a vector of individual characteristics, week and day-of-week fixed effects and standard errors clustered at the level of the intervention cohort. The sample in all regressions is restricted to participants in active treatment groups who attended the baseline survey. Where available, we control for the baseline outcome of the dependent variable. Outcome measures are described in Section 4. Columns (9)–(10) report the *p*-values on the differential effect of the treatments in villages with versus without chlorine dispensers using SUR.

*denotes significance at the 10%, ^**^at the 5%, and ^***^at the 1% levels.

Table 3 shows results from the main estimating equation, focusing on chlorination-related outcomes, separately for non-dispenser villages (columns (1)–(4)) and dispenser villages (columns (5)–(8)). The interaction terms on our two treatment groups with the randomized dispenser treatment are shown in columns (9) and (10). We find little evidence of heterogeneity in treatment effects across dispenser and non-dispenser villages, in either the short or long term. The Visualization treatment leads to a 5 percentage point increase in positive chlorine tests after 12 weeks in non-dispenser villages and a 4 percentage point increase in dispenser villages. Visualization increases self-reported use of chlorination and boiling to a similar extent in dispenser and non-dispenser villages. Consistent with increases in water treatment, Visualization decreases diarrhea incidents among children under 15 and under 5, with few differences across dispenser and non-dispenser villages. After 33 months, effects on self-reported chlorination persist; effects on diarrhea do not, as occurs in the average effects (potentially relating to seasonality, see Section 6.2). In the 10-week survey, Planning has smaller and mostly insignificant effects on chlorination. It leads to a small increase in boiling, and small decreases in diarrhea among children under 15, with effects (insignificantly) larger in dispenser villages. No effects persist in the 33-month follow-up.

The similarity in effects across dispenser and non-dispenser villages suggests that monetary costs may not be the binding constraint to chlorination for the households who respond to our interventions. If costs were a strong barrier, psychological interventions might have *smaller* effects in non-dispenser villages, where using chlorine is somewhat more costly. Instead, we find effects on chlorination are the same in absolute terms in non-dispenser and dispenser villages. In relative terms, they are even slightly *larger* in non-dispenser villages, as fewer households chlorinate in non-dispenser villages to begin with.

In search of further evidence, we explore where households obtained chlorine at the bottom of Table 3. Households in non-dispenser villages are still fairly easily able to access bottled chlorine, which is cheap and widely available: A total of 47% reported adding bottled chlorine to their current water, compared to 31% in dispenser villages. Households in non-dispenser villages are less likely to report using dispenser chlorine (21% compared to 54% in dispenser villages). We see suggestive evidence that increases in chlorination are driven somewhat more by increases in the use of bottled chlorine in both types of villages. This is plausible: There are more barriers to the use of bottled chlorine—although it is cheap, households must remember to buy and add it. However, we are cautious about this conclusion due to concerns about power.

## 7. Mechanisms

Our interventions targeted three psychological mechanisms, registered in our first pre-analysis plan: First, the Visualization intervention encouraged participants to connect everyday behaviors to distant future outcomes and to build their ability to make these future outcomes vivid and tangible in their minds. It thus aimed to increase patient behavior, both through valuation of the future (time preferences) and through strengthening the mapping between current behavior and future outcomes (captured in self-efficacy, or domain-general returns to effort, see footnote 3). Second, our Planning intervention aimed to build planning skills by teaching participants to structure tasks, break them down into individual steps, and approach them in a way that induces self-reinforcing motivation cycles. The intervention may also build self-efficacy by strengthening participants’ beliefs that desired outcomes are within their reach.

### 7.1. Targeted Psychological Mechanisms

Table 4 shows results on pre-specified psychological mechanisms, estimated using equation (1). All outcomes are described in Section 4.3. The raw means and standard deviations of all outcome measures that were *z*-scored are reported in Online Appendix Table G.3.

**Table 4.** Psychological outcomes.

	Endline (10–12 weeks)					Follow-up (30–36 months)					
		(1)	(2)	(3)	(4)	(5)	(6)	(7)	(8)	(9)	(10)
	MHT level	Active control group mean (SD)	Visualization treatment effect	Planning treatment effect	Column 2 versus column 3 *p*-value	*N*	Active Control group mean (SD)	Visualization treatment effect	Planning treatment effect	Column 2 versus column 3 *p*-value	*N*
*Planning skills*											
BADS score (*z*-score)	1/-	0.00	-0.03	0.05	0.21	2,103					
		(1.00)	(0.05)	(0.05)							
			[0.83]	[0.91]							
Tower of London (*z*-score)	2/-	0.00	0.02	-0.04	0.25	2,103					
		(1.00)	(0.05)	(0.05)							
			[0.49]	[0.39]							
*Time preferences*											
$\beta ^{Effort}$	1/-	0.982	0.007	0.005	0.33	2,068					
		(0.005)	(0.006)	(0.007)							
			[0.83]	[0.91]							
$\delta ^{Effort}$	2/-	0.999	-0.001	-0.002	0.16	2,068					
		(0.001)	(0.001)	(0.001)$^{**}$							
			[0.26]	[0.08]$^{*}$							
Utility forecasting: vividness (*z*-score)	-/1						0.00	0.12	-0.04	0.00$^{***}$	2,073
							(1.00)	(0.05)$^{**}$	(0.06)		
								[0.04]$^{**}$	[0.72]		
Utility forecasting: practice (*z*-score)	-/2						-0.00	0.10	0.12	0.68	2,073
							(1.00)	(0.05)$^{*}$	(0.05)$^{**}$		
								[0.17]	[0.09]$^{*}$		
$\beta ^{MPL}$	1/-	1.05	-0.01	0.02	0.27	2,103					
		(0.46)	(0.02)	(0.03)							
			[0.49]	[0.39]							
$\delta ^{MPL}$	2/-	0.98	-0.00	-0.00	0.83	2,103					
		(0.02)	(0.00)	(0.00)							
			[0.34]	[0.20]							
Time preferences, qualitative (*z*-score)	-/2						-0.00	0.09	-0.06	0.01$^{***}$	2,073
							(1.00)	(0.05)$^{*}$	(0.06)		
								[0.17]	[0.47]		
1-year discount factor (GPS staircase)	-/2						0.52	0.00	-0.01	0.21	2,073
							(0.13)	(0.01)	(0.01)		
								[0.94]	[0.47]		
*Self-efficacy*											
GSE (*z*-score)	2/1	0.00	0.15	0.11	0.45	2,103	-0.00	0.07	-0.07	0.01$^{***}$	2,073
		(1.00)	(0.05)$^{***}$	(0.05)$^{**}$			(1.00)	(0.05)	(0.05)		
			[0.01]$^{***}$	[0.08]$^{*}$				[0.09]$^{*}$	[0.72]		

Notes: OLS estimates of treatment effects. For each variable, columns (1) and (6) report the mean and standard deviation of the control group. Columns (2) and (3) and columns (7) and (8) report the coefficients of interest and standard errors in parentheses, respectively. Square brackets contain *p*-values corrected for MHT using the FDR. The column ``MHT level'' indicates the MHT level: whether variables were pre-specified as primary (1), secondary (2), or exploratory outcomes (3), or were not pre-specified (np). The first number relates to pre-specification in the 10-week survey; the second relates to the 30–36-month survey. All regressions include village-level fixed effects, controls for individual characteristics, week and day-of-week fixed effects and standard errors clustered at the level of the intervention cohort. The sample in all regressions is restricted to participants in active treatment groups who attended the baseline survey. Where available, we control for the baseline outcome of the dependent variable. Outcome measures are described in Section 4.

*denotes significance at the 10%, ^**^at the 5%, and ^***^at the 1% levels.

#### Planning Skills.

In the first panel of Table 4, we find no statistically significant effects of any treatment on planning skills, measured by the BADS and ToL, compared to the active control group after 10 weeks. However, the coefficients in the Planning treatment have the expected signs (positive for BADS and negative for the number of moves required to complete the ToL task).

#### Time Preferences and Utility Forecasting.

The second panel shows that the Visualization intervention has few effects on conventional measures of time preferences but appears to improve utility forecasting. Our primary outcome in the 10-week follow-up is $\beta ^{\text {Effort}}$, which captures present bias in our effort task. (See Section 4.3 and Online Appendix G.2 for details of the structural estimation.) Secondary outcomes are the $\delta ^{\text {Effort}}$ parameter from the same task and corresponding parameters from the monetary discounting task. We find no statistically significant effects of the Visualization treatment on the estimated preference parameters.^39^

In the long-run survey, we measure outcomes more fine-tuned to the Gabaix-Laibson model of intertemporal choice (Section 3.1), namely participants’ ability to forecast future utility. Our primary pre-specified psychological outcome for this survey round captures how vivid participants’ images of themselves and their family are in one year’s time, and is our proxy for forecasting noise $\sigma _{\epsilon _{t}}^{2}$ in the Gabaix-Laibson model. We find that participants in the Visualization group report significantly more clear and vivid utility simulations after almost 3 years. There is no effect in the Planning group. A secondary outcome asks participants whether they typically mentally imagine the consequences of decisions when making everyday choices (the “practice” of utility forecasting). This measure relates to both the Gabaix-Laibson model (regular practice of forecasting may improve forecasting ability and reduce forecasting noise), as well as to the concept of self-efficacy (the belief that one’s behavior affects future outcomes). We find significant increases in both Visualization and Planning treatments. A consistent interpretation is that effects on behavior are driven by forecasting *ability*, and that the regular *practice* of utility forecasting by itself (without an associated increase in forecasting skill) is not sufficient to generate behavioral change.

Given our utility forecasting results, it may seem puzzling that we do not observe an effect on conventional measures of time preferences: An increased weight of the future relative to the present should be captured in our estimates of $\beta ^{\text {Effort}}$. However, there are important differences between the Gabaix-Laibson model and conventional models of quasi-hyperbolic discounting (“$\beta \delta$-models”): While both models can generate hyperbolic discounting patterns, $\beta \delta$-models assume the existence of fundamental and stable parameters ($\beta$ and $\delta$). In contrast, the Gabaix-Laibson model stipulates that patience is inherently unstable, as the forecasting noise $\sigma _{\epsilon _{t}}^{2}$ can vary with cognitive load, time spent thinking about a problem, and how cognitively well-simulated the relevant future period is. Thus, details of the choice frame and setting matter.

Importantly, the intervention prompted participants to visualize alternative realizations of their future in one year—a time horizon where future utility is likely to feel vague and distant. In contrast, intuitively speaking, time preferences are estimated from participants’ relative willingness to exert effort in 0 versus 1 days (for $\beta \delta )$ and 7 versus 8 days (for $\delta$, see Section 4.3). It is plausible that the intervention made far-future utility from abstract rewards (e.g. having healthy children in one year) more tangible and salient, while the disutility from near-future effort (SMS data entry within the next 8 days) was already tangible at baseline. This could explain the observed changes in chlorination and savings behavior without affecting our lab measures of $\beta$ and $\delta$.

We investigate the importance of the time horizon further in the long-run follow-up by including a hypothetical monetary discounting task (Falk et al. 2018), which estimates the discount factor between today and 12 months from now. We find no effects on this measure, but note that the informational value is compromised by the fact that only 34% of respondents had 1-year discount factors in the measurable range $[0.46,\, 1]$,^40^ as well as by standard concerns about hypothetical and monetary discounting measures (Andreoni and Sprenger 2012). However, we do find that the Visualization intervention leads to a 0.09 SD increase in the GPS qualitative time preference measure (reported willingness to give up current benefits to obtain future rewards). The effect is significantly larger than in the Planning group ($p<0.01$). We conclude there is limited evidence of effects on conventional time preference measures.

#### Self-Efficacy.

The third panel of Table 4 shows the effect of our interventions on self-efficacy. After 10 weeks, both the Visualization and the Planning interventions generate statistically significant 0.15 SD and 0.11 SD increases, respectively. The effect in the Visualization group is 36% larger than that in the Planning group, though the difference is not statistically significant. We find evidence that the effect of Visualization on self-efficacy persists in the long run: After almost 3 years, this group has 0.14 SD higher self-efficacy than the Planning group ($p<0.01$) and 0.07 SD higher self-efficacy than the active control ($p=0.10$). The result is robust to MHT correction.^41^

Finally, we present simple predictive regressions of key chlorine and savings outcomes on our psychological measures in Online Appendix Tables D.2 and D.3. These do not have a causal interpretation, as many of the regressors are endogenous to treatment. We find that our chlorination measures are robustly and significantly linked with self-efficacy across all survey rounds. There is a positive but insignificant association with utility forecasting. In contrast, savings measures are strongly predicted by both utility forecasting and self-efficacy. For both chlorination and savings, other psychological mechanisms have little predictive power. We conclude that both self-efficacy and utility forecasting are likely drivers of our treatment effects, with self-efficacy playing a larger role for chlorination, and utility forecasting playing a relatively larger role for savings.

In short, we find plausible evidence of which psychological mechanisms—utility forecasting and self-efficacy—are activated by our treatments, in particular by the Visualization treatment. These mechanisms are correlated with chlorination and savings in the absence of treatment. There is persistence in effects, despite the light-touch nature of the interventions.

### 7.2. Beliefs, Knowledge, and Risk Preferences

We tested a range of alternative psychological channels, with results shown in Table 5. The first panel shows results on beliefs and knowledge about chlorination. Beliefs in the efficacy of chlorine in averting diarrhea, as well as knowledge about how to correctly chlorinate water, are similar in the Visualization, Planning and active control groups. This is consistent with the fact that all active arms received the information module. Importantly, beliefs and knowledge do increase in all active arms, relative to the pure control group (see Online Appendix Table E.3). Thus, the information module was somewhat effective. Correlations of beliefs and knowledge with TCR after 12 weeks are shown in Online Appendix Table D.4.

**Table 5.** Alternative mechanisms.

	Endline (10–12 weeks)					Follow-up (30–36 months)				
	(1)	(2)	(3)	(4)	(5)	(6)	(7)	(8)	(9)	(10)
	Active control group mean (SD)	Visualization treatment effect	Planning treatment effect	Column 2 versus column 3 *p*-value	*N*	Active control group mean (SD)	Visualization treatment effect	Planning treatment effect	Column 2 versus column 3 *p*-value	*N*
*Beliefs, knowledge and risk preferences*										
Belief: diarrhea avoided through chlorination (*z*-score)	0.00	0.08	0.05	0.56	2,103					
	(1.00)	(0.05)	(0.05)							
Chlorine knowledge score (*z*-score)	0.00	0.07	0.00	0.22	2,103					
	(1.00)	(0.05)	(0.05)							
ANC/PNC knowledge score (*z*-score)	0.00	0.04	-0.05	0.09$^{*}$	2,103					
	(1.00)	(0.05)	(0.05)							
Risk aversion measure (*z*-score)	0.00	-0.04	-0.08	0.52	1,926					
	(1.00)	(0.06)	(0.06)							
*Salience task*										
Chlorine word remembered	0.40	0.06	0.03	0.095$^{*}$	6,336	0.42	0.10	0.04	0.028$^{**}$	2,073
	(0.49)	(0.01)$^{***}$	(0.01)$^{**}$			(0.49)	(0.03)$^{**}$	(0.03)		
Savings word remembered	0.45	-0.02	0.00	0.124	6,336	0.45	0.04	-0.01	0.069$^{*}$	2,073
	(0.50)	(0.01)	(0.01)			(0.50)	(0.02)	(0.02)		
Total words remembered	4.23	-0.09	0.04	0.105	6,336	4.45	0.28	0.20	0.336	2,073
	(1.64)	(0.07)	(0.07)			(1.61)	(0.09)$^{**}$	(0.09)$^{*}$		

Notes: OLS estimates of treatment effects. For each variable, columns (1) and (6) report the mean and standard deviation of the control group. Columns (2) and (3) and columns (7) and (8) report the coefficients of interest and standard errors in parentheses, respectively. Square brackets contain p-values corrected for MHT using the FDR. The column ``MHT level'' indicates the MHT level: whether variables were pre-specified as primary (1), secondary (2), or exploratory outcomes (3), or were not pre-specified (np). The first number relates to pre-specification in the 10-week survey; the second relates to the 30–36-month survey. All regressions include village-level fixed effects, controls for individual characteristics, week and day-of-week fixed effects and standard errors clustered at the level of the intervention cohort. The sample in all regressions is restricted to participants in active treatment groups who attended the baseline survey. Where available, we control for the baseline outcome of the dependent variable. Outcome measures are described in Section 4. The bottom panel of the table reports the probability of remembering a chlorine-related word, or a savings-related word, on a given word list in the salience task. In the endline survey, participants were read three word lists, resulting in three observations per individual. In the long-run follow-up, participants were read one word list (randomly selected out of three lists). Salience regressions additionally control for the total number of words the participant remembered on that word list.

*denotes significance at the 10%, ^**^at the 5%, and ^***^at the 1% levels.

We see effects on beliefs and knowledge in all three active arms, but only Visualization has statistically significant effects on chlorination and diarrhea. Thus, the Visualization treatment has additional effects on behavior compared to the effect of information on its own. These are likely due to changes in psychological outcomes discussed above. Finally, we find no effects on risk aversion, suggesting that any behavioral effects are unlikely to result from changes in risk preferences induced by our treatments.

### 7.3. Salience

Section 4.4 explains the design of a test for increased salience of chlorination, and Online Appendix G explains the econometric specification. The bottom panel of Table 5 shows that, in the 10-week endline, participants who had received the Visualization or Planning intervention found it easier to remember chlorine-related words, *conditional* on the total number of words remembered. In the long-run survey, this effect persists only for Visualization.^42^

There are three possibilities for the role of salience in our study. First, the interventions may cause participants to pay more attention to chlorination and thus chlorinate more (i.e. salience is one mechanism which accounts for our treatment effects). Although they were domain-general, Visualization and Planning interventions use water treatment as an example behavior in engaging stories or exercises. Second, participants may chlorinate more, and this makes chlorination more salient to them (i.e. reverse causality). Third, the interventions may change the salience of chlorination, but this may not relate to behavior change (i.e. those for whom salience responds are not those who chlorinate).

We find evidence consistent with the second and third explanations, but little evidence consistent with the first explanation. Consistent with the second explanation, effects on both chlorination and the salience of chlorination persist only in Visualization. However, any chlorine references in the Visualization and Planning group were identical. Furthermore, salience effects in Visualization appear to *increase* over time, suggesting they are not a direct response to the intervention. Consistent with the third explanation, there is no correlation between the salience of chlorination and chlorination behavior (Online Appendix Table D.4). Moreover, we observe changes in behavior without a change in salience: Despite persistent effects on savings measures, the salience of savings is unaffected. We tentatively conclude that salience is less likely as a mechanism than other psychological mechanisms we have identified.

## 8. Conclusion

Behavioral constraints may explain low demand for preventive health products, as well as other behaviors requiring participants to incur current costs to secure future benefits. Potential constraints may include people’s ability to visualize future benefits of investments, or their ability to make concrete plans. We study whether two light-touch interventions, one targeting each of these constraints, affect behavioral and psychological outcomes among young women in Kenya. We conduct an in-person survey and incentivized choice tasks after 10 weeks, and test drinking water for the presence of chlorine in unannounced household visits after 12 weeks; we follow up by phone after 30–36 months.

The Visualization intervention is more effective than the Planning intervention in the short and particularly in the longer term. The Visualization intervention increases chlorination in a water test after 12 weeks and self-reported chlorination after both 10 weeks and 30–36 months, relative to an active control group. We observe reductions in child diarrhea in the short but not in the long term. Visualization also increases savings in the short and long term and labor supply in the long term. The Planning intervention, in contrast, has few significant effects. The Visualization intervention is highly cost-effective relative to WHO benchmarks. Possible mechanisms for behavioral effects include self-efficacy (participants’ belief that their current behavior affects future outcomes) and utility forecasting (participants’ ability to mentally simulate future utility (Gabaix and Laibson 2017)).

Our results suggest that visualization-based interventions might be effective in increasing take-up of other preventive health products and be a valuable focus of government policy. Our modules were administered by field workers with no specific training. It would thus be straightforward to incorporate them into the curricula that community health workers (CHWs) administer during household visits or community education sessions to promote preventive health products. Instead of domain-general content like ours, practitioners may choose to further focus the exercises on specific target behaviors. The regularity of the work of CHWs might also overcome the fact that our effects on diarrhea fade out after 3 years—effects may persist if the intervention is refreshed periodically. In the context of recent work raising concerns about the efficacy of CHW programs in improving chlorination take-up (Dupas et al. 2020), our results suggest that improving the content of preventive health promotion interventions might enhance their effectiveness at a low cost.

The observed effects on savings outcomes and labor supply suggest that visualization-based interventions might be used in other domains where policy-makers wish to encourage forward-looking behavior. Such interventions could form part of the training for members of ROSCAs or Village Savings and Loan Associations (VSLAs). They could also be used to enhance repayment discipline among borrowers of microfinance institutions. Furthermore, in ongoing work by Ashraf et al. (2020), visualization is incorporated in an intensive business skills training for entrepreneurs in Colombia.

The experiment suggests a number of areas for future research. A key one is to better understand and measure the mechanisms behind visualization-based interventions. Task-based measures of utility forecasting ability might be particularly valuable. Conducting water testing in longer-term follow-ups, outside a pandemic context, may further increase the credibility and external validity of the intervention. A second potential area of research is to examine if “topping-up” the intervention with repeat sessions ensures that effects on health behaviors persist. In other trials, reminders improved adherence to existing health programs (Pop-Eleches et al. 2011). A third potential area of future research may be to explore whether domain-general planning interventions can be better adapted to target populations or whether planning skills do not constitute a binding constraint to future-oriented behavior.

## Supplementary Material

jvab052_John_Orkin_Online_Appendix

jvab052_John_Orkin_Reproduction

jvab052_John_Orkin_TeachingMaterial

## Footnotes

1. For instance, insecticide-treated bednets reduce the incidence of malaria, which increases farmworker productivity (Dillon, Friedman, and Serneels 2020), farm yields (Fink and Masiye 2015), and children’s earnings in adulthood (Bleakley 2010). Similarly, treating intestinal parasites improves child health and school attendance (Miguel and Kremer 2004), test scores (Ozier 2018), and adult wages (Baird et al. 2016). Yet demand for bednets and deworming pills is low and price-sensitive (Cohen and Dupas 2010; Dupas 2014; Kremer and Miguel 2007).

2. We conservatively assume that treatment effects drop to zero immediately after three months. If, instead, effects persisted for one year, the cost per DALY would be reduced to USD 62 per child under 5. We also do not account for benefits for children older than 5. Extrapolation to children under 15 yields a cost per DALY of USD 108 for the 3-month effects. See Online Appendix B for detailed calculations.

3. Self-efficacy is a psychological concept, which captures beliefs about one’s ability to achieve desirable outcomes and overcome obstacles (Bandura 1977; Reich and Infurna 2016). We hypothesized that both interventions might affect self-efficacy, as both required a strong mapping between current behavior and future outcomes. Specifically, visualizing alternative realizations of the future depending on current behavior requires the belief that current behavior can *affect* future outcomes. Similarly, making plans both requires and enforces the belief that desired outcomes are within an individual’s reach. Self-efficacy is closely related to the economic concept of believed returns to effort.

4. We discuss possible reasons for the divergence of our time preference measures in Section 7.

5. See McKelway (2018) for the effect of self-efficacy on women’s labor supply.

6. Future research is needed to confirm and better understand this mechanism, such as the ongoing work on visualization-based business skills training by Ashraf et al. (2020).

7. One ongoing study examines the effects of a 4-week BA treatment targeted at depressed people on labor market outcomes four years later (de Quidt et al. 2020).

8. Given the size of the literature, our citations here focus on interventions pertaining to water quality and chlorination. For comprehensive reviews of the preventive health literature, see Dupas and Miguel (2017) and Kremer and Glennerster (2011).

9.
https://www.who.int/news-room/fact-sheets/detail/drinking-water.

10. For more information on the WASH study, see Online Appendix C.

11. The wealth index consisted of the total value of a limited set of assets (bicycles, cellphones, gas stoves, all livestock, radios, sofas, and televisions).

12. At the time of the study, USD 1 was equivalent to approximately KES 100.

13. Participants were informed of the participation fees during the phone call inviting them to a session and were told that they would have the opportunity to earn some extra money during the session.

14. A meta-analysis finds point-of-use water chlorination methods reduced relative risk of any episode of child diarrhea in the last two weeks by 34%, across 9 studies (Arnold and Colford 2007).

15. In the 10-week survey, participants are asked, “If three children are sick with diarrhea, how many of these cases could have been prevented with chlorination?” A total of 61% say chlorine would prevent diarrhea in all three children; only 20% say it would not prevent diarrhea in any children. The true number is slightly less than one case.

16. See Section 4.1 on the distinction between free and total chlorine. We report free chlorine here for comparison with other studies, but focus on total chlorine as our primary outcome measure.

17. Unchlorinated water mean score $=6.21/10$, chlorinated water mean score $=6.06/10$, $p=0.66$, $n=90$.

18. Behaviors participants wanted to change included avoiding impulse purchases, saving, searching more for work, taking a loan to start a business, treating water, purchasing a water tank to get cleaner water, eating better, or encouraging children to wash their hands after going to the bathroom.

19. One way to microfound this is to think of forecasting effort as the number of simulations $s_{t}$ that are generated. With *n* simulations, forecasting variance decreases by ${{1}/{n}}$.

20. It is possible to obtain a similar prediction using conventional models of present-biased preferences (Laibson 1997 and O’Donoghue and Rabin 1999), which place a differential weight ${{1}/{\beta }}$ on the present. Using these models, the key question for our intervention is whether the fundamental preference parameters $\beta$ and $\delta$ can be changed persistently by increasing the focus on the future (see also Becker and Mulligan 1997). See Section 7.1 for more discussion on similarities and differences between the models’ predictions.

21. Murphy et al. (2016) tested water samples that were properly chlorinated and kept in normal conditions in rural Kenyan homes. A total of 17% of samples kept in jerry cans had no measured TCR after 48 hours. In clay pots, used by most of our sample, TCR had disappeared in 51% of samples after 48 hours.

22. We validated the Tower of London task, Monetary Price Lists for time preferences, and the self-efficacy scale in Kenya, testing internal consistency, test-retest reliability, and construct validity (Esopo et al. 2018).

23. All participants have access to a mobile phone: 71% own one, 96% have one in their household, and the remainder shares the phone of friends or relatives.

24. In line with the Gabaix and Laibson (2017) prediction that forecasting ability increases with intelligence, this measure correlates with participants’ education level ($t=3.16)$.

25. We similarly check for differential knowledge of WHO-recommended ANC and PNC. These questions were included to pilot them for a future study.

26. Week and day of week fixed effects were not pre-specified, but were added to account for a slight imbalance in survey delay (see Section 6.1). We exclude these in a robustness check.

27. Where only some baseline observations are missing, we replace the missing values with the sample mean at baseline and add a dummy variable indicating such cases.

28. We see some increases over time in self-reported water treatment. In the short-run (long-run) follow-up, in the active control, 73 (85) percent report they generally chlorinate, 35 (63) percent generally boil water, and 27 (54) percent report using both methods. Only 20 (6) percent report no or only ineffective methods, which suggests that effective water treatment is relatively widespread in our study area, though it may not be used consistently. Time trends in water treatment may be due to changing social norms, or increased public health awareness during the COVID-19 pandemic, including public health campaigns. However, we cannot disentangle time effects in underlying behavior from the effect of adapting questions to a phone survey format (see Section 4.1). Estimates of treatment effects as a percentage of the mean can be compared between rounds.

29. We pre-specified diarrhea in children under 15 (as opposed to younger age groups) to maximize sample size, and because children in a wide age range suffer negative health effects from ongoing enteric dysfunction.

30. In the two rainy seasons (February-May and November-December), one main cause of diarrhea is rain washing faeces into water sources, contaminating water. In the dry season (June to September), rotavirus, a major cause of diarrhea, becomes more prevalent. It is transmitted through air and human contact. See Luby et al. (2006) and Arnold and Colford (2007) on the importance of seasonality in interpreting the effects of chlorination interventions on diarrhea.

31. We elicited 13 types of water sources following WHO guidelines in the 33-month survey, so this is potentially endogenous. We classify as unprotected: surface water, rainwater, unprotected springs and wells.

32. Across six randomized controlled trials which measure both outcomes, a 1% increase in detectable free chlorine is correlated with a 1% reduction in child diarrhea incidence in the past two weeks, with confidence intervals from a 2.3% decrease to a 0.5% increase.

33. Respondents are assigned to a group A (B), and told “We hypothesize that people who participated in this study and received the same treatment as you will give higher (lower) responses to these questions than others.” They are then asked how often they added chlorine to water collected from their primary source in the last 7 days. Following de Quidt, Haushofer, and Roth (2018), the responses can be used to obtain bounds $a^{+}(\zeta )$ and $a^{-}(\zeta )$ for the impact of experimenter demand effects on self-reports. We do not reject the equality $a^{-}(\zeta )=a^{+}(\zeta )$, neither for the overall sample ($p=0.92$) nor within any of the treatment groups (Online Appendix Table H.1).

34. We made pre-specified adjustments to how the savings variable is elicited between rounds, which likely explains the sizable increase in means (KES 94 in the 10-week endline versus KES 408 in the long-run follow-up). The 10-week survey asked respondents how often they put money aside for a given savings *purpose* and how much each time. The variable “Amount saved regularly (per week, KES) is obtained by converting to weekly frequency and summing across savings purposes. In the long-run survey, we asked how often they put money aside in a given savings *place* (safe hiding place, bank account, ROSCA...) and how much each time. This will yield a more comprehensive measure if people also saved for unspecified purposes. We suspect that participants included ROSCA savings when asked by place in the long-run survey, but not when asked by purpose after 10 weeks: Given the inflexibility of ROSCA schedules to individual needs, ROSCA savings are more likely to be for unspecified purposes. We also report ROSCA savings separately in both rounds. If our interpretation is correct, total weekly savings are KES 300 in the short run, and KES 408 in the long run.

35. As before, the difference in means is likely explained by a pre-specified change in the elicitation method.

36. Savings behavior has changed since the pandemic. About 11% of respondents report that they reduced their regular savings since the start of public health restrictions in Kenya (March 2020), with an average conditional reduction of 73% in contributions. Treatment effects in the absence of the pandemic may have been different.

37. We verify the WASH randomization remains intact. A total of 70% of active control participants in dispenser villages in the 33-month survey report they have access to a working dispenser at their primary or secondary water source, and 82% have a dispenser within 30 minutes walking distance. This proportion is lower, 34% and 49%, respectively, in non-dispenser villages. These figures suggest that some non-dispenser villagers can still access dispensers, potentially because they live at the edge of dispenser villages.

38.
Online Appendix Table F.4 shows that participants in dispenser and non-dispenser villages were comparable on baseline characteristics, although in dispenser villages they had a slightly higher level of education.

39. Somewhat surprisingly, we find an (economically small -0.002) reduction of the $\delta ^{\text {Effort}}$ parameter in the Planning group, driven by an increased willingness to supply effort 7 and 8 days in the future, at constant willingness to supply effort today or tomorrow (see Online Appendix Figure G.3). This result is consistent with the Planning intervention helping participants to plan the logistics of future effort provision (SMS data entry), such as phone access or child care (which is possible for 7 or 8 days, but perhaps too short notice for 0 or 1 days).

40. We use the official stakes for Kenya from the GPS (Falk et al. 2018).

41. The sharpened *q*-values are lower than unadjusted *p*-values. This can occur when many hypotheses in the outcome group are rejected (Anderson 2008).

42. The interventions are unlikely to have affected participants’ memory in general, proxied by the total number of words remembered. There is a small increase of 0.2–0.3 words remembered in the long-run survey, but this likely reflects that participants remembered chlorine words *in addition* rather than *instead* of filler words. All salience regressions condition on the total number of words remembered.

## Notes

*The editor in charge of this paper was Imran Rasul*.
