# Supplementary material for: Can Simple Psychological Interventions Increase Preventive Health Investment?
Source: J Eur Econ Assoc. 2021 Nov 30;20(3):1001–47. doi: 10.1093/jeea/jvab052 (PMC9194950; doi:10.1093/jeea/jvab052)
Supplement: jvab052_John_Orkin_Online_Appendix [file jvab052_john_orkin_online_appendix.pdf]

ONLINE APPENDIX: CAN SIMPLE PSYCHOLOGICAL INTERVENTIONS INCREASE PREVENTIVE HEALTH INVESTMENT?

Anett John
University of Birmingham

Kate Orkin
University of Oxford

Appendix A: Overview of Interventions

	VISUALIZATION	PLANNING	ACTIVE CONTROL
**Target problem**	Impatient behavior caused by inability to imagine the future	Inactivity cycles caused by avoidance and negative mood	
**Key conceptual reference**	Gabaix and Laibson (2017)	Lejuez et al (2011)	
**Key Content**	1. Connect present behavior to future outcomes<br>2. Visualize alternative realizations of the future depending on current behavior<br>3. Put yourself in the shoes of your future selves, imagine how they feel, and 'talk' to them.	1. Write lists of necessary, routine, and pleasurable tasks<br>2. Rate tasks from most to least difficult<br>3. Schedule in diary, starting systematically with easiest tasks<br>4. Break tasks into steps, anticipate obstacles	Placebo session: Lectures, exercises and drawings on birds and plants of Kenya
**Psychological Targets**	Time Preferences<br>Self-Efficacy	Planning Skills<br>Self-Efficacy	Not psychologically active

Sessions for all treatments included interactive lectures, case stories, exercises and drawings

FIGURE A.1. Overview of the interventions.

E-mail: A.John@bham.ac.uk (John); kate.orkin@bsg.ox.ac.uk (Orkin)

Journal of the European Economic Association
Preprint prepared on 25 March 2022 using jeea.cls *v1.0.*

Appendix B: Cost-Effectiveness Per Disability-Adjusted Life Year Saved

To compute the cost per disability-adjusted life years saved with our interventions, we proceed as follows. The cost of our interventions was USD 4 per household. This is USD 3.70 per child under 5 or USD 1.58 per child under 15 because participants have 1.08 children under 5 and 2.53 children under 15 on average. This includes the costs relevant to a potential scale-up, i.e. the cost of running the sessions including overheads, but excludes our incentivized surveys, as well as the cost of sampling/targeting 18-35 year old women. Troeger et al. (2018) estimate the DALYs lost per child under 5 in Kenya due to diarrhea in 2016, the year before our study was conducted, to be 0.127. This includes both acute, immediate effects of illness and/or death and the longer-term burden of disease associated with growth impairment due to diarrhea. Our region is one of the poorer ones in Kenya and likely has worse health outcomes and a higher burden of disease from diarrhea than the Kenya-wide average, so using this figure will lead to a conservative estimate.

We focus on children under 5, as these are the focus of the estimates in Troeger et al. (2018). We assume that the treatment effects of our interventions on diarrhea relative to the active control group were never higher during the study period of three months than what we measured at the 3-month endline, and then immediately went to zero. This, too, is a conservative assumption and will lead to a lower-bound estimate. For children under 5, Table 2 shows reductions in under-5 diarrhea of 47% in the Visualization group, and no significant effect in the Planning group. DALYs saved due to the Visualization intervention are $0.127 \times 47\% \times 3/12 = 0.0149225$, if one accounts for both acute and long term effects of diarrhea. The cost of one DALY saved is USD $3.70/0.0149225 =$ USD 248.

For children under 15, the effect of our interventions on diarrhea relative to the active control group is 46% for Visualization and 23% for Planning. We could not find published estimates for Kenya of DALYs lost per child under 15 annually to diarrhea, so we use the same figure of 0.127 as for under 5 children. This likely overstates the disease burden on children under 15, as older children are less likely to die. However, older children do benefit from a reduction in ongoing enteric dysfunction, which may cause adverse effects like stunting and impaired cognition in a wide age range. Thus, a restriction to children under 5 would be too narrow. With this assumption, DALYs saved per child due to the Visualization intervention are 0.014605, and of the Planning intervention 0.0073025. Combined with an intervention cost of USD 1.58 per child for both interventions, this implies that the cost of one DALY saved is USD $1.58/0.014605 =$ USD 108 for the Visualization intervention and USD $1.58/0.0073025 =$ USD 216 for the Planning intervention.

Extrapolating to other contexts, these numbers will be sensitive to the number of children per treated participant. Estimates are also sensitive to how long the effect on diarrhea lasts: If, instead of three months, the effects lasted for one year and then faded out, the estimated cost per DALY saved for the Visualization intervention would be reduced to USD 27 (when considering children under 15) and USD 62 (when considering only children under 5). All our estimates remain highly cost-effective by WHO standards. The WHO classifies an intervention as “cost-effective” for a cost

per DALY saved below USD 4525, and “highly cost-effective” below USD 1508 (https://www.who.int/bulletin/volumes/93/2/14-138206/en/).

Policymakers may be interested in the comparison of our interventions to other cost-effective preventive health interventions, such as insecticide-treated nets. Cost effectiveness estimates for malaria bednets range from USD 29 to 100/DALY (Wisniewski et al. 2020), depending on assumptions. The WHO estimates a cost per DALY of USD 29 to 34 (https://www.who.int/news-room/feature-stories/detail/new-cost-effectiveness-updates-from-who-choice). The higher estimate of USD 100 is from GiveWell (https://forum.effectivealtruism.org/posts/HbunzTyFPRwcYihg6/long-lasting-insecticide-treated-nets-usd3-340-per-life). A recent meta-analysis of studies in Africa estimates a cost per DALY between USD 42 and USD 80 (Wisniewski et al. 2020).

Appendix C: Description of WASH Benefits Kenya

The WASH Benefits Kenya study is a cluster-randomized controlled trial testing the effects of six "water, sanitation and handwashing" (WASH) interventions on childhood development (Null et al. 2018). Villages were eligible if they were rural, most of the population relied on communal water sources and had unimproved sanitation facilities, and there were no other ongoing water, sanitation, handwashing, or nutrition programs. Between 2012 and 2014, 8,246 pregnant women were enrolled from three counties in western Kenya: Bungoma, Kakamega and Vihiga. The six interventions were (W) improved water quality ("Water Quality"); (S) improved sanitation; (H) handwashing with soap; (WSH) combined water, sanitation and handwashing; (N) improved nutrition; (WSH+N) combined water, sanitation, handwashing and nutrition. The study also included two control arms: (A) an active control arm, who received monthly visits to measure children's arm circumferences; and (P) a passive comparison arm, who received no visits. The villages in our sample primarily came from either (W) improved water quality or (P) passive comparison arm.

In all villages in arm W, "Water Quality," chlorine dispensers were installed at public water sources used by study participants. All community members were able to use the dispensers free of charge. After filling water into carrying containers, usually a 20 liter jerrycan, users turn the knob on the dispenser to add 3 ml of 1.25% sodium hypochlorite (chlorine), which yields 2.5 ml/l of free chlorine residual after 30 minutes for 20 liters of water(Kremer et al. 2011). This is sufficient chlorine to sanitize a jerrycan of water for drinking. Community promoters encouraged use of the chlorine dispensers, which to this date are monitored and maintained by *Evidence Action's* "Dispensers for Safe Water" program. WASH participant households (excluded in our study) additionally received a six-month supply of bottled chlorine every six months, to be used for sanitizing water at home, in case the household drank harvested rainwater or chose not to use the water source with the installed dispenser.

We compare villages which received the dispenser intervention to villages which do not. Our "dispenser villages" include 67 villages from the "Water Quality" (henceforth "dispenser") treatment arm. We also include 23 villages which received dispensers, as well as combinations of sanitation, handwashing or nutrition interventions at household level. Our "non-dispenser villages" include 67 villages from the "Passive Comparison" arm, which received no interventions. We also include 48 villages who received one of sanitation, handwashing or nutrition interventions, but no dispensers. These additional interventions all took place at the household level, and we do not sample households which participated directly in the original WASH study. Even in WASH households,Null et al. (2018) find no effect of these interventions on whether stored drinking water has detectable free chlorine. In addition, all interventions other than dispensers finished three to four years before our study. We had intended to select only villages from the Water Quality and Passive Comparison arms. The 23 villages with dispensers and other WASH interventions, and the 48 villages with no dispensers and WASH interventions were selected due to a coding error in the sampling in Mumias district, Kakamega county.

Appendix D: Supplementary Evidence on Mechanisms

TABLE D.1. Treatment effect heterogeneity on diarrhea by season and water source.

	Any diarrhea in u15 children, last 7 days (33 months)		
	(1)	(2)	(3)
Visualization	-0.0148	-0.0150	-0.0156
	(0.0162)	(0.0144)	(0.0165)
Planning	-0.0125	-0.00896	-0.0129
	(0.0170)	(0.0147)	(0.0172)
Unprotected water source (33m)	0.00994		-0.00912
	(0.0259)		(0.0266)
Surveyed during rainy season (33m)		0.0500	-0.102***
		(0.0778)	(0.0390)
Surveyed during rainy season× Unprotected water source (33m)			0.359**
			(0.163)
V× Unprotected water source (33m)	-0.0143		0.00237
	(0.0338)		(0.0350)
P× Unprotected water source (33m)	0.00988		0.0175
	(0.0343)		(0.0348)
V× Surveyed during rainy season (33m)		-0.0842	0.0731
		(0.0824)	(0.0499)
P× Unprotected water source (33m)		-0.0387	0.0583
		(0.100)	(0.0741)
V× Surveyed during rainy season× Unprotected water source (33m)			-0.399**
			(0.173)
P× Surveyed during rainy season× Unprotected water source (33m)			-0.0702
			(0.246)
Observations	2045	2045	2045
Control Mean	0.07	0.07	0.07

Notes: The table reports OLS estimates of heterogeneous treatment effects on diarrhea in the 33-month survey, by season and type of water source. The sample is restricted to individuals who attended the baseline survey. *Surveyed during rainy season* is an indicator for whether the respondent was surveyed after November 1, 2020 for the follow-up survey. *Unprotected water source* equals 1 if the household's primary water source is not protected (unprotected well, unprotected spring, rainwater, surface water), and 0 otherwise (private or public tap, borehole or tubewell, protected well or spring). We report standard errors in parentheses. All columns include village-level fixed effects, control for diarrhea at baseline and a vector of individual characteristics, and cluster standard errors at the level of the intervention cohort. As regressors are potentially endogenous to treatment, all regressions should be interpreted as correlational evidence. * denotes significance at 10 pct., ** at 5 pct., and *** at 1 pct. level.

TABLE D.2. Correlates of chlorination.

	Main Treatment: Chlorine (baseline)	Chlorine present in water (TCR) (12 weeks)		Main Treatment: Chlorine (10 weeks)		Main Treatment: Chlorine (33 months)	
	(1)	(2)	(3)	(4)	(5)	(6)	(7)
Visualization		0.0426*	0.0593***	0.0752***	0.0852***	0.0577***	−0.00542
		(0.0221)	(0.0214)	(0.0223)	(0.0254)	(0.0191)	(0.0180)
Planning		0.0154	0.0318	−0.0252	0.0285	0.0304	−0.0268
		(0.0257)	(0.0216)	(0.0255)	(0.0265)	(0.0199)	(0.0180)
Active Control			0.0230		0.0380		−0.0479**
			(0.0213)		(0.0255)		(0.0189)
Tower of London (baseline)	−0.00172	−0.0000		−0.00411***			
	(0.00164)	(0.00141)		(0.00159)			
β^{Effort} (baseline)	−0.00598	0.00272		0.00355			
	(0.00467)	(0.00364)		(0.00520)			
δ^{Effort} (baseline)	−0.00797	−0.00794		0.0104			
	(0.0138)	(0.00847)		(0.0126)			
General Self-Efficacy Score (GSE) (z-score) (baseline)	0.0212**	0.000179		0.0400***			
	(0.0104)	(0.00943)		(0.00965)			
Tower of London (10w)			−0.000982		-0.00595***	−0.00117	−0.000768
			(0.00114)		(0.00132)	(0.00125)	(0.000966)
β^{Effort} (10w)			−0.00106		-0.00340	−0.00797	−0.00112
			(0.00132)		(0.00210)	(0.00909)	(0.00123)
δ^{Effort} (10w)			−0.0196*		0.00855	−0.0210	−0.0106
			(0.0101)		(0.0142)	(0.0163)	(0.0109)
General Self-Efficacy Score (GSE) (z-score) (10w)			0.0108*		0.0268***	0.0110*	0.00778
			(0.00627)		(0.00711)	(0.00644)	(0.00555)
Utility Forecasting: Vividness (z-score) (33m)						0.00463	0.00311
						(0.00858)	(0.00689)
Utility Forecasting: Practice (z-score) (33m)						0.00850	0.0132*
						(0.00940)	(0.00779)
Time Preferences, qualitative (z-score) (33m)						−0.00979	−0.00373
						(0.00888)	(0.00710)
One-year discount factor (GPS staircase) (33m)						0.0984	0.0859
						(0.0601)	(0.0529)
Main Treatment: Chlorine(baseline)				0.213***		0.0816***	
				(0.0235)		(0.0183)	
High wealth index (census)	−0.00431	−0.00139	0.00778	0.0138	0.0254	0.00574	0.00525
	(0.0230)	(0.0212)	(0.0168)	(0.0212)	(0.0178)	(0.0170)	(0.0137)
Completed secondary education (census)	0.0396	0.0337	0.0264	0.105***	0.0670***	−0.0221	−0.0237
	(0.0256)	(0.0230)	(0.0181)	(0.0220)	(0.0197)	(0.0189)	(0.0156)
Birth Year (census)	−0.00231	−0.00442**	−0.00391**	−0.000805	-0.000908	0.00287	0.00213
	(0.00245)	(0.00207)	(0.00164)	(0.00237)	(0.00194)	(0.00189)	(0.00148)
Employed (census)	−0.0402	0.0195	0.00593	0.0283	0.0259	−0.00229	−0.00405
	(0.0288)	(0.0262)	(0.0205)	(0.0263)	(0.0212)	(0.0217)	(0.0177)
Married or cohabiting (census)	−0.000306	−0.0170	−0.0155	−0.00590	0.0113	0.0246	0.0156
	(0.0354)	(0.0334)	(0.0277)	(0.0340)	(0.0293)	(0.0281)	(0.0228)
BASELINE SAMPLE	YES	YES	NO	YES	NO	YES	NO
Observations	2.129	1.821	2.782	1.918	2,934	1.894	2.638
R-squared	0.181	0.354	0.338	0.231	0.139	0.152	0.134

Notes: Predictive OLS regressions of chlorination measures on psychological mechanisms, as well as on demographics. All regressors except those measured at baseline are potentially endogenous to treatment, and thus provide only correlational evidence. All regressions include village-level fixed effects, the demographic controls listed, and standard errors which are clustered at the level of the intervention cohort. Where available, we control for the baseline outcome of the dependent variable. Covariates are listed on the left, and are described in detail in Section 4. * denotes significance at 10 pct., ** at 5 pct., and *** at 1 pct. level.

TABLE D.3. Correlates of savings.

	Amount Saved Regularly (per week, KES)(10 weeks)		Amount Saved Regularly (per week, KES)(33 months)	
	(1)	(2)	(3)	(4)
Visualization	10.22	44.46	39.41	25.31
	(37.93)	(29.31)	(45.73)	(43.43)
Planning	−70.81	−19.57	20.31	16.76
	(46.71)	(23.18)	(41.64)	(41.97)
Active Control		45.60		−16.94
		(34.24)		(40.91)
Tower of London (baseline)	−2.990*			
	(1.697)			
β^{Effort} (baseline)	−3.112			
	(2.509)			
δ^{Effort} (baseline)	17.71			
	(30.80)			
General Self-Efficacy Score (GSE) (z-score) (baseline)	21.75			
	(14.41)			
Tower of London (10w)		−0.882	3.333	2.083
		(1.665)	(2.914)	(2.164)
β^{Effort} (10w)		−0.916	−17.51	−4.349
		(1.033)	(24.16)	(2.810)
δ^{Effort} (10w)		−17.65	1.577	1.090
		(14.41)	(31.70)	(22.23)
General Self-Efficacy Score (GSE) (z-score) (10w)		23.11***	42.09***	42.21***
		(8.341)	(13.48)	(11.76)
Utility Forecasting: Vividness (z-score) (33m)			53.00***	34.73**
			(20.08)	(16.57)
Utility Forecasting: Practice (z-score) (33m)			56.18***	36.65**
			(21.11)	(17.45)
Time Preferences, qualitative (z-score) (33m)			1.533	−1.490
			(17.31)	(13.91)
One-year discount factor (GPS staircase) (33m)			186.7	102.4
			(152.7)	(123.8)
High wealth index (census)	−6.586	−18.98	50.51	42.59
	(37.05)	(33.24)	(37.80)	(29.60)
Completed secondary education (census)	−5.398	13.84	116.1**	109.4***
	(47.60)	(34.25)	(48.84)	(40.63)
Birth Year (census)	−2.408	−2.539	0.208	0.122
	(4.435)	(3.163)	(4.119)	(3.185)
Employed (census)	80.75**	84.03**	53.64	107.2***
	(37.20)	(35.19)	(46.13)	(37.29)
Married or cohabiting (census)	−32.91	−0.689	18.22	1.447
	(76.21)	(61.38)	(54.24)	(46.36)
BASELINE SAMPLE	YES	NO	YES	NO
Observations	1.910	2.924	1.894	2.638
R-squared	0.136	0.070	0.167	0.135

Notes: Predictive OLS regressions of savings outcomes on psychological mechanisms, as well as on demographics. All regressors except those measured at baseline are potentially endogenous to treatment, and thus provide only correlational evidence. All regressions include village-level fixed effects, the demographic controls listed, and standard errors which are clustered at the level of the intervention cohort. Covariates are listed on the left, and are described in detail in Section 4. * denotes significance at 10 pct., ** at 5 pct., and *** at 1 pct. level.

TABLE D.4. Correlates of chlorination and savings: alternative mechanisms.

	Chlorine present in water (TCR) (12 weeks)		Amount Saved Regularly (per week, KES)(10 weeks)	
	(1)	(2)	(3)	(4)
Visualization	0.0491**	0.0571***	12.42	45.08
	(0.0216)	(0.0211)	(37.85)	(30.02)
Planning	0.0234	0.0351*	−51.50	−16.11
	(0.0228)	(0.0212)	(39.85)	(22.11)
Active Control		0.0227		48.20
		(0.0212)		(33.57)
Belief: Diarrhea avoided through chlorination (z score) (10w)	0.00544	0.00670	−11.58	−2.917
	(0.00904)	(0.00750)	(24.94)	(21.28)
Chlorine knowledge score (z-score) (10w)	0.0203**	0.0152**	−0.196	8.097
	(0.00910)	(0.00720)	(14.19)	(10.61)
Risk Aversion Measure (z-score) (10w)	0.00966	0.00795	−20.38	−22.17
	(0.00927)	(0.00769)	(12.59)	(13.79)
Total chlorine words remembered (10w)	−0.00181	0.00365	16.80	5.103
	(0.0110)	(0.00927)	(27.30)	(20.80)
Total saving words remembered (10w)	−0.00445	0.00117	55.86	23.74
	(0.0127)	(0.0106)	(35.86)	(29.34)
Total words remembered (10w)	0.000847	0.000754	−8.796	−3.922
	(0.00299)	(0.00256)	(6.637)	(6.178)
High wealth index (census)	−0.00894	0.00727	−14.28	−23.37
	(0.0203)	(0.0167)	(35.61)	(31.67)
Completed secondary education (census)	0.0339	0.0311*	20.17	27.22
	(0.0219)	(0.0180)	(41.54)	(32.32)
Birth Year (census)	−0.00467**	−0.00419***	−2.637	−1.859
	(0.00190)	(0.00162)	(3.824)	(3.134)
Employed (census)	0.0140	0.00756	86.34**	86.11**
	(0.0248)	(0.0208)	(34.19)	(34.92)
Married or cohabiting (census)	−0.00295	−0.0142	−49.00	2.180
	(0.0317)	(0.0276)	(74.34)	(61.79)
BASELINE SAMPLE	YES	NO	YES	NO
Observations	1.998	2.798	2.095	2.938
R-squared	0.367	0.336	0.123	0.072

Notes: Predictive OLS regressions of chlorination and savings measures on alternative mechanisms, as well as on demographics. All regressors except those measured at baseline are potentially endogenous to treatment, and thus provide only correlational evidence. All regressions include village-level fixed effects, the demographic controls listed, and standard errors which are clustered at the level of the intervention cohort. Covariates are listed on the left, and are described in detail in Section 4. * denotes significance at 10 pct., ** at 5 pct., and *** at 1 pct. level.

Appendix E: Pure Control Comparison

TABLE E.1. Behavioral outcomes (comparison with pure control group).

	MHT Level	Endline (10-12 weeks) (1) Pure Control Mean (SD)	(2) V+INF Treatment Effect	(3) P+INF Treatment Effect	(4) AC+INF Treatment Effect	(5) *N*	Follow-Up (30-36 months) (6) Pure Control Mean (SD)	(7) V+INF Treatment Effect	(8) P+INF Treatment Effect	(9) AC+INF Treatment Effect	(10) *N*
HEALTH OUTCOMES											
Objective measure: chlorine present in water (TCR)	1/-	0.22	0.06	0.04	0.03	2839					
		(0.42)	(0.02)***	(0.02)*	(0.02)						
Objective measure: chlorine sufficient to be safe (FCR)	3/-	0.18	0.06	0.03	0.03	2839					
		(0.39)	(0.02)***	(0.02)	(0.02 0						
			[0.01]**	[0.77]	[1.00]						
Main Treatment; Chlorine (self-report)	np/1	0.66	0.10	0.04	0.05	2984	0.88	−0.01	−0.01	-0.03	3194
		(0.47)	(0.03)***	(0.03)*	(0.03)**		(0.33)	(0.02)	(0.02)	(0.02)*	
			[0.00]***	[0.59]	[0.28]			[1.00]	[0.29]	[0.61]	
Main Treatment: Boil (self-report)	np/np	0.36	0.05	0.02	-0.01	2984	0.58	0.05	0.02	0.05	3194
		(0.48)	(0.03)*	(0.03)	(0.03)		(0.49)	(0.03)*	(0.03)	(0.03)**	
			[0.08]*	[1.00]	[1.00]			[0.91]	[1.00]	[0.37]	
Diarrhea incidences per child u15, last 3 months	3/1	0.24	−0.10	−0.08	-0.00	2815	0.29	−0.04	−0.07	-0.02	3130
		(0.73)	(0.04)***	(0.04)**	(0.04		(0.76)	(0.04)	(0.04)*	(0.04)	
			[0.02]**	[0.29]	[1.00]			[1.00]	[0.09]*	[1.00]	
Diarrhea incidences per child u5, last 3 months	np/3	0.28	−0.11	−0.03	0.03	2357	0.44	−0.05	−0.03	-0.03	2482
		(0.83)	(0.05)**	(0.05)	(0.05		(1.14)	(0.07)	(0.07)	(0.07)	
			[0.02]**	[1.00]	[1.00]			[1.00]	[1.00]	[1.00]	
Proportion of children taken for healthcare check-up	3/3	0.16	0.01	0.02	0.05	2796	0.36	0.00	0.00	0.02	3130
		(0.31)	(0.02)	(0.02)	(0.02)**		(0.37)	(0.02)	(0.02)	(0.02)	
			[0.46]	[1.00]	[0.25]			[1.00]	[1.00]	[1.00]	
Proportion of children u15 vaccinated, last 3 months	3/-	0.22	0.01	−0.02	-0.00	2790					
		(0.35)	(0.02)	(0.02)	(0.02)						
			[0.61]	[0.77]	[1.00]						
Number of ANC visits, last 3 months (if pregnant)	3/-	1.19	−0.22	0.05	0.26	272					
		(1.17)	(0.43)	(0.44)	(0.48)						
			[0.50]	[1.00]	[1.00]						
SAVINGS OUTCOMES											
Amount saved regularly (per week, KES)	2/2	88.76	16.62	7.67	3.09	2972	424.48	37.29	22.76	-18.28	3194
		(228.12)	(12.79)	(13.17)	(12.06)		(618.21)	(32.36)	(33.39)	(32.01)	
			[1.00]	[0.23]	[1.00]			[0.46]	[1.00]	[0.40]	
Indicator: Amount saved regularly is positive	3/3	0.32	0.12	0.01	0.04	2972	0.79	0.03	0.02	-0.01	3194
		(0.47)	(0.03)***	(0.03)	(0.03)		(0.41)	(0.02)	(0.02)	(0.02)	
			[0.00]***	[1.00]	[1.00]			[0.91]	[1.00]	[1.00]	
Number of ROSCAs [joined in last 3 months/total]	3/3	0.21	0.02	−0.00	-0.02	2972	1.05	0.12	0.09	0.01	3191
		(0.46)	(0.03)	(0.03)	(0.03)		(1.06)	(0.06)**	(0.06)*	(0.05)	
			[0.39	[1.00]	[1.00]			[0.86]	[1.00]	[1.00]	
Weekly ROSCA savings	np/np	202.18	36.60	16.03	5.50	2972	257.93	28.77	19.66	-12.71	3194
		(315.21)	(16.85)**	(17.11)	(16.68		(382.92)	(20.90)	(20.58)	(19.80)	
			[0.04]**	[1.00]	[1.00]			[0.46]	[1.00]	[0.40]	
Indicator: Saves for productive investments	3/3	0.17	0.10	0.01	0.02	2972	0.61	0.04	0.03	0.01	3194
		(0.38)	(0.02)***	(0.02)	(0.02)		(0.49)	(0.02)	(0.02)	(0.02)	
			[0.00]***	[1.00]	[1.00]			[0.91]	[1.00]	[1.00]	
Total Savings Balance (KES)	–/3						2984.17	556.07	−263.13	-522.20	3194
							(7912.65)	(424.38)	(391.31)	(388.07)	
								[0.98]	[1.00]	[1.00]	
LABOR OUTCOMES											
Total hours of work [last 3 months/last 7 days]	2/2	108.32	0.97	−21.77	-4.77	2972	14.45	0.98	−0.10	-1.35	3194
		(183.05)	(10.12)	(9.52)**	(9.78)		(17.25)	(0.88)	(0.87)	(0.86)	
			[1.00]	[0.07]*	[1.00]			[0.46]	[1.00]	[0.36]	
Total days of work, last 3 months	3/-	21.73	−0.07	−3.63	-1.17	2972					
		(30.45)	(1.72)	(1.62)**	(1.66)						
			[0.71]	[0.29]	[1.00]						
Earnings, cash and in-kind [monthly/last 7 days]	3/3	1167.22	6.11	−55.35	-105.20	2972	828.16	−82.87	−77.48	-204.74	3194
		(3155.76)	(168.76)	(174.31)	(177.88)		(1571.16)	(76.14)	(77.91)	(77.89)***	
			[0.71]	[1.00]	[1.00]			[1.00]	[1.00]	[0.14]	
OTHER BEHAVIORAL OUTCOMES											
Index of investment in children's education (z-score)	2/-	0.00	0.06	0.11	0.11	1967					
		(1.00)	(0.07)	(0.07)	(0.07)						
			[[1.00]	[0.14]	[0.64]						

Notes: OLS estimates of treatment effects, relative to the pure control group. For each variable, columns (1) and (6) report the mean and standard deviation of the control group. Columns (2)-(3) and (7)-(8) report the coefficients of interest and standard errors in parentheses. * denotes significance at 10 pct., ** at 5 pct., and *** at 1 pct. level. Square brackets contain additional p-values corrected for multiple hypothesis testing using the false discovery rate. All columns include village-level fixed effects, a vector of individual characteristics, fixed effects for the week and the day of the week of the relevant survey, and standard errors which are clustered at the level of the intervention cohort. Outcome measures are listed on the left, and are described in detail in Section 4.

TABLE E.2. Psychological outcomes (comparison with pure control group).

		Endline (10-12 weeks)					Follow-Up (30-36 months)				
	MHT Level	(1) Pure Control Mean (SD)	(2) V+INF Treatment Effect	(3) P+INF Treatment Effect	(4) AC+INF Treatment Effect	(5) N	(6) Pure Control Mean (SD)	(7) V+INF Treatment Effect	(8) P+INF Treatment Effect	(9) AC+INF Treatment Effect	(10) N
PLANNING SKILLS											
BADS score (z-score)	1/-	0.00	0.03	0.12	0.05	2955					
		(1.00)	(0.06)	(0.06)**	(0.06)						
			[1.00]	[0.08]*	[1.00]						
Tower of London (z-score)	2/-	0.00	−0.36	−0.40	-0.34	2955					
		(1.00)	(0.05)***	(0.05)***	(0.05)***						
			[0.00]***	[0.00]***	[0.00]***						
TIME PREFERENCES											
β^{Effort}	1/-	0.953	0.007	0.012	0.009	2906					
		(0.020)	(0.018)	(0.018)	(0.018)						
			[1.00]	[0.32]	[1.00]						
δ^{Effort}	2/-	0.995	−0.001	−0.002	0.003	2906					
		(0.002)	(0.002)	(0.002)	(0.002)						
			[0.46]	[0.22]	[0.23]						
Utility Forecasting: Vividness (z-score)	–/1						−0.00	0.06	−0.07	-0.02	3191
							(1.00)	(0.05)	(0.05)	(0.05)	
								[0.74]	[0.12]	[1.00]	
Utility Forecasting: Practice (z-score)	–/2						0.00	0.01	0.04	-0.08	3191
							(1.00)	(0.05)	(0.05)	(0.05)	
								[1.00]	[1.00]	[0.35]	
β^{MPL}	1/-	1.02	−0.01	0.01	0.00	2955					
		(0.43)	(0.03)	(0.03)	(0.02)						
			[0.46]	[0.29]	[0.50]						
δ^{MPL}	2/-	0.98	0.00	0.00	0.00	2955					
		(0.02)	(0.00)*	(0.00)*	(0.00)**						
			[0.09]*	[0.11]	[0.05]**						
Time Preferences, qualitative (GPS)	–/2						0.00	0.12	−0.01	0.09	3191
							(1.00)	(0.05)**	(0.05)	(0.05)*	
								[0.06]*	[1.00]	[0.35]	
One-year discount factor (GPS staircase)	–/2						0.52	0.00	−0.01	0.00	3191
							(0.12)	(0.01)	(0.01)	(0.01)	
								[1.00]	[1.00]	[0.36]	
SELF-EFFICACY											
General Self-Efficacy Score (GSE) (z-score)	2/1	0.00	0.17	0.17	0.02	2955	−0.00	−0.01	−0.13	-0.05	3191
		(1.00)	(0.07)***	(0.06)***	(0.06)		(1.00)	(0.05)	(0.05)***	(0.05)	
			[0.02]**	[0.01]***	[0.50]						

Notes: OLS estimates of treatment effects, relative to the pure control group. For each variable, columns (1) and (6) report the mean and standard deviation of the control group. Columns (2)-(3) and (7)-(8) report the coefficients of interest and standard errors in parentheses. * denotes significance at 10 pct., ** at 5 pct., and *** at 1 pct. level. Square brackets contain additional p-values corrected for multiple hypothesis testing using the false discovery rate. All columns include village-level fixed effects, a vector of individual characteristics, fixed effects for the week and the day of the week of the relevant survey, and standard errors which are clustered at the level of the intervention cohort. Outcome measures are listed on the left, and are described in detail in Section 4.

TABLE E.3. Alternative mechanisms (comparison with pure control group).

	Endline (10-12 weeks)					Follow-Up (30-36 months)				
	(1) Pure Control Mean (SD)	(2) V+INF Treatment Effect	(3) P+INF Treatment Effect	(4) AC+INF Treatment Effect	(5) *N*	(6) Pure Control Mean (SD)	(7) V+INF Treatment Effect	(8) P+INF Treatment Effect	(9) AC+INF Treatment Effect	(10) *N*
BELIEFS AND KNOWLEDGE										
Belief: Diarrhea avoided through chlorination (z-score)	0.00	0.13	0.15	0.11	2955					
	(1.00)	(0.06)**	(0.05)***	(0.06)**						
Chlorine knowledge score (z-score)	0.00	0.11	0.06	0.08	2955					
	(1.00)	(0.05)**	(0.06)	(0.06)						
ANC/PNC knowledge score (z-score)	0.00	0.34	0.28	0.33	2955					
	(1.00)	(0.06)***	(0.05)***	(0.05)***						
Risk Aversion Measure (z-score)	0.00	0.01	–0.01	0.04	2735					
	(1.00)	(0.06)	(0.06)	(0.06)						
SALIENCE TASK										
Chlorine word remembered	0.38	0.08	0.05	0.02	8934	0.43	0.08	0.03	0.00	3191
	(0.48)	(0.01)***	(0.01)***	(0.01)		(0.50)	(0.03)**	(0.03)	(0.02)	
Savings word remembered	0.44	0.00	0.02	0.01	8934	0.48	0.01	-0.03	–0.03	3191
	(0.50)	(0.01)	(0.01)	(0.01)		(0.50)	(0.02)	(0.02)	(0.02)	
Total words remembered	4.23	–0.12	–0.01	-0.04	8934	4.50	0.19	0.09	–0.06	3191
	(1.66)	(0.08)	(0.08)	(0.07)		(1.60)	(0.08)*	(0.08)	(0.08)	

Notes: OLS estimates of treatment effects, relative to the pure control group. For each variable, columns (1) and (6) report the mean and standard deviation of the control group. Columns (2)-(3) and (7)-(8) report the coefficients of interest and standard errors in parentheses. * denotes significance at 10 pct., ** at 5 pct., and *** at 1 pct. level. All columns include village-level fixed effects, a vector of individual characteristics, fixed effects for the week and the day of the week of the relevant survey, and standard errors which are clustered at the level of the intervention cohort. Outcome measures are listed on the left, and are described in detail in Section 4. The bottom panel of the table reports the probability of remembering a chlorine-related word, or a savings-related word, on a given word list in the salience task. In the endline survey, participants were read three word lists, resulting in three observations per individual. In the long-run follow-up, participants were read one word list (randomly selected out of three lists). Salience regressions additionally control for the total number of words the participant remembered on that word list.

Appendix F: Test Corrections and Experimental Integrity

Table F.1 lists our hypotheses and pre-specified outcome variables. We adjust for multiple hypothesis testing within outcome groups (behaviors and psychological mechanisms) and hierarchical outcome categories (primary, secondary, and exploratory), but not across them.

Behavioral outcomes are our main focus. Our primary hypothesis is that interventions affect water chlorination, measured with the primary outcome of objectively measured water chlorination (10 weeks) and self-reported chlorination (30-36 months). In the follow-up, we add another primary hypothesis, that the intervention affects chlorination-related health outcomes, and measure child diarrhea. After 30-36 months, we correct p-values across these two primary hypotheses.

Our secondary hypothesis tests if the interventions have domain-general effects on future investments. We consider one pre-specified outcome measuring savings behavior, labor supply, and education investment (10 weeks) and savings behavior and labor supply (30-36 months). We adjust p-values across this group of outcomes in each round.

For analysis on psychological outcomes, after 10 weeks, we test three main hypotheses, namely that interventions affect planning, time preferences and self-efficacy, with one primary variable to capture each concept. We correct p-values over the three hypotheses. After 30-36 months, we only examine time preferences and self-efficacy, as we found few short term effects on planning measures. We also run exploratory analysis on some pre-specified and some non-specified variables. We correct across all the exploratory tests we run on behaviors, and separately, on psychological outcomes.

TABLE F.1. Primary and secondary hypotheses by round.

	10 weeks	30-36 months
BEHAVIOR		
**Hypothesis 1: the intervention affects water chlorination**		
Presence of any chlorine in household drinking water	Primary	
General water treatment: Indicator for chlorine	Not pre-specified	Primary
**Hypothesis 1a: the intervention affects health outcomes**		
Number of diarrhea episodes per child under 15 in last 3 months	Exploratory	Primary
**Hypothesis 2: the intervention affects future investments**		
Amount saved regularly (weekly, KES)	Secondary	Secondary
Total hours of work[1]	Secondary	Secondary
Education investment index[2]	Secondary	Not measured
PSYCHOLOGICAL MECHANISMS		
**Hypothesis 1: the intervention affects planning ability**		
Behavioral Activation for Depression Scale - Short Form (BADS-SF)	Primary	Not measured
Tower of London task: total moves across all four rounds	Secondary	Not measured
**Hypothesis 2: the intervention affects time preferences**		
β^{Effort} (estimated from the effort discounting task)	Primary	Not measured
Utility forecasting: Vividness rating	Not measured	Primary
**Hypothesis 3: the intervention affects self-efficacy**		
Generalized Self-Efficacy (GSE) scale	Secondary	Primary

[1] Hours of work is measured over the last 3 months in the 10-week survey and last 7 days in 30-36 month survey.
[2] Index consists of school days missed across all children, and schooling expenditure per child in last three months.

TABLE F.2. Summary of participation.

	Pure Control	Active Control	Visualization	Planning	Total
Recruited	775	992	992	991	3750
Completed baseline	–	777	783	777	2337
Completed baseline & Intervention 1	–	777	783	777	2337
Completed baseline & Intervention 1 & Intervention 2	–	736	734	711	2181
Completed baseline & endline	-	716	708	692	2116
Completed endline	588	811	800	785	2984
Completed baseline & endline & chlorine test	-	662	647	641	1950
Completed endline & chlorine test	568	771	754	738	2831
Completed chlorine test	571	771	756	741	2839
Completed follow-up survey	649	849	848	849	3195
Completed baseline & follow-up survey	–	697	692	684	2073
Completed baseline & endline & follow-up survey	–	652	637	627	1916

TABLE F.3. Baseline balance: main outcomes.

	Comparison with active control (AC+INF)				
	(1) Active Control Group Mean (SD)	(2) Visualization Treatment Effect	(3) Planning Treatment Effect	(4) Column 2 vs. Column 3 p-value	(5) N
Baseline score					
Tower of London: Total Moves	23.32	−0.48	−0.30	0.70	2197
	(6.83)	(0.46)	(0.42)		
		[0.98]	[1.00]		
General Self-Efficacy Score (GSE)	42.84	−0.07	0.90	0.19	2175
	(11.59)	(0.68)	(0.74)		
		[1.00]	[1.00]		
β^{MPL}	0.99	0.01	0.00	0.64	2142
	(0.37)	(0.02)	(0.02)		
		[1.00]	[1.00]		
δ^{MPL}	0.98	0.00	0.00	0.52	2142
	(0.02)	(0.00)	(0.00)		
		[1.00]	[1.00]		
Self report: added chlorine in last month	0.65	−0.04	−0.04	0.82	2184
	(0.48)	(0.03)	(0.03)		
		[0.56]	[1.00]		
Chlorine knowledge score (z-score)	0.00	0.09	0.01	0.18	2337
	(1.00)	(0.05)	(0.05)		
		[0.56]	[1.00]		
Prevalence of diarrhea among u15 children, last 2 weeks	0.09	0.01	0.00	0.63	2056
	(0.24)	(0.01)	(0.01)		
		[0.41]	[0.82]		

Notes: OLS estimates of baseline values of main outcomes. For each variable, column (1) reports the mean and standard deviation of the control group. Columns (2)-(3) report the coefficients of interest and standard errors in parentheses. Square brackets contain additional p-values corrected for multiple hypothesis testing using the false discovery rate. All columns include village-level fixed effects, control for a vector of individual characteristics, and cluster standard errors at the level of the intervention cohort. * denotes significance at 10 pct., ** at 5 pct., and *** at 1 pct. level. Outcome measures are listed on the left, and are described in detail in Section 4. The Tower of London is a lab game that measures a participant's ability to plan ahead. The General Self-Efficacy score measures a participant's belief in their own ability to achieve the outcomes they desire. Time preference parameters β and δ measured over money are derived from responses to Multiple Price Lists (MPL).

TABLE F.4. Baseline balance: dispenser vs. non-dispenser villages .

	(1) Village without Chlorine Dispenser Mean (SD)	(2) Village with Chlorine Dispenser Difference	(3) N
Observables			
Age	26.25	0.21	3750
	(4.68)	(0.15)	
Married/ Cohabiting	0.89	−0.01	3750
	(0.31)	(0.01)	
Education Level	5.84	0.10	3750
	(1.18)	(0.04)***	
High Wealth Index	0.51	0.01	3750
	(0.50)	(0.02)	

Notes: OLS estimates of baseline balance on observed characteristics for villages with and without WASH chlorine dispensers. For each variable, we report the mean of villages without a chlorine dispenser, with the standard deviation in parentheses. Column (2) reports the difference for villages with a chlorine dispenser, with standard errors in parentheses. All standard errors are clustered at the level of the intervention cohort. * denotes significance at 10 pct., ** at 5 pct., and *** at 1 pct. level.

TABLE F.5. Attrition analysis: treatments vs. active control.

	(1) Not in endline	(2) Not in chlorine test	(3) Not in follow-up	(4) Not in endline	(5) Not in chlorine test	(6) Not in follow-up	(7) Not in endline	(8) Not in chlorine test	(9) Not in follow-up
Visualization	0.02	0.01	0.01	0.02	0.01	0.01	0.02	-0.05	0.12
	(0.02)	(0.02)	(0.01)	(0.02)	(0.02)	(0.01)	(0.12)	(0.14)	(0.12)
Planning	0.03	0.03	0.02	0.03	0.03	0.02	0.14	0.15	0.01
	(0.02)*	(0.02)	(0.01)	(0.02)*	(0.02)	(0.01)	(0.14)	(0.15)	(0.12)
Age				-0.01	–0.01	–0.01	–0.01	-0.01	–0.01
				(0.00)***	(0.00)***	(0.00)***	(0.00)***	(0.00)**	(0.00)***
Married or cohabiting				-0.03	–0.02	0.00	0.00	0.01	–0.07
				(0.02)	(0.02)	(0.03)	(0.03)	(0.04)	(0.04)
Education level				-0.00	–0.01	–0.00	–0.00	-0.01	0.01
				(0.01)	(0.01)	(0.01)	(0.01)	(0.01)	(0.01)
V x Age Interaction							0.00	0.00	–0.00
							(0.00)	(0.00)	(0.00)
P x Age Interaction							–0.00	-0.00	–0.00
							(0.00)	(0.00)	(0.00)
V x Married Interaction							–0.09	-0.08	0.10
							(0.05)*	(0.05)	(0.06)*
P x Married Interaction							–0.01	-0.01	0.12
							(0.06)	(0.06)	(0.06)**
V x Education Interaction							0.01	0.00	–0.02
							(0.01)	(0.01)	(0.01)
P x Education Interaction							–0.01	-0.01	–0.01
							(0.01)	(0.02)	(0.01)
V x Wealth Interaction							–0.01	0.00	0.03
							(0.03)	(0.04)	(0.03)
P x Wealth Interaction							–0.03	-0.01	0.02
							(0.03)	(0.04)	(0.03)
Constant	0.09	0.15	0.13	0.31	0.39	0.41	0.27	0.37	0.38
	(0.01)***	(0.02)***	(0.01)***	(0.06)***	(0.07)***	(0.05)***	(0.08)***	(0.10)***	(0.08)***
Observations	2337	2337	2337	2337	2337	2337	2337	2337	2337

Notes: OLS estimates of the probability of attriting relative to the active control group. For each variable, we report the coefficients of interest, and standard errors in parentheses. Each column represents a different specification, with or without controls and interaction terms to assess whether i) there was differential attrition for groups with certain observed characteristics (columns (4)–(6)) and ii) there was any differential effect of an observed characteristic on the probability of attriting for any treatment group (columns (7)–(9)). All standard errors are clustered at the level of the intervention cohort. * denotes significance at 10 pct., ** at 5 pct., and *** at 1 pct. level.

Table F.6. Attrition analysis: active treatments vs. pure control.

	(1) Not in endline	(2) Not in chlorine test	(3) Not in follow-up	(4) Not in endline	(5) Not in chlorine test	(6) Not in follow-up	(7) Not in endline	(8) Not in chlorine test	(9) Not in follow-up
V+INF	–0.05	–0.04	–0.01	-0.06	–0.04	–0.02	–0.02	0.01	0.21
	(0.02)***	(0.02)*	(0.02)	(0.02)***	(0.02)*	(0.02)	(0.16)	(0.17)	(0.13)*
P+INF	–0.04	–0.02	–0.01	-0.04	–0.02	–0.02	–0.04	0.01	0.14
	(0.02)*	(0.02)	(0.02)	(0.02)**	(0.02)	(0.02)	(0.18)	(0.18)	(0.14)
AC+INF	–0.06	–0.04	–0.01	-0.06	–0.04	–0.01	–0.16	-0.09	0.14
	(0.02)***	(0.02)*	(0.02)	(0.02)***	(0.02)**	(0.02)	(0.17)	(0.17)	(0.13)
Age				-0.01	–0.01	–0.01	–0.01	-0.01	–0.01
				(0.00)***	(0.00)***	(0.00)***	(0.00)**	(0.00)*	(0.00)**
Married or cohabiting				-0.08	–0.08	0.02	–0.10	-0.13	0.04
				(0.02)***	(0.02)***	(0.02)	(0.06)*	(0.06)**	(0.05)
Education level				-0.01	–0.01	–0.00	–0.01	-0.01	0.01
				(0.01)**	(0.01)**	(0.00)	(0.01)	(0.01)	(0.01)
V+INF x Age Interaction							–0.00	-0.00	–0.00
							(0.00)	(0.00)	(0.00)
P+INF x Age Interaction							0.00	0.00	–0.00
							(0.00)	(0.00)	(0.00)
AC+INF x Age Interaction							0.00	-0.00	0.00
							(0.00)	(0.00)	(0.00)
V+INF x Married Interaction							0.01	0.03	–0.00
							(0.07)	(0.08)	(0.06)
P+INF x Married Interaction							0.04	0.08	–0.01
							(0.08)	(0.08)	(0.06)
AC+INF x Married Interaction							0.05	0.09	–0.08
							(0.07)	(0.08)	(0.06)
V+INF x Education Interaction							0.00	-0.01	–0.02
							(0.02)	(0.02)	(0.01)
P+INF x Education Interaction							–0.01	-0.02	–0.02
							(0.02)	(0.02)	(0.01)
AC+INF x Education Interaction							0.00	-0.00	–0.01
							(0.02)	(0.02)	(0.01)
V+INF x Wealth Interaction							0.00	-0.00	0.01
							(0.04)	(0.04)	(0.04)
P+INF x Wealth Interaction							–0.02	-0.02	0.01
							(0.04)	(0.04)	(0.04)
AC+INF x Wealth Interaction							0.02	0.00	–0.02
							(0.04)	(0.04)	(0.04)
Constant	0.26	0.29	0.19	0.59	0.62	0.44	0.61	0.61	0.29
	(0.02)***	(0.02)***	(0.02)***	(0.06)***	(0.06)***	(0.05)***	(0.13)***	(0.14)***	(0.10)***
Observations	3750	3750	3750	3750	3750	3750	3750	3750	3750

Notes: OLS estimates of the probability of attriting relative to the pure control group. For each variable, we report the coefficients of interest, and standard errors in parentheses. Each column represents a different specification, with or without controls and interaction terms to assess whether i) there was differential attrition for groups with certain observed characteristics (columns (4)–(6)) and ii) there was any differential effect of an observed characteristic on the probability of attriting for any treatment group (columns (7)–(9)). All standard errors are clustered at the level of the intervention cohort. * denotes significance at 10 pct., ** at 5 pct., and *** at 1 pct. level.

Appendix G: Detailed Descriptions of Outcome Measures

G.1. Tower of London Planning Task

In our computerized version of the task, participants see a screen with two parts: on the left side is the word "start" with a picture of three "pegs" and various shapes positioned on the pegs; on the right side is the word "goal" with a similar picture of three "pegs" and the same shapes positioned differently on the pegs. To complete the task, participants must reposition the shapes underneath the "start" on the left to match the "goal" position on the right. They are instructed to complete each round in as few moves as possible, with the minimum number of moves shown as a number on the screen. In addition to a practice round, participants attempt four rounds of increasing complexity, beginning with one shape requiring only one move, and concluding with three shapes in a pattern that necessitates at least four moves. In all rounds, participants are limited to a maximum of 10 moves. If this occurs, the round ends and the participant is required to contact a staff member to ensure she understands the task before continuing to the next round. Therefore, the distribution of scores is censored at both ends. Performance on the Tower of London task is computed as the total number of moves used across the four rounds. An example of the participant's screen is shown in Figure G.1. Payment was based on a randomly selected round, with a payment of KES 250 for completing the task with the minimum number of moves, and a KES 50 deduction for each additional move.

G.2. Effort Discounting Task

Following recent innovations in the elicitation of time preferences (Andreoni and Sprenger 2012; Augenblick et al. 2015), we estimate time preferences in the effort domain, using the methodology of Augenblick (2017): participants choose how many units of an effort task they want to complete at a time t for a piece rate w, where t is 0, 1, 7, or 8 days from today, and the piece rate w is KES 2, 6, or 10. Variation in time identifies the discount rate, while variation in piece rates identifies the curvature of the utility function. One time and one piece rate are randomly implemented at the end (described below). Figure G.2 provides an example of the participant interface for the task.[1] In contrast to Augenblick (2017), we hold the time of decision constant and vary the time of effort provision, which requires us to control for weekday effects. All questions required a minimum effort allocation of one task at each time to control for the fixed costs of starting, and allow a maximum of 50 tasks.

Developing an effort task that is adapted to a field setting in a developing country, with low levels of literacy, was challenging: the required variation in timing meant that effort could not be completed in the laboratory. We needed to monitor and enforce *when* participants supply effort, and *how much*, while they are in their homes, and do

1. To consider the possibility that respondents feel obligated to carry out some effort regardless of the wage, a subsample of participants was also asked how many units of effort they would supply for a piece rate of KES 0 (but still receiving the KES 100 completion bonus explained below).

not have access to a computer. We thus developed a new effort task that is adapted to our setting: participants completed data entry tasks by SMS, using toll-free numbers administered by the Busara Center.[2] Each SMS was supposed to contain a 30-digit random number string, which takes approximately two minutes to type. Participants were given a sheet with 50 such strings, including a counter to keep track. To ensure comprehension, participants completed one practice SMS during the survey. At the end of the survey, one decision (out of 12) was randomly selected to be the "decision that counts": at the selected piece rate and time horizon, participants had to send the exact number of SMS they chose. If they did, they received the full piece rate payment plus a KES 100 completion bonus. If they failed to implement the decision they made, they lost both the payment for this task and the completion bonus (see Augenblick (2017) for a full description of this method).[3] Earnings from this task were paid 14 days from the survey date, regardless of the selected effort time horizon.

We estimate time preferences over effort following the approach of Augenblick (2017) by assuming quasi-linear utility (linear in money, convex in effort) and a power cost of effort function. We additionally assume quasi-hyperbolic discounting. Following DellaVigna and Pope (2017), we allow for a non-monetary reward s, which participants receive for each task in addition to the piece rate. The non-monetary reward captures a range of motives, from norm or sense of duty, to reciprocity towards the employer (for the flat payment), to intrinsic motivation and personal competitiveness. It was motivated by the observation that participants supply non-zero amounts of effort even for low piece rates (DellaVigna and Pope 2017). The optimal level of effort is thus given by

$$e^* = \text{argmax}\,(s + D_m(14)\cdot\varphi\cdot w)\cdot e - \beta^{I(t>0)}\cdot\delta^t\cdot((1/\gamma)e^\gamma + d_w\cdot e) \qquad \text{(G.1)}$$

where β and δ capture (hyperbolic) temporal discounting of effort, w is the piece rate, $D_m(14)$ captures monetary discounting of the payment in 14 days (this is constant for all questions, and thus allowed to differ from effort discounting), t is the time of effort provision, $\gamma > 1$ captures convex costs of effort, φ is a slope parameter, and d_w are weekday indicators which allow the opportunity cost of time to vary across weekdays. Within the non-linear objective function above, we estimate additive treatment effects of V, P, and AC on the parameters β, δ, s, and γ.[4]

2. Although we did not screen on phone access, all participants in our sample have access to a mobile phone: 71% own one, 96% have one in their household, and the remainder shares the phone of friends or relatives. Since phones are often used by multiple individuals, phone access should be understood as continuous rather than binary.

3. The field setting with SMS required some tolerance: while a laboratory computer can confirm correct and incorrect entries, and display the number of tasks still to complete, we relied on participants to do this themselves. We thus allowed for 75% accuracy in entering the number strings, and a tolerance of 10 in the number of completed SMS (subject to positive completion). The permissible time window started on the calendar day before the task was due, and ended with the calendar day of the task itself. Participants were told that there would be some tolerance for mistakes, but not how much.

4. To control for the effect of introducing a 0 KES piece rate for a subsample of participants (Footnote 1), we additionally allow treatment effects of being exposed to the zero rate on the parameters γ and s. These parameters measure the intercept and curvature of the effort supply function, and may thus be affected by variations in the set of wages. In contrast, time preferences are assumed to be orthogonal to wage variation effects.

Sixty-six percent of participants identifiably sent at least one SMS (that was not a practice SMS during the session), 60% sent the correct number of SMS during the correct time window, and 41% additionally satisfied the required accuracy threshold (see footnote 3) and got paid. The key challenge for the verification of the effort task was matching SMS to participants: despite various safety provisions (including name and subject ID in each SMS, asking participants to report all phone numbers they might use), 59049 SMS from 3144 phone numbers could not be matched to any of our 2983 participants. This challenge arises from a field setting where individuals commonly share multiple phones within or across households (see footnote 2).

To test for difficulties in access to phones, we included a small module in the endline survey in which participants were asked about their access to a mobile phone, particularly at the times necessary to complete the SMS task. To alleviate the concern that respondents did not understand the payment scheme, we included three multiple-choice comprehension questions immediately before the task that asked participants to calculate the payout in different circumstances. Respondents could not participate in the task until they had answered the comprehension questions correctly.

Table G.4 shows phone access and task comprehension by treatment group. We find high rates of phone access and comprehension across all treatment groups, and no large differences across treatment groups. The exception is the pure control group, which showed lower comprehension at endline compared to the active treatment groups, presumably because it was their first time completing the task, while the other groups had already experienced it at baseline. We therefore interpret differences in time preferences between this group and the others with caution.

G.3. Money Discounting Task

In addition to the effort discounting task, we included a conventional Multiple Price List (MPL) task to measure monetary discounting. Participants were asked to make 10 choices between payments at earlier and later dates. The payment at the early date was always KES 100, while the payment at the later date increased gradually from KES 110 to KES 300, using gross interest rates 1.1, 1.25, 1.75, 2, and 3. Each decision was first made in a near time-frame (today vs. four weeks from today), and later in a future time-frame (four weeks vs. eight weeks from today). The list of decisions is presented in Table G.1. One decision was randomly selected to be paid out. As outcome measures from the MPL we estimate β and δ in the quasi-hyperbolic discounting model of Laibson (1997), assuming linearity of utility in money.

G.4. Alternative Mechanisms

Beliefs About Effectiveness of Chlorination. We assess differential beliefs across treatment groups about the proportion of pediatric diarrhea cases which can be prevented by water chlorination. At baseline, all participants in the active treatment groups (“Visualization,” “Planning,” and “AC+INF”) are told that water chlorination reduces childhood diarrhea by approximately one third. At endline they are asked this

question in a multiple-choice format. We take the proportion of cases the participant believes chlorine can avert as a measure of belief about chlorine effectiveness.

Knowledge of How to Use Chlorine. We assess differential knowledge across treatment groups of how to use chlorine to sanitize water. We ask two multiple-choice questions at endline, to which all three active treatment groups were told the correct answer at baseline: (i) how much chlorine to add to water; (ii) the amount of time that needs to pass after chlorine is added for water to be safe to drink.

Risk Preferences. We include a modified Eckel-Grossman task to account for changes in risk preferences (Charness et al. 2013). Participants choose between one of three 50/50 lotteries, represented as bets on a coin flip. We construct an ordinal measure of risk aversion based on the expected payout the participant is willing to forgo for an increase in certainty of payout.

Salience of Chlorination. We test for the possibility that our treatments differentially increased the salience of water chlorination. During the endline survey, enumerators read out three lists of nine words each to every participant, and asked her to recall as many words as possible directly after reading each list. Participants were paid KES 5 for every word they remembered. Each list contained three categories of future-related words (chlorine, savings, and farm investment), as well as non-future related filler words. The word lists are available in original Swahili and English translation in Table G.2. We estimate salience effects using equation G.2:

$$w_{im} = a_0 + \sum_{j=1}^{3} a_j T_{ji} + \psi_0 X_{im} + \delta_m + \theta_{im} \tag{G.2}$$

where w_{im} is an indicator for participant i correctly recalling the word related to chlorine in list m; X_{im} refers to the number of words that the individual correctly recounted from that list; δ_m is a fixed effect for list m; and T_j are treatment indicators. We test $H_0 : \alpha_1 = \alpha_2 = \alpha_3$, with the null hypothesis corresponding to no differential salience of chlorine across (active) treatment groups.

In case our treatments differentially affected the salience of chlorine, we further test whether this is due to an increased salience of future-oriented behaviors in general - which may result from our main psychological mechanisms of interest. To this end, we estimate whether the differential treatment effect on chlorine words also holds for two other future-oriented behaviors (saving and farm investment), which were not emphasized in the sessions. We estimate

$$w_{imn} = a_0 + \sum_{j=1}^{3} a_j T_{ji} + \lambda chlorine_n + \psi X_{im} + \sum_{k=1}^{3} b_k T_{ki} \cdot chlorine_n + \delta_m + \theta_{imn} \tag{G.3}$$

where w_{imn} is an indicator for participant i correctly recalling the words in list m from future oriented behavior n (chlorination, savings or farm investment); and $chlorine_n$ is a

dummy for the word being related to chlorine. The a_j coefficients capture increased future orientation due to treatment, while the b_j coefficients indicate that salience increased differentially for chlorination. We test $H_o : b_1 = b_2 = b_3$, with the null hypothesis corresponding to no differential salience of chlorine across (active) treatments.

G.4.1. Schedule of Tasks and Treatments. Participants were assigned randomly to attend baseline and intervention sessions either in the morning or in the afternoon. While participants were encouraged to attend the session type assigned to them, they were allowed to switch to the other session time if necessary to minimize attrition. Within a geographical region and within each treatment group, participants were invited to sessions in alphabetical order, based on the first letter of their last name. Participants were invited to a 7:30AM or 12:30PM session at a village hall in their area. Sessions lasted between two and four hours. Participants received short breaks between each item on the agenda.

During zTree portions of the session, each participant sat in front of a Windows tablet computer, sufficiently spaced to prevent participants from seeing the answers of their neighbors. One enumerator read instructions and answer options aloud in Kiswahili from the center of the room, while several others were available to answer individual questions or assist with the technology.

During the SurveyCTO questionnaires at endline, five to eight enumerators went through questionnaires with participants individually, in the order that participants arrived.

Interventions were carried out in cohorts of approximately five, in a circle outside when weather permitted. Groups were physically separated to ensure participants could not be overheard. All participants received the same intervention on a given day.

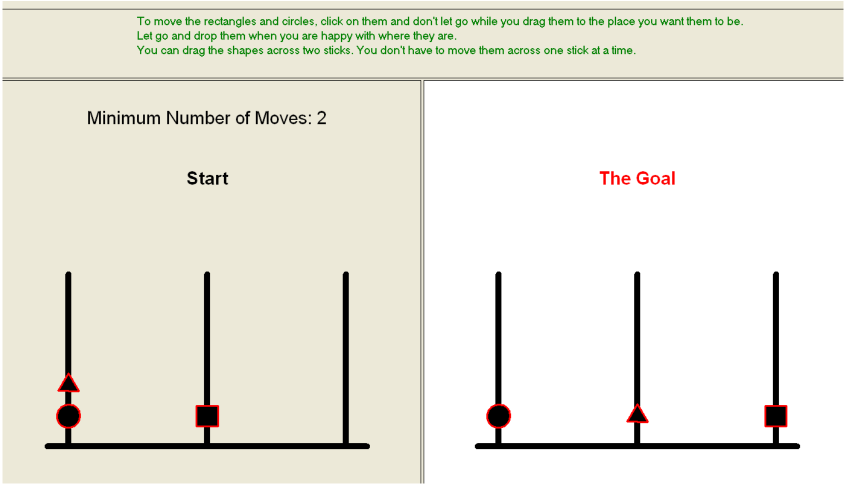

FIGURE G.1. Tower of London example screen.

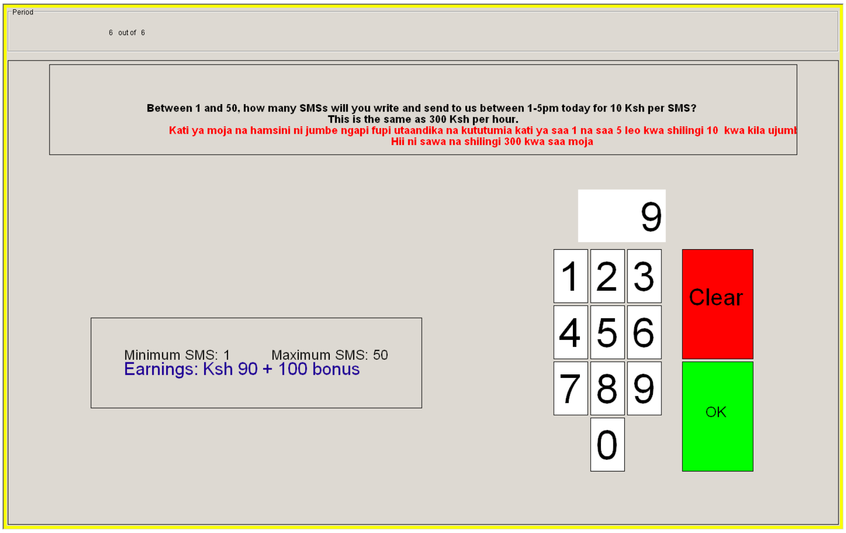

FIGURE G.2. Effort discounting task example screen.

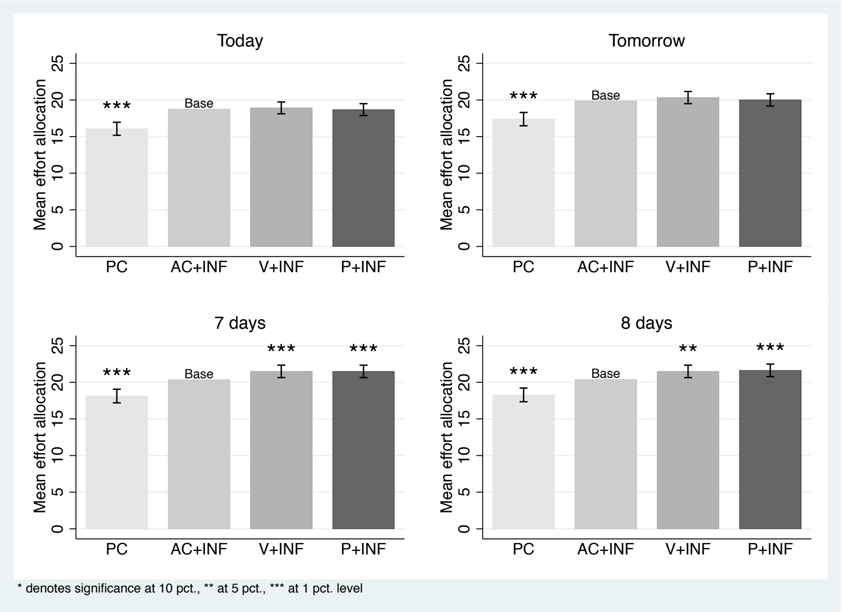

FIGURE G.3. Effort discounting: raw responses.

TABLE G.1. Monetary discounting decisions.

Front-end delay (t)	Delay between payments (k)	Early (m)	Maximum Late ($m(1+r)$)	Implied interest rate ($1+r$)
**Frame 1**				
0	28	100	110	1.1
0	28	100	125	1.25
0	28	100	175	1.75
0	28	100	200	2
0	28	100	300	3
**Frame 2**				
28	28	100	110	1.1
28	28	100	125	1.25
28	28	100	175	1.75
28	28	100	200	2
28	28	100	300	3

Table G.2. Word lists for salience test.

List	Position	English Translation	Swahili	Group
A	1	Fence	Fence	Filler
A	2	Panadol	Panadol	Filler
A	3	WaterGuard	WaterGuard	Chlorine
A	4	Playing	Kucheza	Filler
A	5	Saving	Kuwekeza	Saving
A	6	Tarmac	Lami	Filler
A	7	Dairy Cow	Ng'ombe wa maziwa	Farm Investment
A	8	Safaricom	Safaricom	Filler
A	9	Resting	Kupumzika	Filler
B	1	Patterned Cloth	Kitenge	Filler
B	2	Thermos	Thermos	Filler
B	3	Savings Group	Chama	Savings
B	4	Baby Oil	Mafuta ya mtoto	Filler
B	5	Poultry Farming	Kilimo cha kuku	Farm investment
B	6	Petrol	Petroli	Filler
B	7	Chlorine	Klorini	Chlorine
B	8	Machete	Panga	Filler
B	9	Shoe Polish	Rangi ya viatu	Filler
C	1	Saucepan	Sufuria	Filler
C	2	Stool	Stool	Filler
C	3	Farm Lease	Kukodisha shamba	Farm investment
C	4	Transport	Transport	Filler
C	5	Dispenser	Dispensa	Chlorine
C	6	Photocopier	Photocopier	Filler
C	7	Piggybank	Benki ya nyumbani	Savings
C	8	Airtime	Airtime	Filler
C	9	Community Hall	Ukumbi wa jamii	Filler

TABLE G.3. Raw means of z-scored outcome variables.

	(1) Active Control Group Mean (SD)	(2) Pure Control Mean (SD)
Behavioral Activation score (BADS) (9–63)	29.27	28.90
	(5.83)	(5.67)
Tower of London: Total moves (9–36)	21.29	23.86
	(6.71)	(6.79)
Risk aversion measure (1–3)	1.74	1.70
	(0.82)	(0.83)
General Self-Efficacy score (GSE) (0–72)	43.43	43.32
	(11.90)	(9.83)
Belief: Proportion of diarrhea incidences avoided through chlorination (0–1)	0.75	0.71
	(0.39)	(0.41)
Chlorine knowledge score (0–2)	1.12	1.07
	(0.69)	(0.69)
ANC/PNC knowledge score (0–2)	1.21	0.93
	(0.85)	(0.87)
Index of investment in children's education (-3–3)	0.04	−0.07
	(1.14)	(0.95)

Notes: Mean and standard deviation of the control group by which the z-scored outcome variables are standardized.

TABLE G.4. Phone access & task comprehension questions.

	Comparison with active control (AC+INF)					Comparison with pure control (PC)				
	(1) Active Control Group Mean (SD)	(2) Visualization Treatment Effect	(3) Planning Treatment Effect	(4) Column 2 vs. Column 3 *p*-value	(5) *N*	(6) Pure Control Mean (SD)	(7) V+INF Treatment Effect	(8) P+INF Treatment Effect	(9) AC+INF Treatment Effect	(10) *N*
SMS Task Checks										
Participant uses a phone she owns	0.71	0.00	−0.03	0.24	2108	0.70	0.00	−0.03	−0.00	2972
	(0.45)	(0.02)	(0.03)			(0.46)	(0.02)	(0.03)	(0.03)	
Participant uses a phone belonging to her household	0.96	0.01	−0.00	0.30	2108	0.96	0.01	0.00	0.01	2972
	(0.19)	(0.01)	(0.01)			(0.21)	(0.01)	(0.01)	(0.01)	
Proportion for whom accessing a phone for 30mins is very difficult or impossible	0.12	0.02	0.00	0.18	2107	0.13	0.02	−0.00	−0.01	2970
	(0.33)	(0.02)	(0.02)			(0.33)	(0.02)	(0.02)	(0.02)	
Proportion for whom accessing a phone for 1hr is very difficult or impossible	0.17	0.04	0.02	0.55	2107	0.19	0.01	0.00	−0.03	2970
	(0.37)	(0.02)*	(0.02)			(0.39)	(0.02)	(0.02)	(0.02)	
Proportion for whom accessing a phone for 4hrs is very difficult or impossible	0.32	0.02	0.01	0.60	2105	0.35	−0.02	−0.03	−0.04	2967
	(0.46)	(0.03)	(0.03)			(0.48)	(0.03)	(0.03)	(0.03)	
SMS Comprehension questions correct first time	0.81	−0.04	−0.01	0.23	2103	0.72	0.04	0.07	0.07	2955
	(0.39)	(0.03)	(0.03)			(0.45)	(0.03)	(0.02)***	(0.03)***	
Number of attempts at SMS comprehension questions	0.78	0.23	0.12	0.39	2103	1.22	−0.18	−0.27	−0.36	2955
	(1.89)	(0.12)*	(0.12)			(2.36)	(0.13)	(0.13)**	(0.13)***	

Notes: OLS estimates of responses to questions asked as validation checks for the SMS effort task. For each variable, columns (1) and (6) report the mean and standard deviation of the control group. Columns (2)-(3) and (7)-(8) report the coefficients of interest and standard errors in parentheses. All columns include village-level fixed effects and a vector of individual characteristics, and cluster standard errors at the level of the intervention cohort. * denotes significance at 10 pct., ** at 5 pct., and *** at 1 pct. level.

Appendix H: Robustness Checks

TABLE H.1. Randomized experimenter demand treatments (de Quidt, Haushofer, and Roth 2018).

	$\alpha^{+}(\zeta)$ Mean (SD)	$\alpha^{-}(\zeta)$ Mean (SD)	Test: $\alpha^{-}(\zeta)=\alpha^{+}(\zeta)$ p-value
Visualization	2.338	2.264	0.533
	(1.769)	(1.697)	
Planning	2.269	2.146	0.381
	(2.376)	(1.650)	
Active Control	2.085	2.296	0.079*
	(1.745)	(1.734)	
Pure Control	2.163	2.172	0.945
	(1.644)	(1.597)	
All Treatment Groups Combined	2.217	2.223	0.923
	(1.924)	(1.674)	
Observations	1556	1616	3172

Notes: The table shows results from randomized experimenter demand treatments (de Quidt et al. 2018), which were included in the long-run follow-up survey. Respondents are assigned to a group A (B), and told "We hypothesize that people who participated in this study and received the same treatment as you will give higher (lower) responses to these questions than others." They are then asked how often they added chlorine to water collected from their primary source in the last 7 days. Following de Quidt et al. (2018), the responses can be used to obtain bounds $a^{+}(\zeta)$ and $a^{-}(\zeta)$ for the impact of experimenter demand effects on self-reports. The table shows means and standard deviations for $a^{+}(\zeta)$ and $a^{-}(\zeta)$ in all treatment groups, as well as p-values from a test of equality $a^{-}(\zeta)=a^{+}(\zeta)$.

TABLE H.2. Robustness of chlorination effects to within-village testing order.

	Comparison with active control (AC+INF)			Comparison with pure control (PC)		
	(1) Chlorine present (TCR)	(2) Chlorine present (TCR)	(3) Chlorine present (TCR)	(4) Chlorine present (TCR)	(5) Chlorine present (TCR)	(6) Chlorine present (TCR)
Visualization	0.05<br>(0.02)**	0.05<br>(0.02)**	0.01<br>(0.04)	0.06<br>(0.02)***	0.06<br>(0.02)***	0.02<br>(0.04)
Planning	0.02<br>(0.02)	0.01<br>(0.02)	0.02<br>(0.05)	0.04<br>(0.02)*	0.03<br>(0.02)	0.02<br>(0.04)
Active Control				0.03<br>(0.02)	0.02<br>(0.02)	0.04<br>(0.04)
Days after first day of testing within village		−0.00<br>(0.00)			-0.01<br>(0.00)	
Dummy: test done on first day of testing within village			0.00<br>(0.04)			0.00<br>(0.04)
V x with first day of testing			0.06<br>(0.05)			0.07<br>(0.05)
P x first day of testing			0.01<br>(0.05)			0.03<br>(0.05)
AC x with first day of testing						−0.02<br>(0.05)
V x with days after first test		−0.00<br>(0.00)			0.00<br>(0.00)	
P x with days after first test		0.00<br>(0.01)			0.00<br>(0.01)	
AC x with days after first test					0.01<br>(0.01)	
Observations	2012	2012	2012	2839	2839	2839

Notes: OLS estimates of treatment effects on chlorine in water (TCR) after 12 weeks, with additional controls for testing order within the village. For each variable, we report the coefficients of interest, and standard errors in parentheses. * denotes significance at 10 pct., ** at 5 pct., and *** at 1 pct. level. All columns include village-level fixed effects, a vector of individual characteristics, and standard errors which are clustered at the level of the intervention cohort. * denotes significance at 10 pct., ** at 5 pct., and *** at 1 pct. level.

TABLE H.3. Behavioral outcomes (without survey date fixed effects).

		Endline (10-12 weeks)					Follow-Up (30-36 months)				
	MHT Level	(1)<br>Active Control Group Mean (SD)	(2)<br>Visualization Treatment Effect	(3)<br>Planning Treatment Effect	(4)<br>Col. 2 vs. Col. 3 p-value	(5)<br>N	(6)<br>Active Control Group Mean (SD)	(7)<br>Visualization Treatment Effect	(8)<br>Planning Treatment Effect	(9)<br>Col. 2 vs. Col. 3 p-value	(10)<br>N
HEATH OUTCOMES											
Objective measure: chlorine present in water (TCR)	1/-	0.23<br>(0.42)	0.05<br>(0.02)**	0.02<br>(0.02)	0.15	2012					
Objective measure: chlorine sufficient to be safe (FCR)	3/-	0.21<br>(0.40)	0.04<br>(0.02)**<br>[0.04]**	0.01<br>(0.02)<br>[1.00]	0.16	2012					
Main Treatment; Chlorine (self-report)	np/1	0.73<br>(0.45)	0.07<br>(0.02)***<br>[0.00]***	−0.00<br>(0.02)<br>[1.00]	0.00***	2116	0.85<br>(0.35)	0.05<br>(0.02)***<br>[0.03]**	0.02<br>(0.02)<br>[0.38]	0.15	2073
Main Treatment: Boil (self-report)	np/np	0.35<br>(0.48)	0.07<br>(0.03)***<br>[0.02]**	0.05<br>(0.03)*<br>[0.38]	0.45	2116	0.63<br>(0.48)	0.03<br>(0.03)<br>[1.00]	−0.01<br>(0.03)<br>[1.00]	0.21	2073
Diarrhea incidences per child u15, last 3 months	3/1	0.26<br>(0.69)	−0.11<br>(0.03)***<br>[0.00]***	−0.07<br>(0.03)*<br>[0.38]	0.12	2004	0.27<br>(0.73)	−0.01<br>(0.04)<br>[0.27]	−0.05<br>(0.03)<br>[0.38]	0.27	2045
Diarrhea incidences per child u5, last 3 months	np/3	0.34<br>(0.86)	−0.16<br>(0.05)***<br>[0.00]***	−0.06<br>(0.05)<br>[0.76]	0.03**	1682	0.39<br>(1.04)	0.01<br>(0.06)<br>[1.00]	−0.02<br>(0.06)<br>[1.00]	0.73	1612
Proportion of children taken for healthcare check-up	3/3	0.21<br>(0.34)	−0.04<br>(0.02)**<br>[0.03]**	−0.02<br>(0.02)<br>[0.76]	0.41	1995	0.36<br>(0.37)	−0.01<br>(0.02)<br>[1.00]	−0.01<br>(0.02)<br>[1.00]	0.90	2045
Proportion of children u15 vaccinated, last 3 months	3/-	0.22<br>(0.35)	0.00<br>(0.02)<br>[0.35]	−0.01<br>(0.02)<br>[1.00]	0.35	1990					
Number of ANC visits, last 3 months (if pregnant)	3/-	1.26<br>(1.19)	−0.24<br>(0.49)<br>[0.26]	0.06<br>(0.35)<br>[1.00]	0.44	200					
SAVINGS OUTCOMES											
Amount saved regularly (per week, KES)	2/2	93.96<br>(230.26)	24.89<br>(12.37)**<br>[0.16]	3.28<br>(12.51)<br>[1.00]	0.10	2108	407.50<br>(605.28)	60.00<br>(33.65)*<br>[0.10]*	26.67<br>(33.16)<br>[0.51]	0.34	2073
Indicator: Amount saved regularly is positive	3/3	0.36<br>(0.48)	0.13<br>(0.03)***<br>[0.00]***	−0.02<br>(0.03)<br>[1.00]	0.00***	2108	0.78<br>(0.42)	0.05<br>(0.02)**<br>[0.14]	0.02<br>(0.02)<br>[1.00]	0.13	2073
Number of ROSCAs [joined in last 3 months/total]	3/3	0.17<br>(0.44)	0.04<br>(0.03)*<br>[0.06]*	0.01<br>(0.02)<br>[1.00]	0.17	2108	1.08<br>(1.05)	0.13<br>(0.06)**<br>[0.14]	0.10<br>(0.06)*<br>[1.00]	0.59	2073
Weekly ROSCA savings	np/np	205.93<br>(304.72)	34.67<br>(15.81)**<br>[0.03]**	10.83<br>(16.02)<br>[1.00]	0.12	2108	246.07<br>(363.71)	45.90<br>(22.36)*<br>[0.10]	33.84<br>(21.24)<br>[0.42]	0.60	2073
Indicator: Saves for productive investments	3/3	0.17<br>(0.38)	0.11<br>(0.02)***<br>[0.00]***	−0.01<br>(0.02)<br>[1.00]	0.00***	2108	0.62<br>(0.49)	0.02<br>(0.02)<br>[1.00]	0.01<br>(0.03)<br>[1.00]	0.74	2073
Total Savings Balance (KES)	–/3						2542.85<br>(6378.76)	1046.08<br>(423.20)**<br>[0.14]	45.72<br>(401.32)<br>[1.00]	0.01***	2073
LABOR OUTCOMES											
Total hours of work [last 3 months/last 7 days]	2/2	106.11<br>(174.61)	−6.78<br>(9.56)<br>[0.90]	−24.33<br>(9.03)***<br>[0.02]**	0.06*	2108	13.25<br>(15.41)	2.42<br>(0.87)***<br>[0.05]**	0.91<br>(0.87)<br>[0.42]	0.09*	2073
Total days of work, last 3 months	3/-	21.22<br>(30.09)	−0.59<br>(1.64)<br>[0.32]	−3.91<br>(1.60**<br>[0.27]	0.04**	2108					
Earnings, cash and in-kind [monthly/last 7 days]	3/3	1094.50<br>(2865.35)	3.11<br>(147.23)<br>[0.39]	−1.23<br>(163.19)<br>[1.00]	0.97	2108	677.26<br>(1343.83)	108.14<br>(77.42)<br>[0.81]	95.12<br>(79.96)<br>[1.00]	0.87	2073
OTHER BEHAVIORAL OUTCOMES											
Index of investment in children's education (z-score)	2/-	0.00<br>(1.00)	−0.02<br>(0.06)<br>[0.90]	0.01<br>(0.07)<br>[1.00]	0.60	1420					

Notes: OLS estimates of treatment effects, using the specification from the PAP, without survey date fixed effects. For each variable, columns (1) and (6) report the mean and standard deviation of the control group. Columns (2)-(3) and (7)-(8) report the coefficients of interest and standard errors in parentheses. * denotes significance at 10 pct., ** at 5 pct., and *** at 1 pct. level. Square brackets contain additional p-values corrected for multiple hypothesis testing using the false discovery rate. All columns include village-level fixed effects, a vector of individual characteristics, and standard errors which are clustered at the level of the intervention cohort. The sample in all regressions is restricted to participants in active treatment groups who attended the baseline survey. Where available, we control for the baseline outcome of the dependent variable. Outcome measures are listed on the left, and are described in detail in Section 4.

TABLE H.4. Psychological outcomes (without survey date fixed effects).

		Endline (10-12 weeks)					Follow-Up (30-36 months)				
	MHT Level	(1)<br>Active Control Group Mean (SD)	(2)<br>Visualization Treatment Effect	(3)<br>Planning Treatment Effect	(4)<br>Column 2 vs. Column 3 p-value	(5)<br>N	(6)<br>Active Control Group Mean (SD)	(7)<br>Visualization Treatment Effect	(8)<br>Planning Treatment Effect	(9)<br>Column 2 vs. Column 3 p-value	(10)<br>N
PLANNING SKILLS											
BADS score (z-score)	1/-	0.00<br>(1.00)	–0.01<br>(0.05)<br>[0.83]	0.04<br>(0.05)<br>[0.91]	0.31	2103					
Tower of London (z-score)	2/-	0.00<br>(1.00)	0.01<br>(0.05)<br>[0.79]	–0.04<br>(0.05)<br>[0.50]	0.28	2103					
TIME PREFERENCES											
β^{Effort}	1/-	0.982<br>(0.005)	0.007<br>(0.006)<br>[0.83]	0.005<br>(0.007)<br>[0.91]	0.33	2068					
δ^{Effort}	2/-	0.999<br>(0.001)	–0.001<br>(0.001)<br>[0.26]	–0.002<br>(0.001)**<br>[0.06]*	0.16	2068					
Utility Forecasting: Vividness (z-score)	–/1						0.00<br>(1.00)	0.11<br>(0.05)**<br>[0.06]**	–0.03<br>(0.06)<br>[0.71]	0.01***	2073
Utility Forecasting: Practice (z-score)	–/2						–0.00<br>(1.00)	0.10<br>(0.05)*<br>[0.15]	0.11<br>(0.05)**<br>[0.13]	0.77	2073
β^{MPL}	1/-	1.05<br>(0.46)	–0.02<br>(0.02)<br>[0.41]	0.02<br>(0.03)<br>[0.53]	0.21	2103					
δ^{MPL}	2/-	0.98<br>(0.02)	–0.00<br>(0.00)<br>[0.36]	–0.00<br>(0.00)<br>[0.31]	0.84	2103					
Time Preferences, qualitative (z-score)	–/2						–0.00<br>(1.00)	0.10<br>(0.05)*<br>[0.15]	–0.06<br>(0.06)<br>[0.36]	0.00***	2073
One-year discount factor (GPS staircase)	–/2						0.52<br>(0.13)	0.00<br>(0.01)<br>[0.93]	–0.01<br>(0.01)<br>[0.36]	0.17	2073
SELF-EFFICACY											
General Self-Efficacy Score (GSE) (z-score)	2/1	0.00<br>(1.00)	0.16<br>(0.05)***<br>[0.00]***	0.12<br>(0.05)**<br>[0.06]*	0.38	2103	–0.00<br>(1.00)	0.07<br>(0.05)<br>[0.08]*	–0.07<br>(0.05)<br>[0.71]	0.00***	2073

Notes: OLS estimates of treatment effects, using the specification from the PAP, without survey date fixed effects. For each variable, columns (1) and (6) report the mean and standard deviation of the control group. Columns (2)-(3) and (7)-(8) report the coefficients of interest and standard errors in parentheses. * denotes significance at 10 pct., ** at 5 pct., and *** at 1 pct. level. Square brackets contain additional p-values corrected for multiple hypothesis testing using the false discovery rate. All columns include village-level fixed effects, a vector of individual characteristics, and standard errors which are clustered at the level of the intervention cohort. The sample in all regressions is restricted to participants in active treatment groups who attended the baseline survey. Where available, we control for the baseline outcome of the dependent variable. Outcome measures are listed on the left, and are described in detail in Section 4.

References

Andreoni, James and Charles Sprenger (2012). “Estimating Time Preferences from Convex Budgets.” *American Economic Review*, 102(7), 3333–3356.

Augenblick, Ned (2017). “Short-Term Time Discounting of Unpleasant Tasks.” *Working Paper, UC Berkeley*.

Augenblick, Ned, Muriel Niederle, and Charles Sprenger (2015). “Working Over Time: Dynamic Inconsistency in Real Effort Tasks.” *The Quarterly Journal of Economics*, 130(3), 1067–1115.

Charness, Gary, Uri Gneezy, and Alex Imas (2013). “Experimental Methods: Eliciting Risk Preferences.” *Journal of Economic Behavior & Organization*, 87, 43–51.

de Quidt, Jonathan, Johannes Haushofer, and Christopher Roth (2018). “Measuring and Bounding Experimenter Demand.” *American Economic Review*, 108(11), 3266–3302.

DellaVigna, Stefano and Devin Pope (2017). “What Motivates Effort? Evidence and Expert Forecasts.” *The Review of Economic Studies*, 85(2), 1029–1069.

Kremer, Michael, Edward Miguel, Sendhil Mullainathan, Clair Null, and Alix Peterson Zwane (2011). “Social Engineering: Evidence from a Suite of Take-Up Experiments in Kenya.” *Working Paper, Harvard University*.

Laibson, David (1997). “Golden Eggs and Hyperbolic Discounting.” *The Quarterly Journal of Economics*, 112(2), 443–478.

Null, Clair, Christine Stewart, Amy Pickering, Holly Dentz, Benjamin Arnold, Charles Arnold, Jade Benjamin-Chung, Thomas Clasen, Kathryn Dewey, Lia Fernald, et al. (2018). “Effects of Water Quality, Sanitation, Handwashing, and Nutritional Interventions on Diarrhoea and Child Growth in Rural Kenya: A Cluster-Randomised Controlled Trial.” *The Lancet Global Health*, 6(3), e316–e329.

Troeger, Christopher, Danny Colombara, Puja Rao, Ibrahim Khalil, Alexandria Brown, Thomas Brewer, Richard Guerrant, Eric Houpt, Karen Kotloff, Kavita Misra, William Petri, James Platts-Mills, Mark Riddle, Scott Swartz, Mohammad Forouzanfar, Robert Reiner, Simon Hay, and Ali Mokdad (2018). “Global Disability-Adjusted Life-Year Estimates of Long-Term Health Burden and Undernutrition Attributable to Diarrhoeal Diseases in Children Younger than 5 Years.” *The Lancet Global Health*, 6(3), e255–e269.

Wisniewski, Janna, Angela Acosta, Jan Kolaczinski, Hannah Koenker, and Joshua Yukich (2020). “Systematic Review and Meta-analysis of the Cost and Cost-effectiveness of Distributing Insecticide-treated Nets for the Prevention of Malaria.” *Acta Tropica*, 202, 105229.
