# Supplementary material for: Can Simple Psychological Interventions Increase Preventive Health Investment?
Source: J Eur Econ Assoc. 2021 Nov 30;20(3):1001–47. doi: 10.1093/jeea/jvab052 (PMC9194950; doi:10.1093/jeea/jvab052)
Supplement: jvab052_John_Orkin_Reproduction [file jvab052_john_orkin_reproduction.zip › Reproduction/Data/SMS effort files/MLE_Nov2018_PC.docx]

Number of obs = 39,428

Wald chi2(0) = .

Log pseudolikelihood = -163963.63 Prob > chi2 = .

(Std. Err. adjusted for 2,906 clusters in subject_id)

------------------------------------------------------------------------------

| Robust | Coef. Std. Err. z P>|z| [95% Conf. Interval]

-------------+----------------------------------------------------------------

sigma | _cons | 15.48109 .1191969 129.88 0.000 15.24747 15.71472

-------------+----------------------------------------------------------------

delta | _cons | .9951738 .0023606 421.57 0.000 .9905471 .9998006

-------------+----------------------------------------------------------------

phi | _cons | .0428986 .0269125 1.59 0.111 -.0098489 .0956461

-------------+----------------------------------------------------------------

gamma | _cons | 1.753181 .2132964 8.22 0.000 1.335127 2.171234

-------------+----------------------------------------------------------------

beta | _cons | .9528473 .0197765 48.18 0.000 .914086 .9916086

-------------+----------------------------------------------------------------

s | _cons | 7.756013 4.573181 1.70 0.090 -1.207256 16.71928

-------------+----------------------------------------------------------------

b_TE_ITF | _cons | .0071959 .0184329 0.39 0.696 -.0289318 .0433237

-------------+----------------------------------------------------------------

b_TE_BA | _cons | .0121393 .0175829 0.69 0.490 -.0223226 .0466012

-------------+----------------------------------------------------------------

b_TE_PLA | _cons | .0094874 .0179645 0.53 0.597 -.0257225 .0446973

-------------+----------------------------------------------------------------

d_TE_ITF | _cons | -.0006477 .0023244 -0.28 0.781 -.0052036 .0039081

-------------+----------------------------------------------------------------

d_TE_BA | _cons | -.0020803 .00226 -0.92 0.357 -.0065098 .0023491

-------------+----------------------------------------------------------------

d_TE_PLA | _cons | .0031804 .0024031 1.32 0.186 -.0015295 .0078903

-------------+----------------------------------------------------------------

s_TE_ITF | _cons | 1.848624 .8939351 2.07 0.039 .0965432 3.600705

-------------+----------------------------------------------------------------

s_TE_BA | _cons | .6319981 .5841385 1.08 0.279 -.5128923 1.776889

-------------+----------------------------------------------------------------

s_TE_PLA | _cons | .6016039 .5419265 1.11 0.267 -.4605526 1.66376

-------------+----------------------------------------------------------------

s_zero | _cons | -3.610802 3.147761 -1.15 0.251 -9.780302 2.558697

-------------+----------------------------------------------------------------

g_TE_ITF | _cons | .0572593 .0411201 1.39 0.164 -.0233346 .1378531

-------------+----------------------------------------------------------------

g_TE_BA | _cons | .0010119 .0359725 0.03 0.978 -.0694929 .0715167

-------------+----------------------------------------------------------------

g_TE_PLA | _cons | -.0004286 .0357315 -0.01 0.990 -.0704611 .0696038

-------------+----------------------------------------------------------------

g_zero | _cons | -.2061236 .0835744 -2.47 0.014 -.3699263 -.0423208

-------------+----------------------------------------------------------------

d1 | _cons | -.2318869 .160799 -1.44 0.149 -.5470472 .0832735

-------------+----------------------------------------------------------------

d2 | _cons | -.0751658 .1512125 -0.50 0.619 -.371537 .2212053

-------------+----------------------------------------------------------------

d3 | _cons | -.1931811 .2319439 -0.83 0.405 -.6477827 .2614206

-------------+----------------------------------------------------------------

d4 | _cons | -.2585335 .2696657 -0.96 0.338 -.7870686 .2700015

-------------+----------------------------------------------------------------

d5 | _cons | -.2789058 .2474977 -1.13 0.260 -.7639925 .2061808

-------------+----------------------------------------------------------------

d6 | _cons | .0022709 .0995482 0.02 0.982 -.19284 .1973817

------------------------------------------------------------------------------

Warning: convergence not achieved
