# Supplementary material for: Can Simple Psychological Interventions Increase Preventive Health Investment?
Source: J Eur Econ Assoc. 2021 Nov 30;20(3):1001–47. doi: 10.1093/jeea/jvab052 (PMC9194950; doi:10.1093/jeea/jvab052)
Supplement: jvab052_John_Orkin_Reproduction [file jvab052_john_orkin_reproduction.zip › Reproduction/Data/SMS effort files/MLE_Nov2018_PC_compliers.docx]

NB: Estimates from 100 iterations, but all iterations “backed up” after iteration 46.

Number of obs = 39,428

Wald chi2(0) = .

Log pseudolikelihood = -163948.65 Prob > chi2 = .

(Std. Err. adjusted for 2,906 clusters in subject_id)

------------------------------------------------------------------------------

| Robust | Coef. Std. Err. z P>|z| [95% Conf. Interval]

-------------+----------------------------------------------------------------

sigma | _cons | 15.47522 .119174 129.85 0.000 15.24164 15.70879

-------------+----------------------------------------------------------------

delta | _cons | .9948689 .002456 405.08 0.000 .9900552 .9996825

-------------+----------------------------------------------------------------

phi | _cons | .0313269 .0275375 1.14 0.255 -.0226457 .0852995

-------------+----------------------------------------------------------------

gamma | _cons | 1.67195 .2625218 6.37 0.000 1.157417 2.186483

-------------+----------------------------------------------------------------

beta | _cons | .9644588 .0173329 55.64 0.000 .930487 .9984306

-------------+----------------------------------------------------------------

s | _cons | 6.470693 4.767128 1.36 0.175 -2.872705 15.81409

-------------+----------------------------------------------------------------

b_TE_ITF | _cons | .0025458 .0145035 0.18 0.861 -.0258805 .0309721

-------------+----------------------------------------------------------------

b_TE_BA | _cons | .0052628 .013708 0.38 0.701 -.0216044 .03213

-------------+----------------------------------------------------------------

b_TE_PLA | _cons | -.0037972 .0133264 -0.28 0.776 -.0299166 .0223221

-------------+----------------------------------------------------------------

d_TE_ITF | _cons | -.0001562 .0018619 -0.08 0.933 -.0038054 .0034931

-------------+----------------------------------------------------------------

d_TE_BA | _cons | 3.32e-06 .0017227 0.00 0.998 -.003373 .0033797

-------------+----------------------------------------------------------------

d_TE_PLA | _cons | .00404 .0023562 1.71 0.086 -.0005781 .0086582

-------------+----------------------------------------------------------------

s_TE_ITF | _cons | 1.806519 1.200214 1.51 0.132 -.5458568 4.158894

-------------+----------------------------------------------------------------

s_TE_BA | _cons | .8145967 .6171208 1.32 0.187 -.394938 2.024131

-------------+----------------------------------------------------------------

s_TE_PLA | _cons | .7547337 .5452268 1.38 0.166 -.3138912 1.823359

-------------+----------------------------------------------------------------

s_zero | _cons | -3.098314 3.199127 -0.97 0.333 -9.368489 3.17186

-------------+----------------------------------------------------------------

g_TE_ITF | _cons | .0865943 .0357282 2.42 0.015 .0165684 .1566203

-------------+----------------------------------------------------------------

g_TE_BA | _cons | .0305819 .0324772 0.94 0.346 -.0330722 .0942361

-------------+----------------------------------------------------------------

g_TE_PLA | _cons | .0287412 .0296872 0.97 0.333 -.0294447 .086927

-------------+----------------------------------------------------------------

g_zero | _cons | -.2090253 .0884866 -2.36 0.018 -.3824559 -.0355947

-------------+----------------------------------------------------------------

d1 | _cons | -.14168 .1399664 -1.01 0.311 -.416009 .1326491

-------------+----------------------------------------------------------------

d2 | _cons | -.0270827 .1106865 -0.24 0.807 -.2440243 .1898589

-------------+----------------------------------------------------------------

d3 | _cons | -.1272928 .1859145 -0.68 0.494 -.4916784 .2370929

-------------+----------------------------------------------------------------

d4 | _cons | -.1373157 .1976971 -0.69 0.487 -.524795 .2501635

-------------+----------------------------------------------------------------

d5 | _cons | -.1555919 .183765 -0.85 0.397 -.5157648 .2045809

-------------+----------------------------------------------------------------

d6 | _cons | .0159494 .073722 0.22 0.829 -.128543 .1604418

------------------------------------------------------------------------------
