# Supplementary material for: Can Simple Psychological Interventions Increase Preventive Health Investment?
Source: J Eur Econ Assoc. 2021 Nov 30;20(3):1001–47. doi: 10.1093/jeea/jvab052 (PMC9194950; doi:10.1093/jeea/jvab052)
Supplement: jvab052_John_Orkin_Reproduction [file jvab052_john_orkin_reproduction.zip › Reproduction/Data/SMS effort files/MLE_Nov2018_placebo.docx]

Number of obs = 28,304

Wald chi2(0) = .

Log pseudolikelihood = -118075.75 Prob > chi2 = .

(Std. Err. adjusted for 2,068 clusters in subject_id)

------------------------------------------------------------------------------

| Robust | Coef. Std. Err. z P>|z| [95% Conf. Interval]

-------------+----------------------------------------------------------------

sigma | _cons | 15.68586 .1395974 112.36 0.000 15.41225 15.95946

-------------+----------------------------------------------------------------

delta | _cons | .9991141 .0005085 1964.76 0.000 .9981174 1.000111

-------------+----------------------------------------------------------------

phi | _cons | .0064123 .0007923 8.09 0.000 .0048594 .0079652

-------------+----------------------------------------------------------------

gamma | _cons | 1.268639 .03236 39.20 0.000 1.205215 1.332063

-------------+----------------------------------------------------------------

beta | _cons | .9821594 .004686 209.59 0.000 .972975 .9913438

-------------+----------------------------------------------------------------

s | _cons | 2.102163 .1993197 10.55 0.000 1.711504 2.492823

-------------+----------------------------------------------------------------

b_TE_ITF | _cons | .0071415 .0058991 1.21 0.226 -.0044204 .0187035

-------------+----------------------------------------------------------------

b_TE_BA | _cons | .0047079 .0065993 0.71 0.476 -.0082266 .0176424

-------------+----------------------------------------------------------------

d_TE_ITF | _cons | -.0010773 .0006592 -1.63 0.102 -.0023693 .0002147

-------------+----------------------------------------------------------------

d_TE_BA | _cons | -.0017516 .0007346 -2.38 0.017 -.0031915 -.0003118

-------------+----------------------------------------------------------------

s_TE_ITF | _cons | -.4167688 .2450254 -1.70 0.089 -.8970097 .0634721

-------------+----------------------------------------------------------------

s_TE_BA | _cons | -.0538502 .2913456 -0.18 0.853 -.624877 .5171766

-------------+----------------------------------------------------------------

s_zero | _cons | .2023294 .1862435 1.09 0.277 -.1627011 .5673599

-------------+----------------------------------------------------------------

g_TE_ITF | _cons | -.0706259 .0410551 -1.72 0.085 -.1510923 .0098405

-------------+----------------------------------------------------------------

g_TE_BA | _cons | -.0092871 .0452153 -0.21 0.837 -.0979075 .0793334

-------------+----------------------------------------------------------------

g_zero | _cons | .0154087 .0297195 0.52 0.604 -.0428404 .0736579

-------------+----------------------------------------------------------------

d1 | _cons | -.0390088 .0147308 -2.65 0.008 -.0678806 -.010137

-------------+----------------------------------------------------------------

d2 | _cons | -.0187345 .0222063 -0.84 0.399 -.0622581 .0247891

-------------+----------------------------------------------------------------

d3 | _cons | -.0062616 .0230381 -0.27 0.786 -.0514155 .0388924

-------------+----------------------------------------------------------------

d4 | _cons | -.0204673 .0204938 -1.00 0.318 -.0606344 .0196997

-------------+----------------------------------------------------------------

d5 | _cons | -.0290169 .0196997 -1.47 0.141 -.0676276 .0095938

-------------+----------------------------------------------------------------

d6 | _cons | .0150864 .0146291 1.03 0.302 -.013586 .0437589
