# Supplementary material for: Can Simple Psychological Interventions Increase Preventive Health Investment?
Source: J Eur Econ Assoc. 2021 Nov 30;20(3):1001–47. doi: 10.1093/jeea/jvab052 (PMC9194950; doi:10.1093/jeea/jvab052)
Supplement: jvab052_John_Orkin_Reproduction [file jvab052_john_orkin_reproduction.zip › Reproduction/README.docx]

Manuscript name: **Can Simple Psychological Interventions Increase Preventative Health Investment?**

Authors: Anett John and Kate Orkin

The main folder contains subfolders each containing the data files and scripts necessary to replicate the analyses in the paper. It also contains master scripts for the paper. The folder contains the following do files:

- *“torun.do”* is the master do file that runs all the tables. Each of these tables runs globals before running analyses. In order to reproduce the results presented in this study, amend line 22 of this do file.
- “*global.do”* runs globals for tables that have both data from the endline and follow up survey.
- *“globals_end.do”* runs globals from the endline and
- “*globals_ps.do”* run globals for the follow up survey

The main folder has the following subfolders:

1. **DATA**

The *Data* folder contains the following:

| SOBC3_Data_Final.dta | This is the main dataset, a merge of data sets from the baseline, endline and follow-up survey rounds |
| --- | --- |
| saliencedata_foranalysis1_long.dta | This is the data from the salience test at endline |
| saliencedata_cleaned | This is the data from the salience test at endline |
| Endline folder | This is the dataset from effort discounting tasks and monetary price list run to elicit time preferences and utility forecasting |
| SMS effort files folder | This is the dataset from effort discounting tasks run to elicit time preferences and utility forecasting |
| Pvals folder |  |

1. **ADO**

This folder contains the extra STATA program files required to carry out the analysis.

1. **ANALYSIS**

The *Analysis* folder contains the do files required to produce the individual tables. Each table do file initially runs a do file that contains all the globals. Specific tables will also run output do files for the endline and follow-up rounds of the survey. The tables are in the subfolder called *output*. The table below lists all the tables in the paper and which main do file runs the table. The Tables column is the name of the outputs (.tex files) from the do files (produced in Tables subfolder in the main folder)

1. **TABLES**

This is the subfolder that contains all the table outputs as .tex files

| **#** | **Label** | **Do File** | **Output (endline)** | **Output**  **(follow-up)** | **Tables** |
| --- | --- | --- | --- | --- | --- |
| 1 | Experimental Integrity | table_1 | table_experimental |  | experimental_ps |
| 2 | Behavioural outcomes | table_2 | table_main_behav_end | table_main_behav_ps | behavoutcomes_end  behavoutcomes_ps |
| 3 | Chlorine-related outcomes in dispenser vs. non-dispenser villages | table_3 | table_wash_end | table_wash_ps | washvillages_ac_end  washvillages_ac_ps |
| 4 | Psychological outcomes | table_4 | table_psych_end | table_psych_enps | pyschoutcomes_end  psychoutcomes_ps |
| 5 | Alternative Mechanisms | table_5 | table_main_behav_end | - | mechanisms_end  saliencetable_AC_el  saliencetable_AC_ps  table_5^[[1]](#footnote-1)^ |
|  |  |  |  |  |  |
| **D. Supplementary evidence on mechanisms** | |  |  |  |  |
| A.1 | Treatment Effect Heterogeneity on Diarrhea by Season and Water Source | table_a1 | - | - | tablea1 |
| A.2 | Correlates of Chlorination | table_a2 | - | - | chlor_mechanisms_ed |
| A.3 | Correlates of Savings | table_a3 | - | - | Saving_mechanisms_ed |
| A.4 | Correlates of Chlorination and Savings – Alternative Mechanisms | table_a4 | - | - | chlor_sav_mechanisms_ed |
|  |  |  |  |  |  |
| **E. Pure Control Comparison** | |  |  |  |  |
| A.5 | Behavioral outcomes (comparison with pure control group | table_a5 | table_main_behav_end_pc | table_main_behav_ps_pc | behavoutcomes_pc  behavoutcomes_ps_pc |
| A.6 | Psychological outcomes (comparison with pure control group) | table_a6 | table_psych_end_pc | table_psych_ps_pc | psychoutcomes_end_pc  psychoutcomes_ps_pc |
| A.7 | Alternative Mechanisms (comparison with pure control group) | table_a7 | table_main_behav_end_pc | - | mechanisms_pc  saliencetable2_el  saliencetable2 |
|  |  |  |  |  |  |
| **F. Test corrections and experimental integrity** | |  |  |  |  |
| A.10 | Baseline balance: main outcomes | table_a10 | table_balance | - | baselinebalancecheck |
| A.11 | Baseline balance: dispenser vs. non-dispenser villages | table_a11 | - | - | table_a11 |
| A.12 | Attrition analysis: treatments vs. active control | table_a12 | - | - | attritionanalysis_pla |
| A.13 | Attrition analysis: active treatments vs. pure control | table_a13 | - | - | attritionanalysis_pc |
|  |  |  |  |  |  |
| **G. Detailed descriptions of outcome measures** | |  |  |  |  |
| A.16 | Raw means of z-scored outcome variables | table_a16 | - | - | table_a16b |
| A.17 | Phone access & task comprehension questions | table_a17 | table_main_behav_end | - | phoneaccess |
|  |  |  |  |  |  |
| **H. Robustness checks** | |  |  |  |  |
| A.18 | Randomized Experimenter Demand Treatments (de Quidt, Haushofer, and Roth 2018) | table_a18 | - | - | table_a18 |
| A.19 | Robustness of chlorination effects to within-village testing order | table_a19 | - | - | robustness_chlorine |
| A.20 | Behavioral outcomes (without survey date fixed effects) | table_a20 | table_main_behav_end_nofe | table_main_behav_ps_nofe | behavoutcomes_nofe  behvoutcomes_ps_nofe |
| A.21 | Psychological outcomes (without survey date fixed effects) | table_a21 | table_psych_end_nofe | table_psych_ps_nofe | psychoutcomes_end_nofe  psychoutcomes_ps_nofe |

1. table_5.tex is a log file of the table_5.do. This is included as it contains p-values which are part of the Salience table output in the paper but not formatted into the table outputs saliencetable_AC_el and saliencetable_AC_ps. [↑](#footnote-ref-1)
