# Supplementary figures and images for: Can Simple Psychological Interventions Increase Preventive Health Investment?
Source: J Eur Econ Assoc. 2021 Nov 30;20(3):1001–47. doi: 10.1093/jeea/jvab052 (PMC9194950; doi:10.1093/jeea/jvab052)

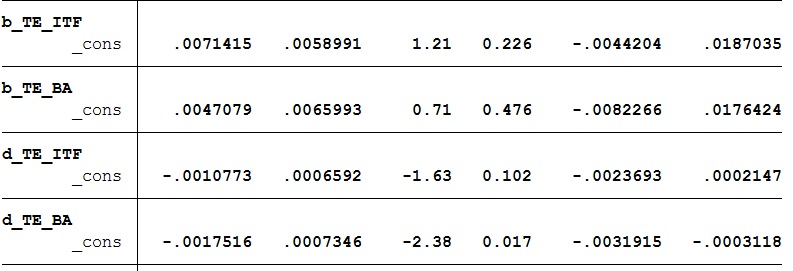

Supplement: jvab052_John_Orkin_Reproduction [file jvab052_john_orkin_reproduction.zip › Reproduction/Data/SMS effort files/MLE_estimation_Nov2018_placebo.jpg]

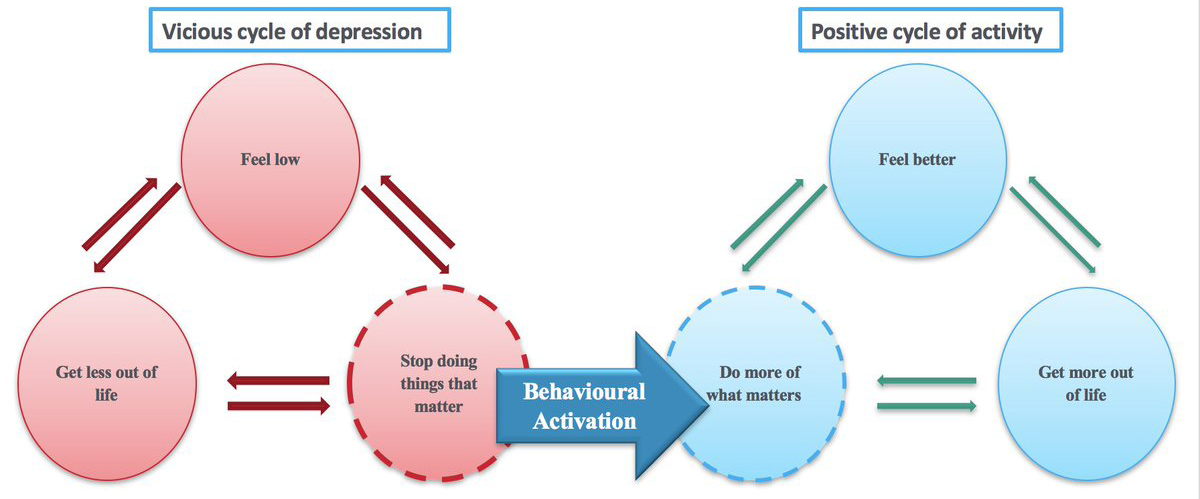

Supplement: jvab052_John_Orkin_TeachingMaterial [file jvab052_john_orkin_teachingmaterial.zip › Figures/BA_cycle.jpg]

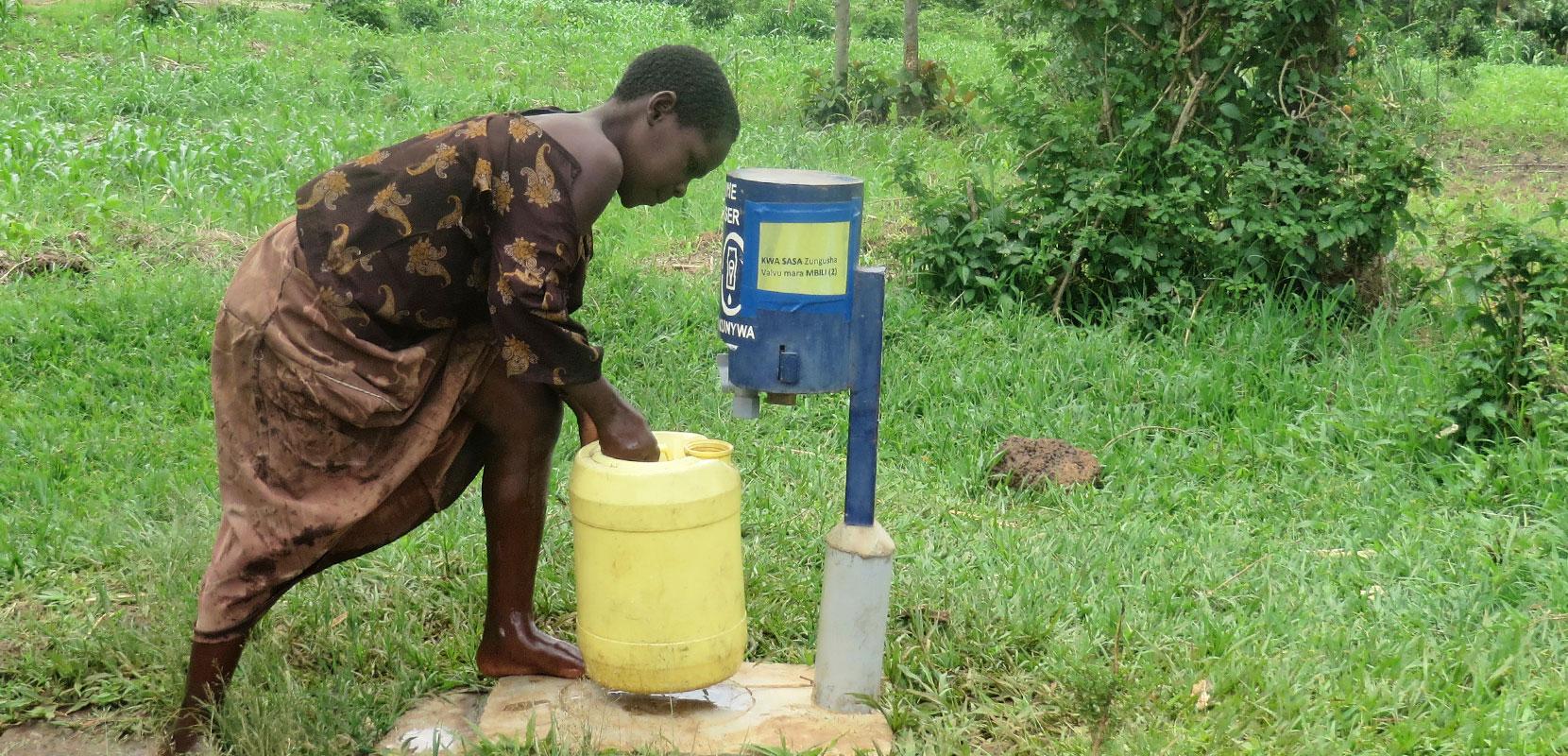

Supplement: jvab052_John_Orkin_TeachingMaterial [file jvab052_john_orkin_teachingmaterial.zip › Figures/chlorine-dispensers-girl.jpg]

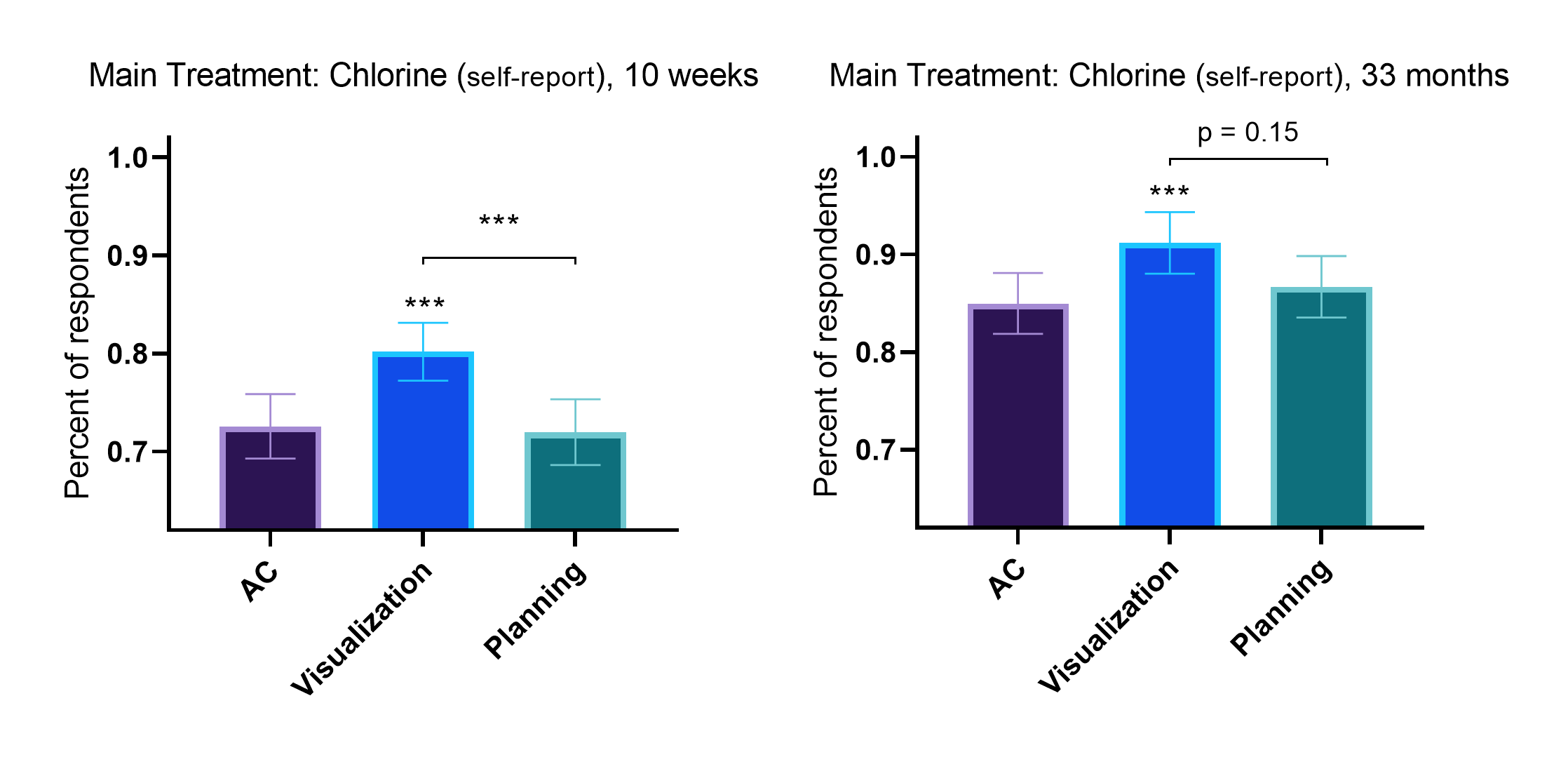

Supplement: jvab052_John_Orkin_TeachingMaterial [file jvab052_john_orkin_teachingmaterial.zip › Figures/Chlorine_selfreport.png]

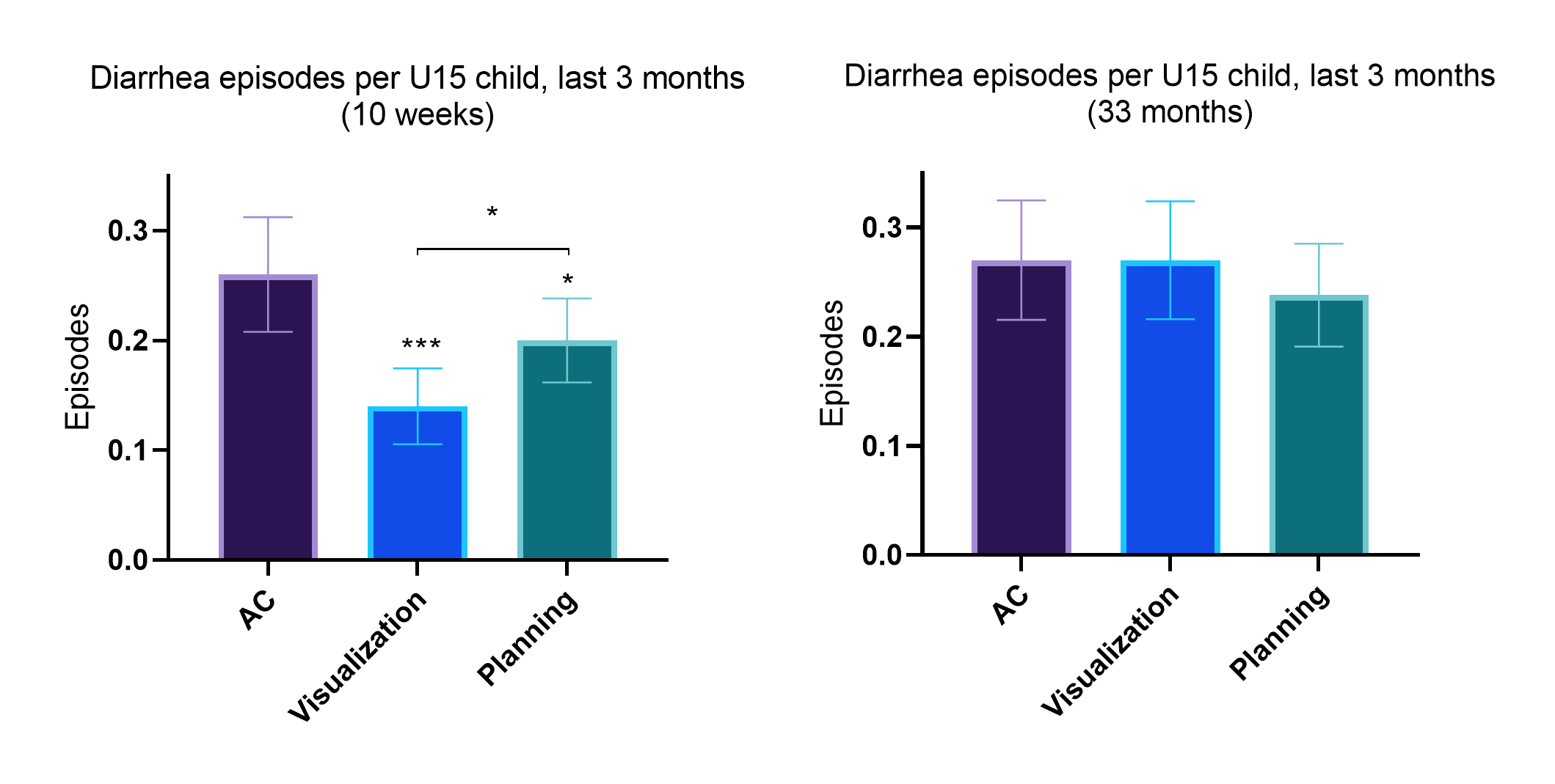

Supplement: jvab052_John_Orkin_TeachingMaterial [file jvab052_john_orkin_teachingmaterial.zip › Figures/Diarrhea_u15.png]

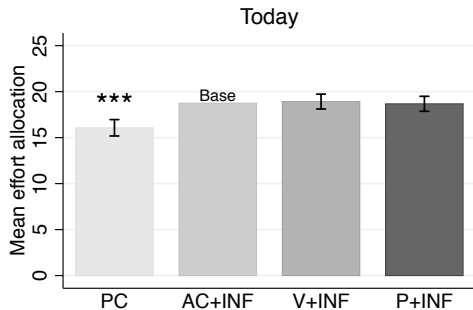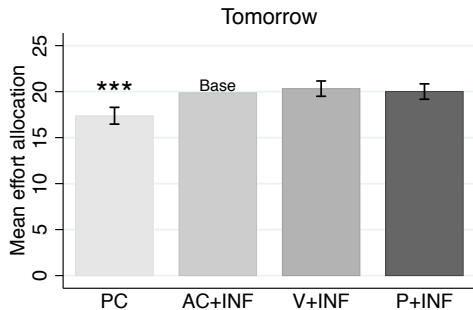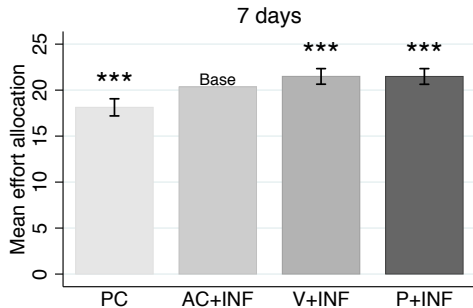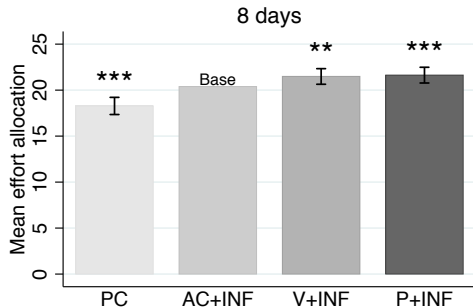

\* denotes significance at 10 pct., \*\* at 5 pct., \*\*\* at 1 pct. level

Supplement: jvab052_John_Orkin_TeachingMaterial [file jvab052_john_orkin_teachingmaterial.zip › Figures/effort_response.pdf]

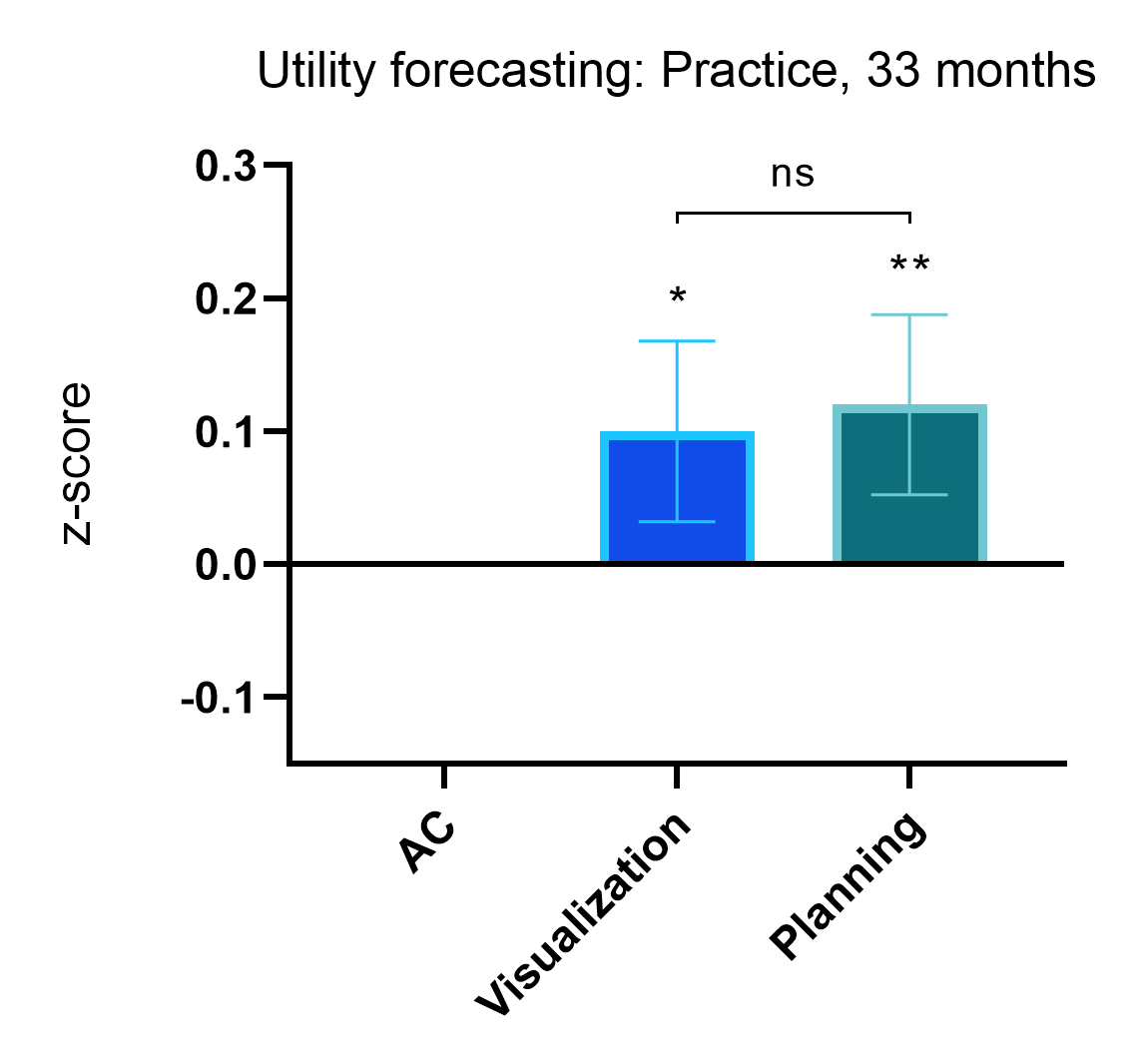

Supplement: jvab052_John_Orkin_TeachingMaterial [file jvab052_john_orkin_teachingmaterial.zip › Figures/forecasting_practice_LR.png]

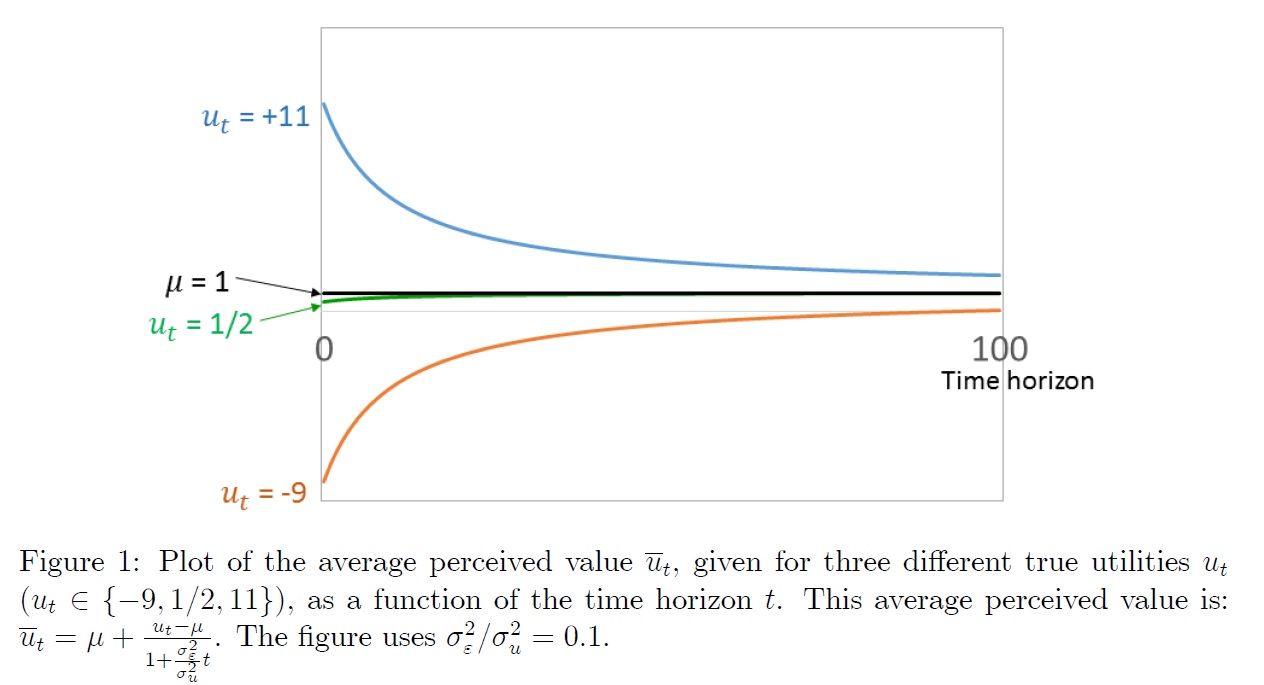

Supplement: jvab052_John_Orkin_TeachingMaterial [file jvab052_john_orkin_teachingmaterial.zip › Figures/Gabaix_Laibson.jpg]

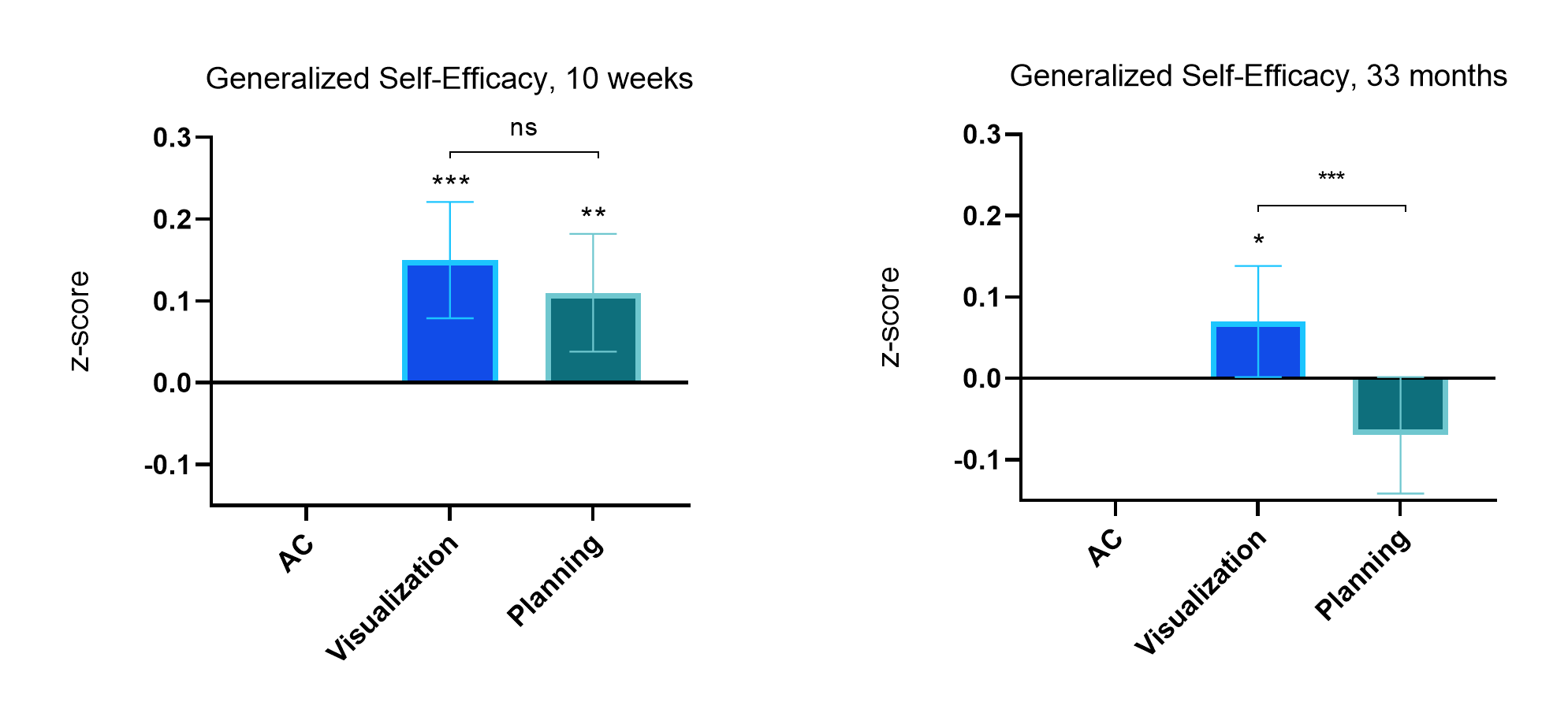

Supplement: jvab052_John_Orkin_TeachingMaterial [file jvab052_john_orkin_teachingmaterial.zip › Figures/GSE.png]

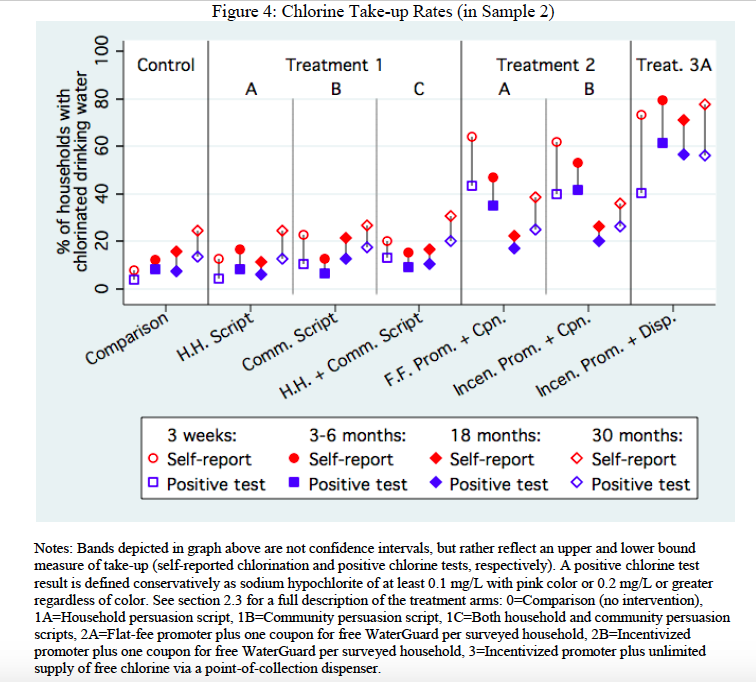

Supplement: jvab052_John_Orkin_TeachingMaterial [file jvab052_john_orkin_teachingmaterial.zip › Figures/Kremer_Dispenser_Figure.png]

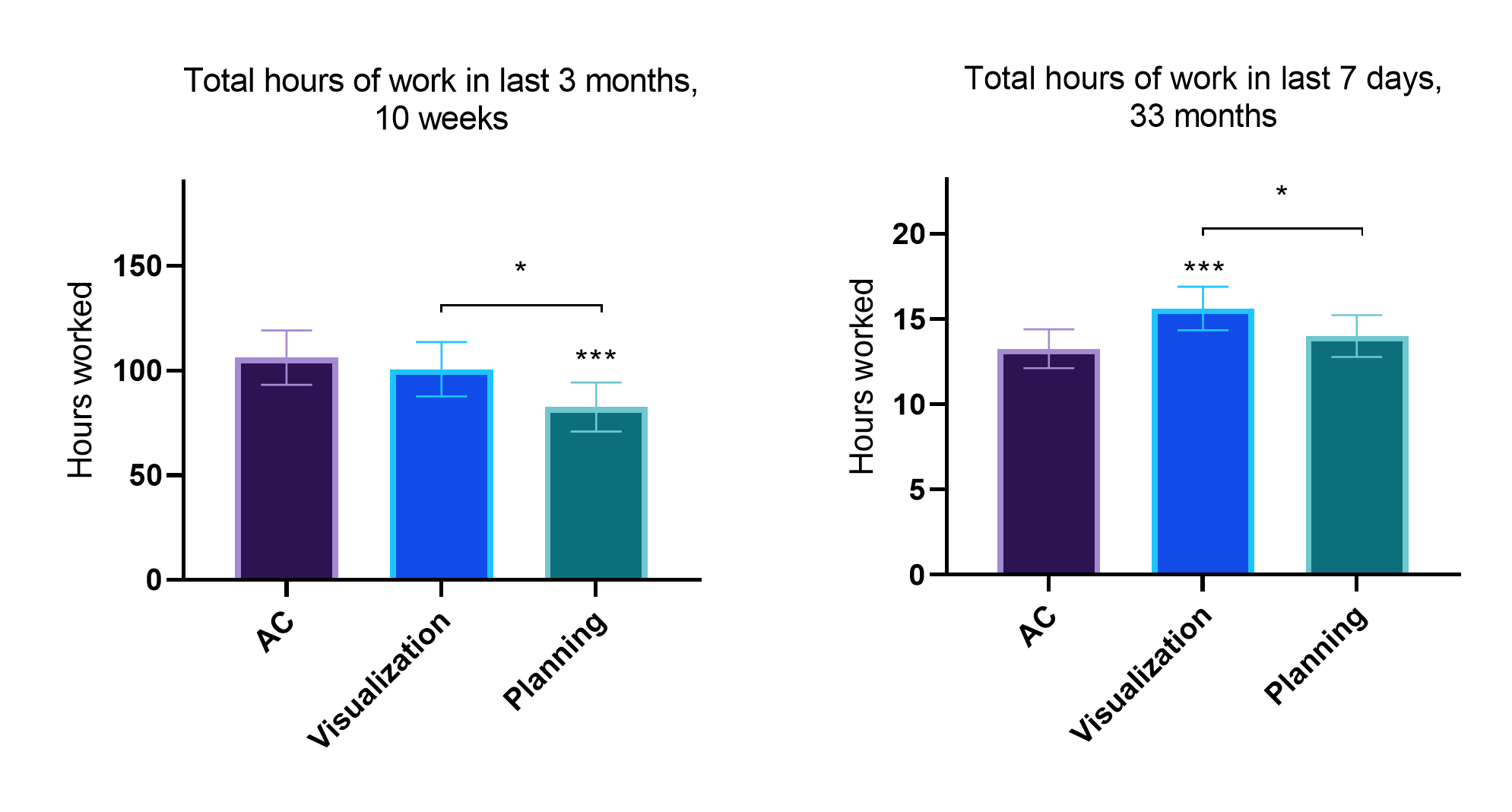

Supplement: jvab052_John_Orkin_TeachingMaterial [file jvab052_john_orkin_teachingmaterial.zip › Figures/Labor.png]

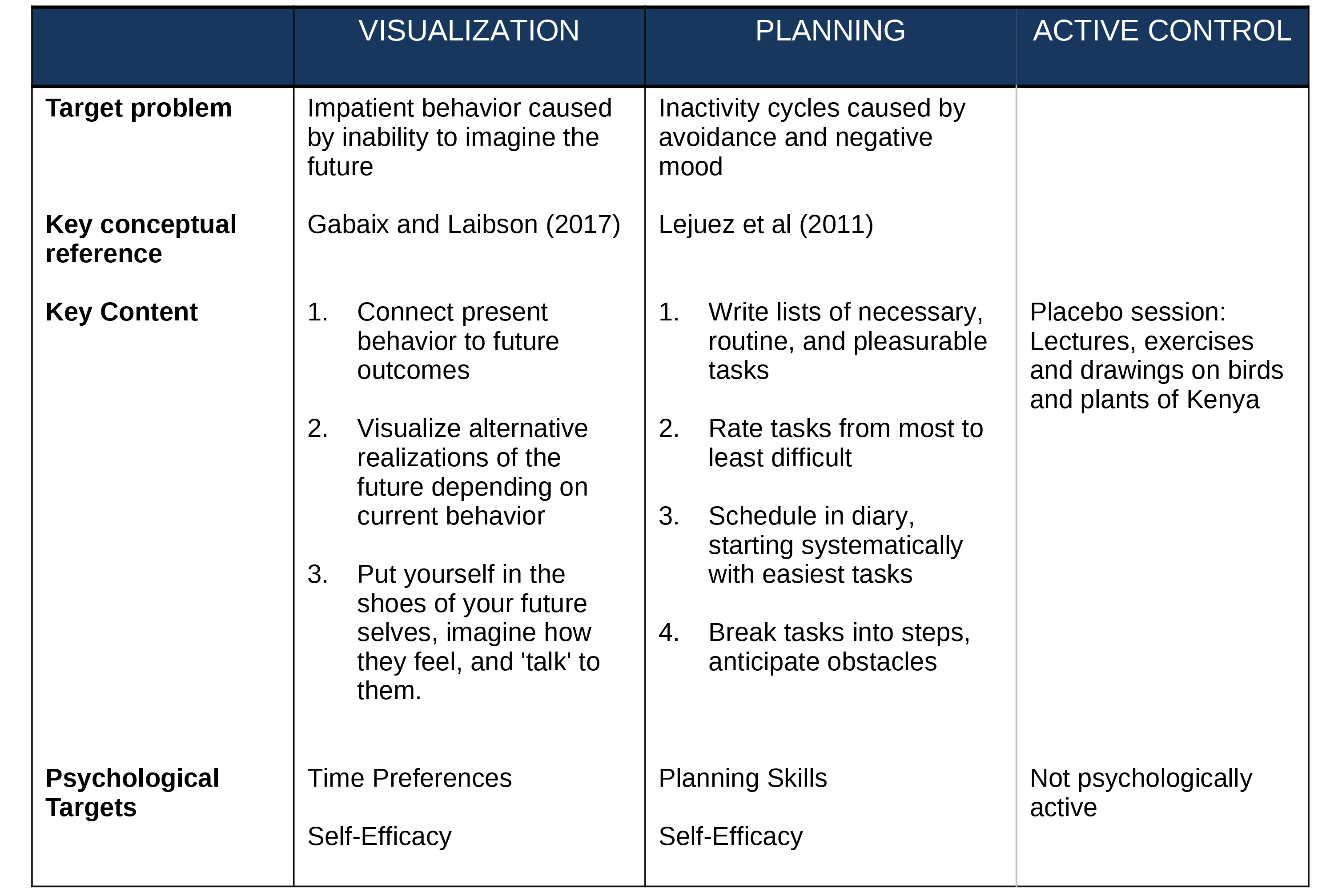

Supplement: jvab052_John_Orkin_TeachingMaterial [file jvab052_john_orkin_teachingmaterial.zip › Figures/Overview_interventions.png]

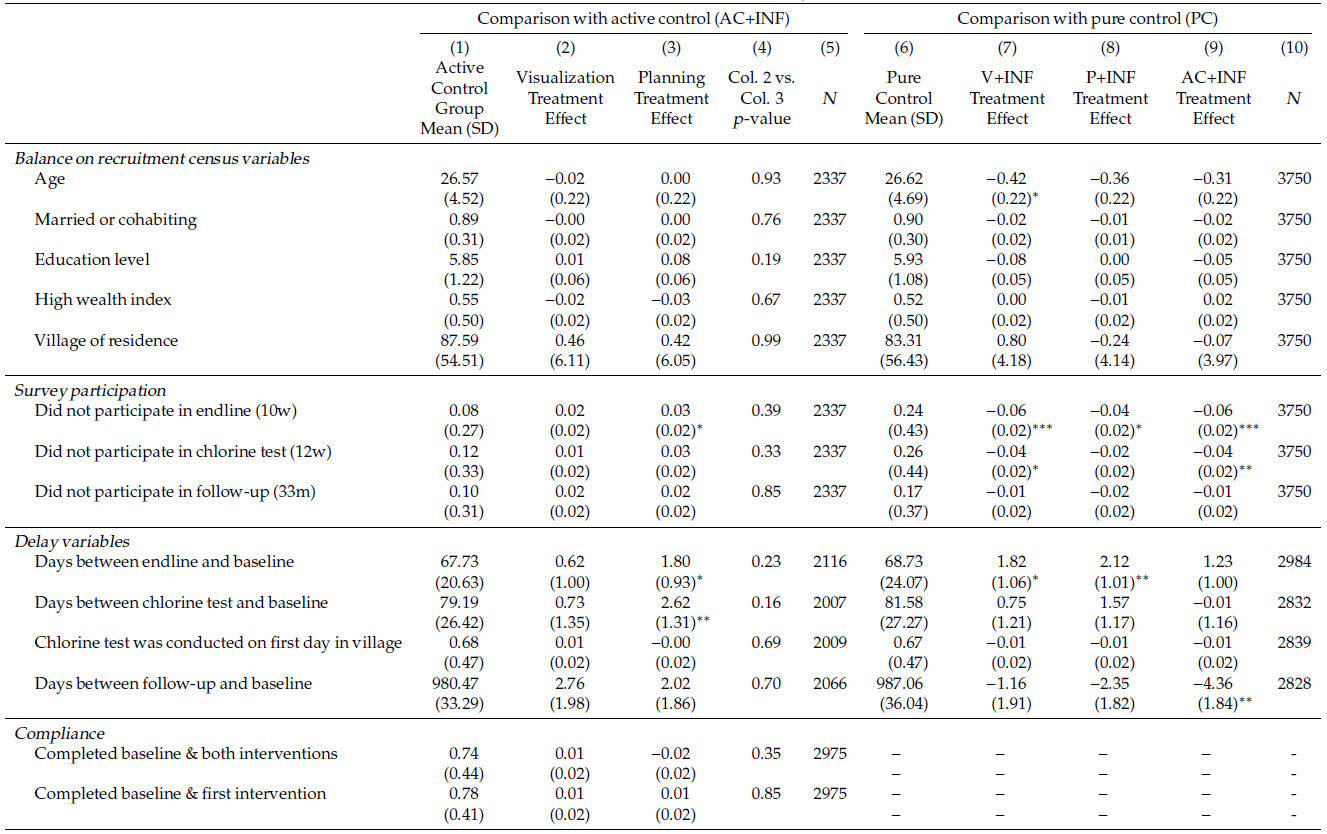

Supplement: jvab052_John_Orkin_TeachingMaterial [file jvab052_john_orkin_teachingmaterial.zip › Figures/Table1.png]

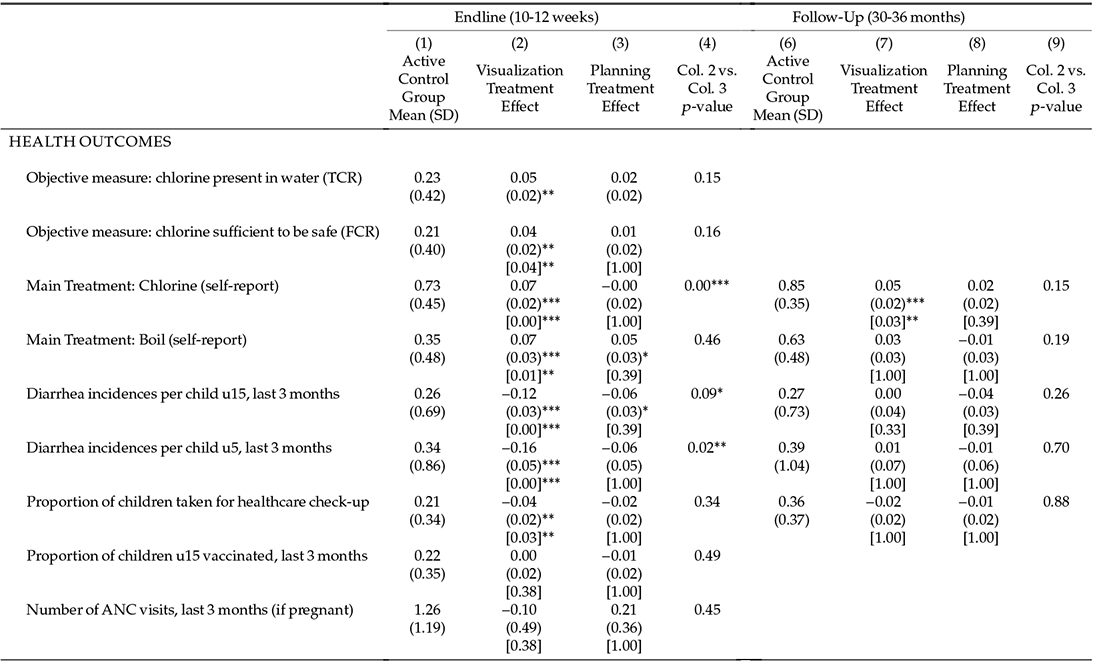

Supplement: jvab052_John_Orkin_TeachingMaterial [file jvab052_john_orkin_teachingmaterial.zip › Figures/Table2_health_cropped.png]

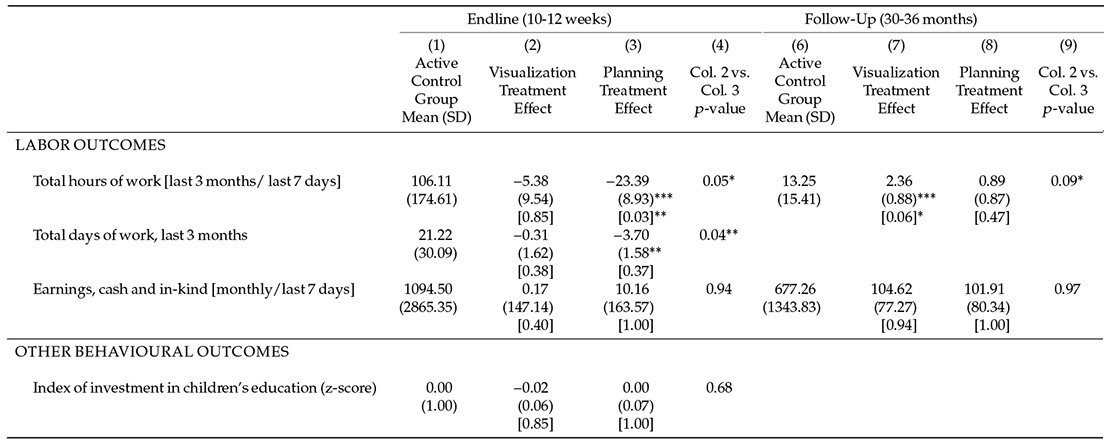

Supplement: jvab052_John_Orkin_TeachingMaterial [file jvab052_john_orkin_teachingmaterial.zip › Figures/Table2_labor_corr.png]

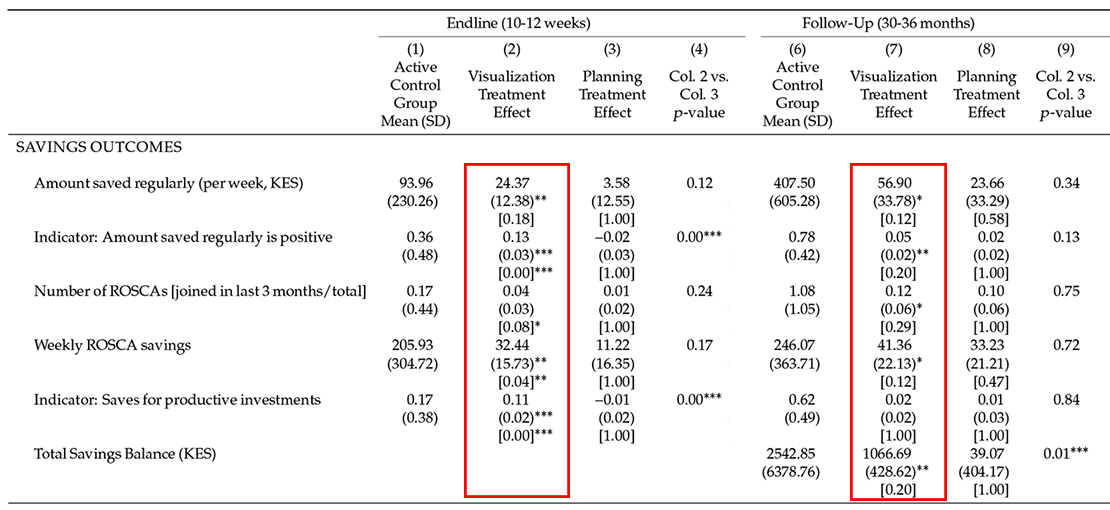

Supplement: jvab052_John_Orkin_TeachingMaterial [file jvab052_john_orkin_teachingmaterial.zip › Figures/Table2_savings_200red.png]

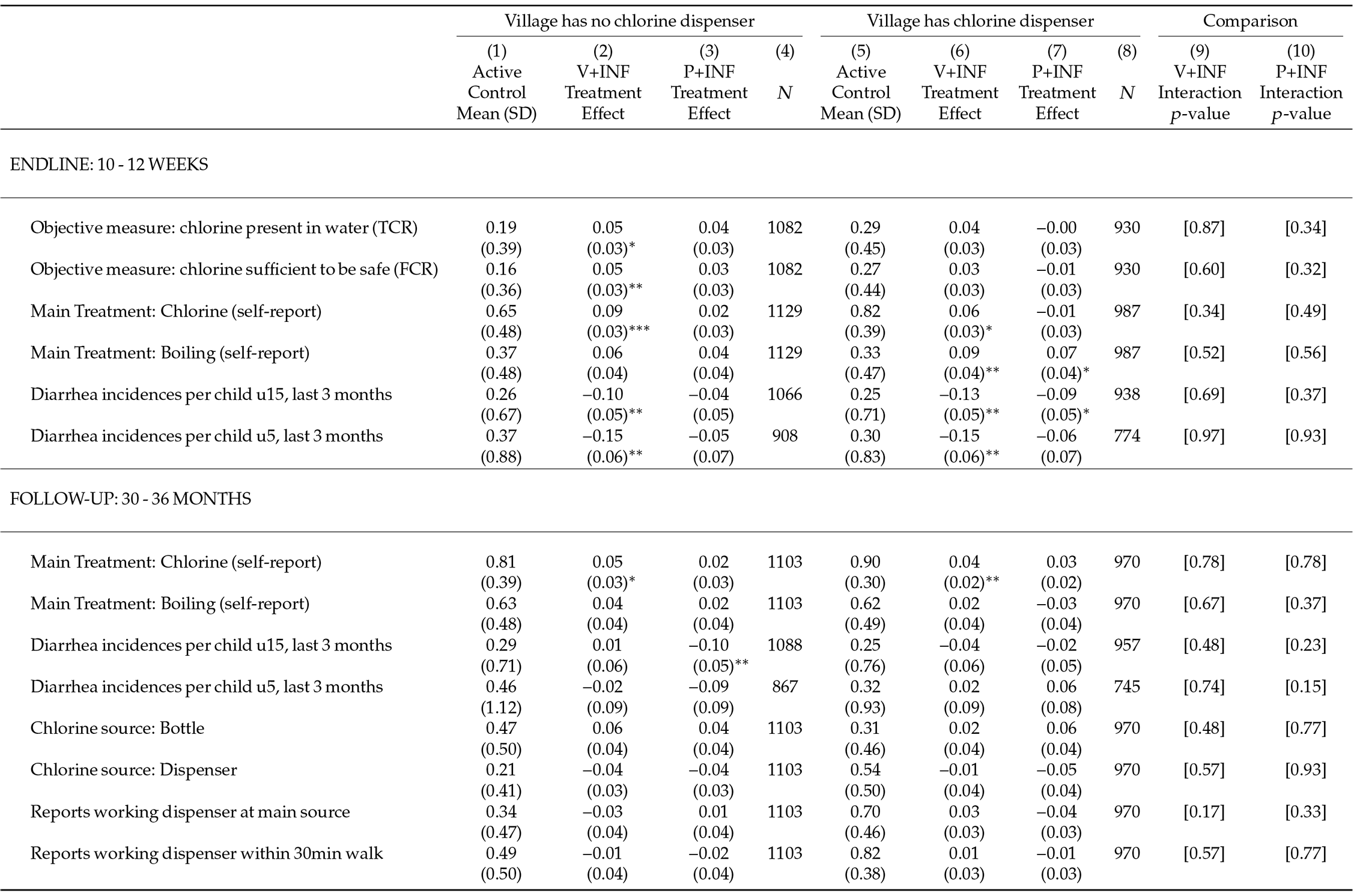

Supplement: jvab052_John_Orkin_TeachingMaterial [file jvab052_john_orkin_teachingmaterial.zip › Figures/Table3_dispenser_corr.png]

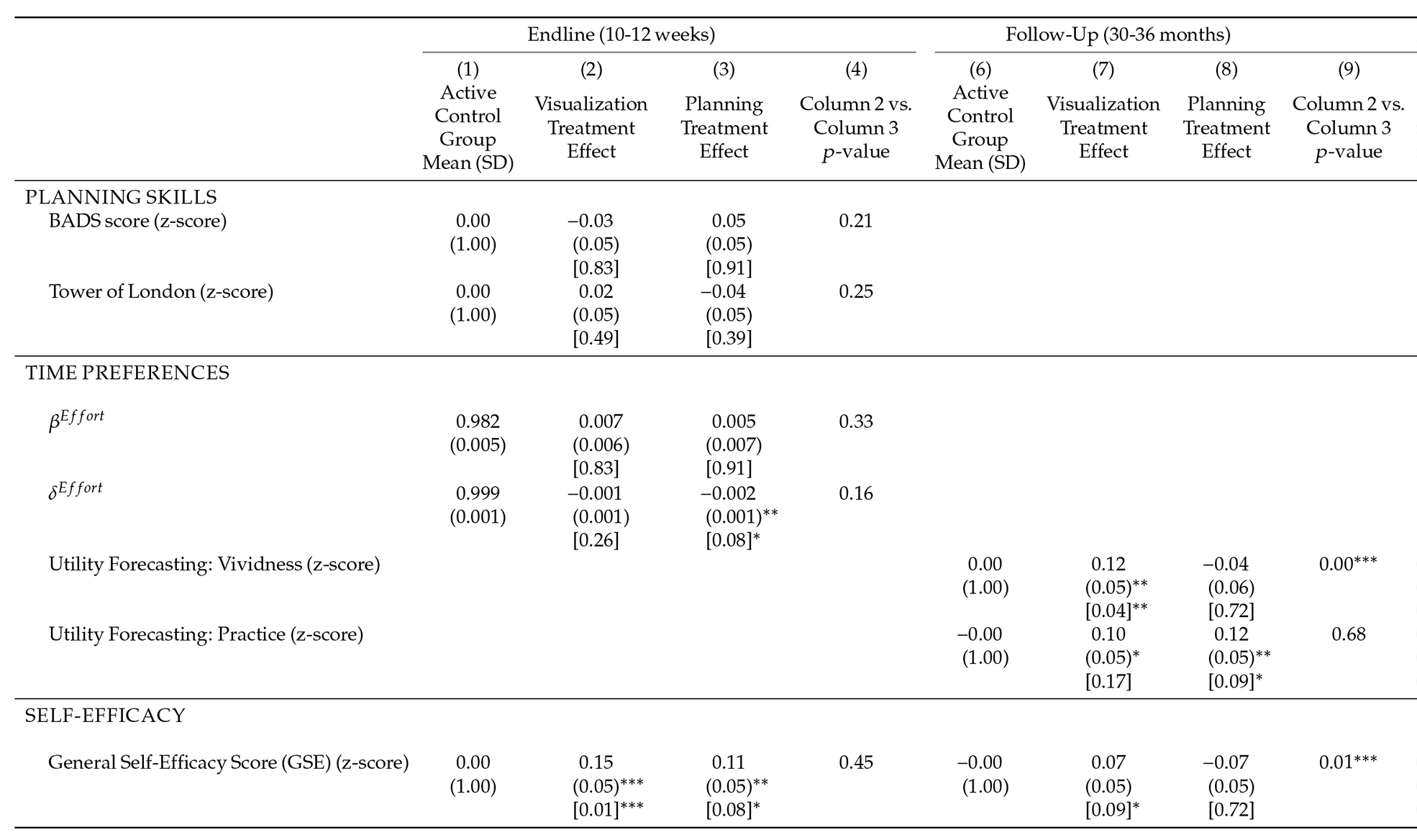

Supplement: jvab052_John_Orkin_TeachingMaterial [file jvab052_john_orkin_teachingmaterial.zip › Figures/Table4_psych_corr.png]

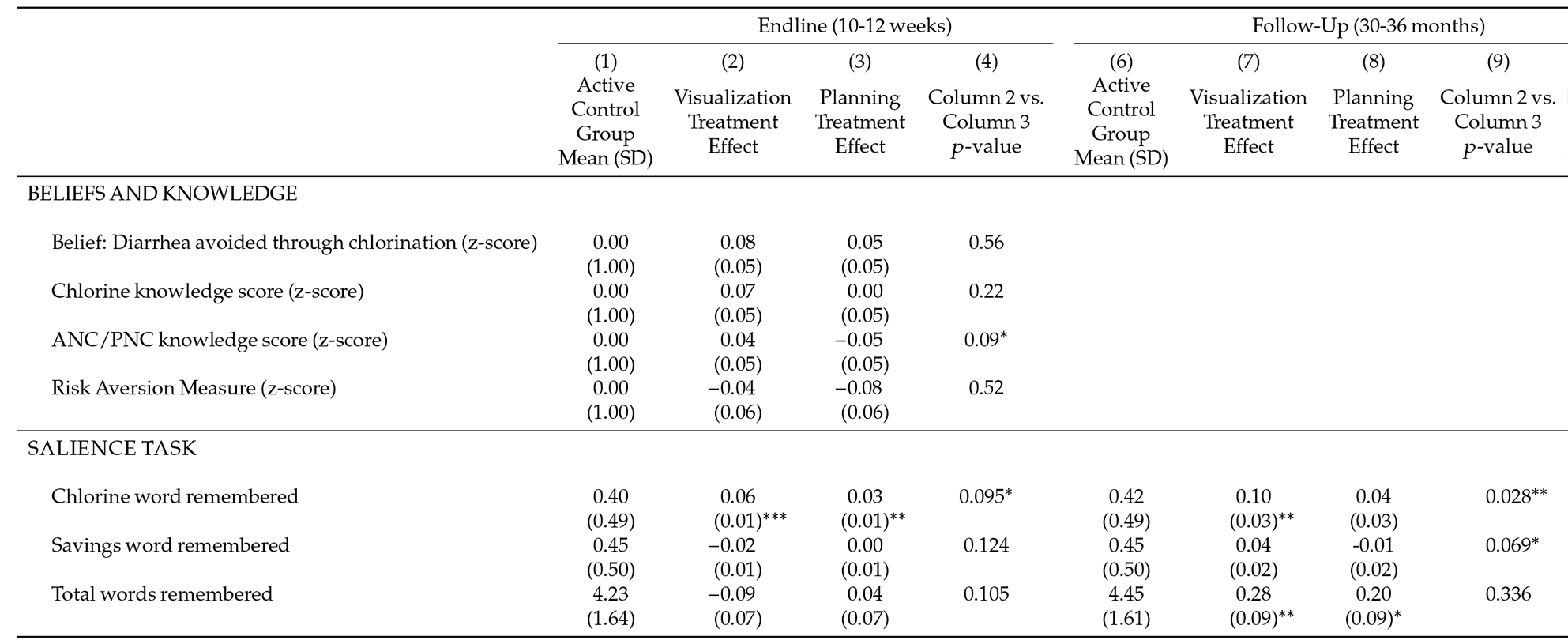

Supplement: jvab052_John_Orkin_TeachingMaterial [file jvab052_john_orkin_teachingmaterial.zip › Figures/Table5_alternative_corr.png]

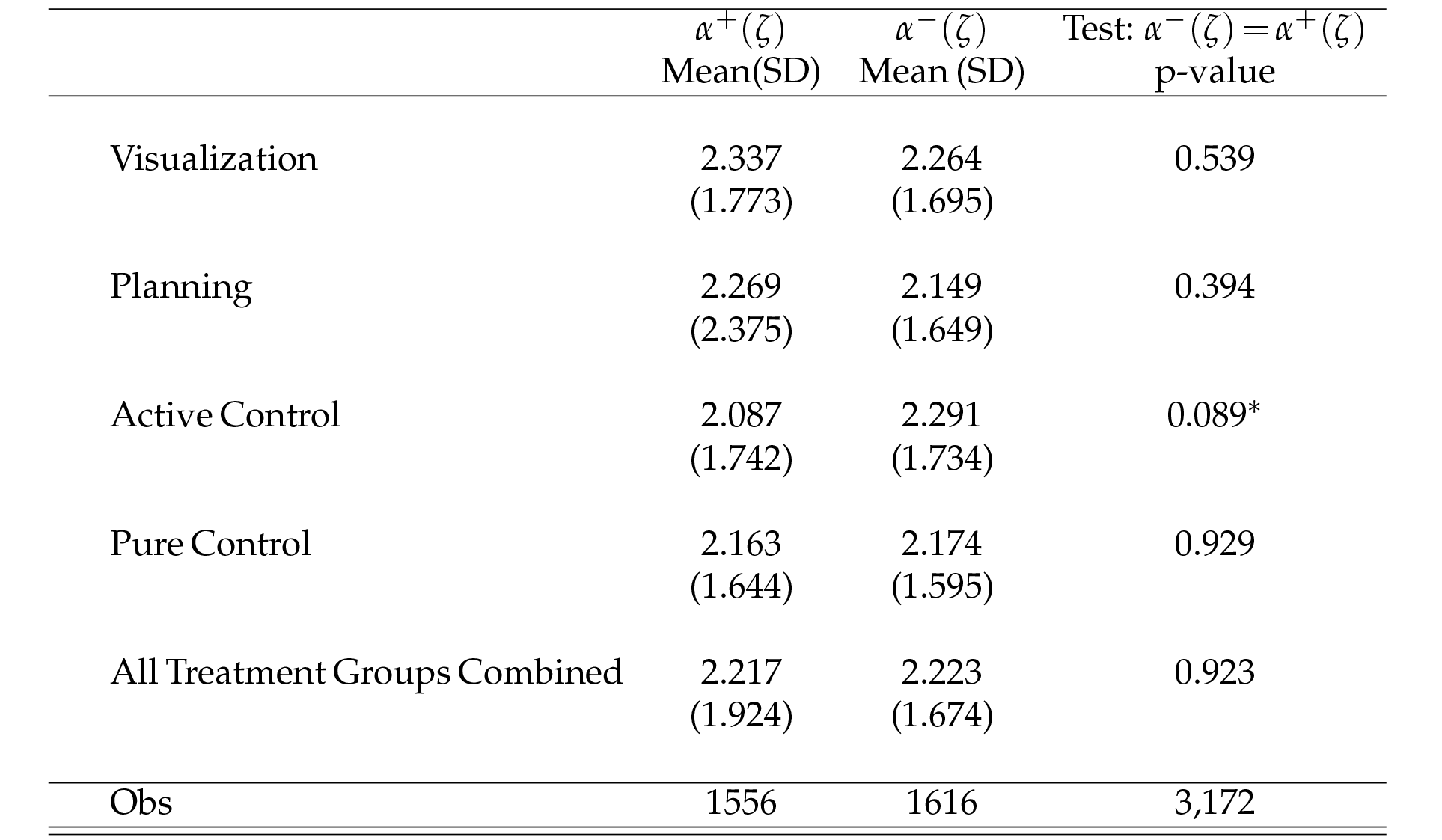

Supplement: jvab052_John_Orkin_TeachingMaterial [file jvab052_john_orkin_teachingmaterial.zip › Figures/Table_demand_effects.png]

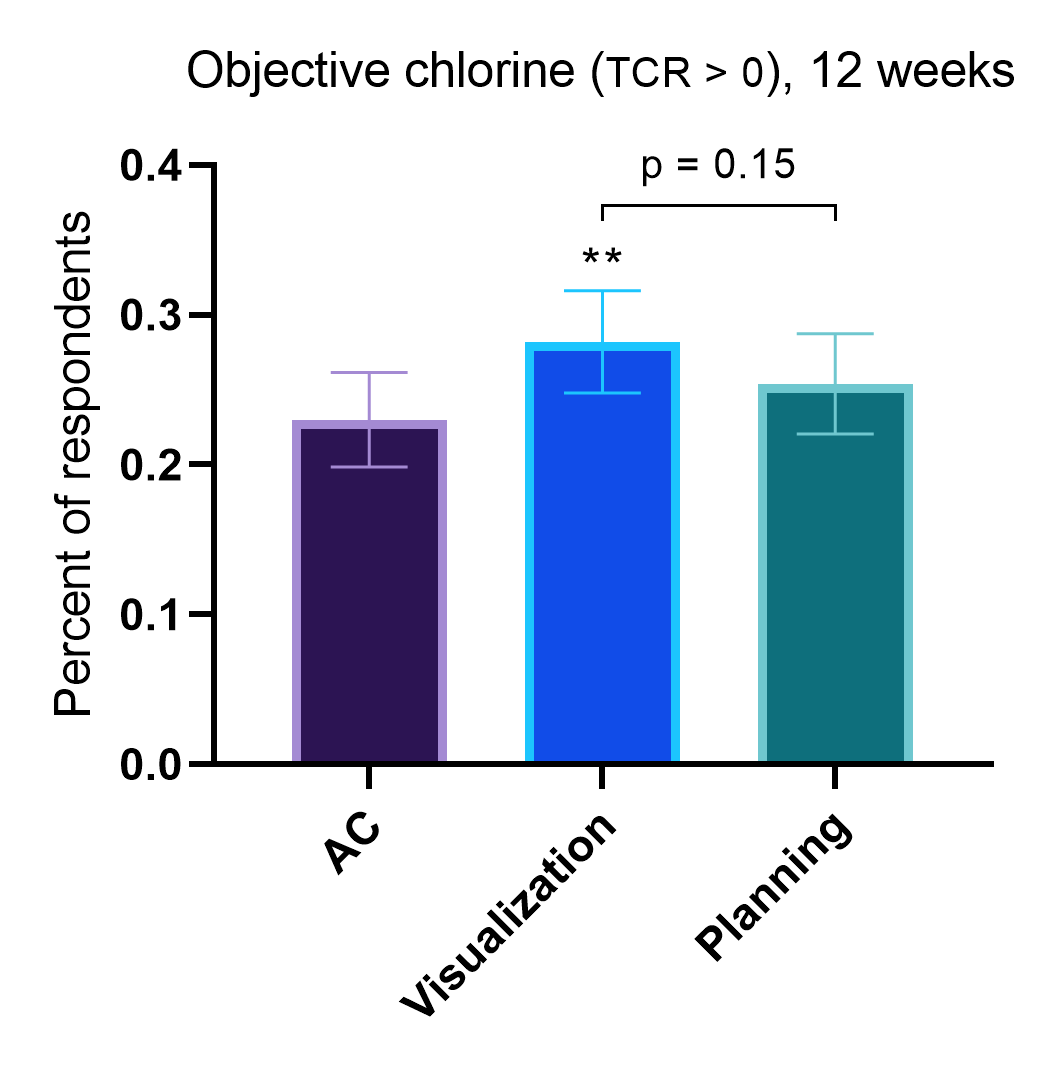

Supplement: jvab052_John_Orkin_TeachingMaterial [file jvab052_john_orkin_teachingmaterial.zip › Figures/TCR.png]

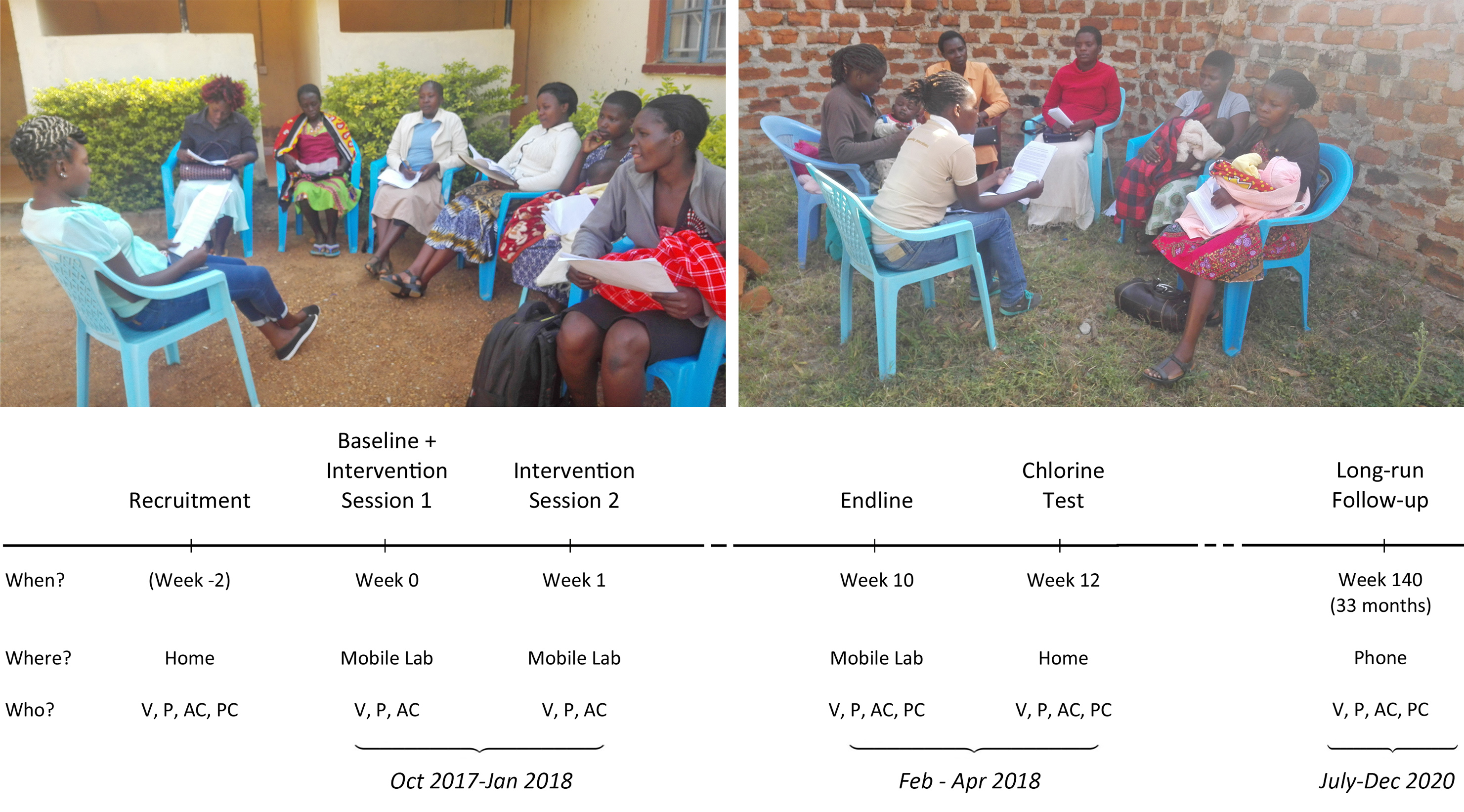

Supplement: jvab052_John_Orkin_TeachingMaterial [file jvab052_john_orkin_teachingmaterial.zip › Figures/Timeline_LR_photos.jpg]

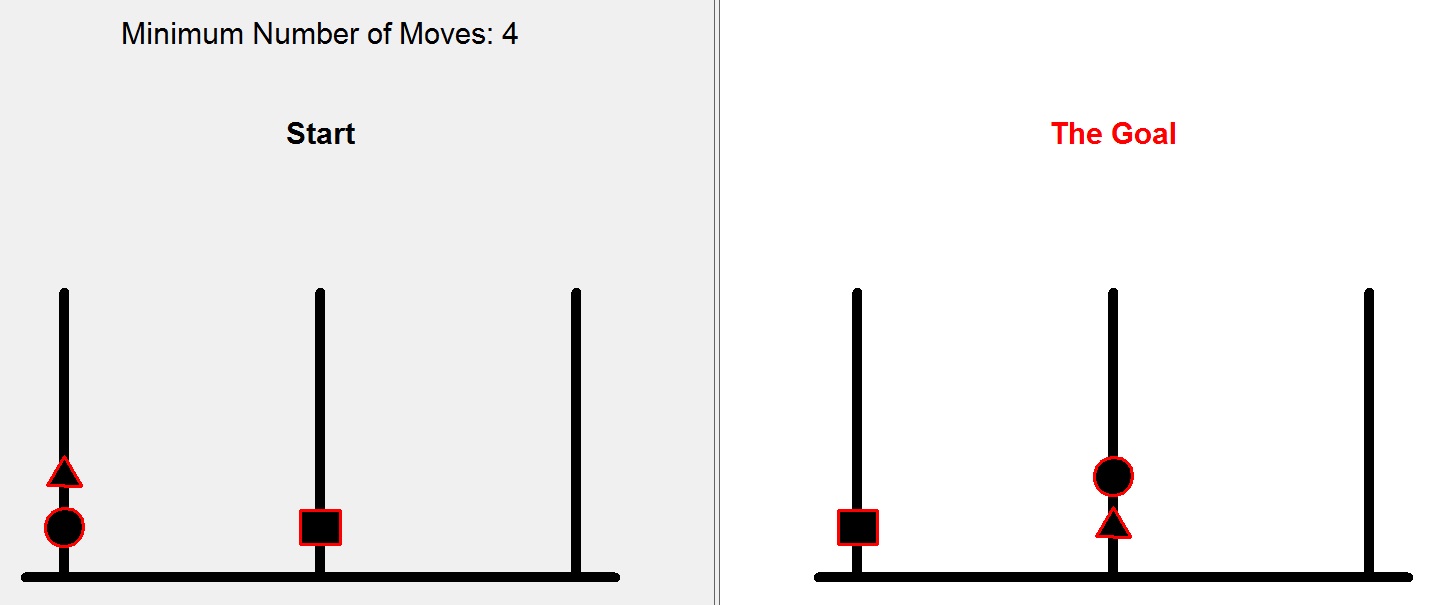

Supplement: jvab052_John_Orkin_TeachingMaterial [file jvab052_john_orkin_teachingmaterial.zip › Figures/ToL_Q4.jpg]

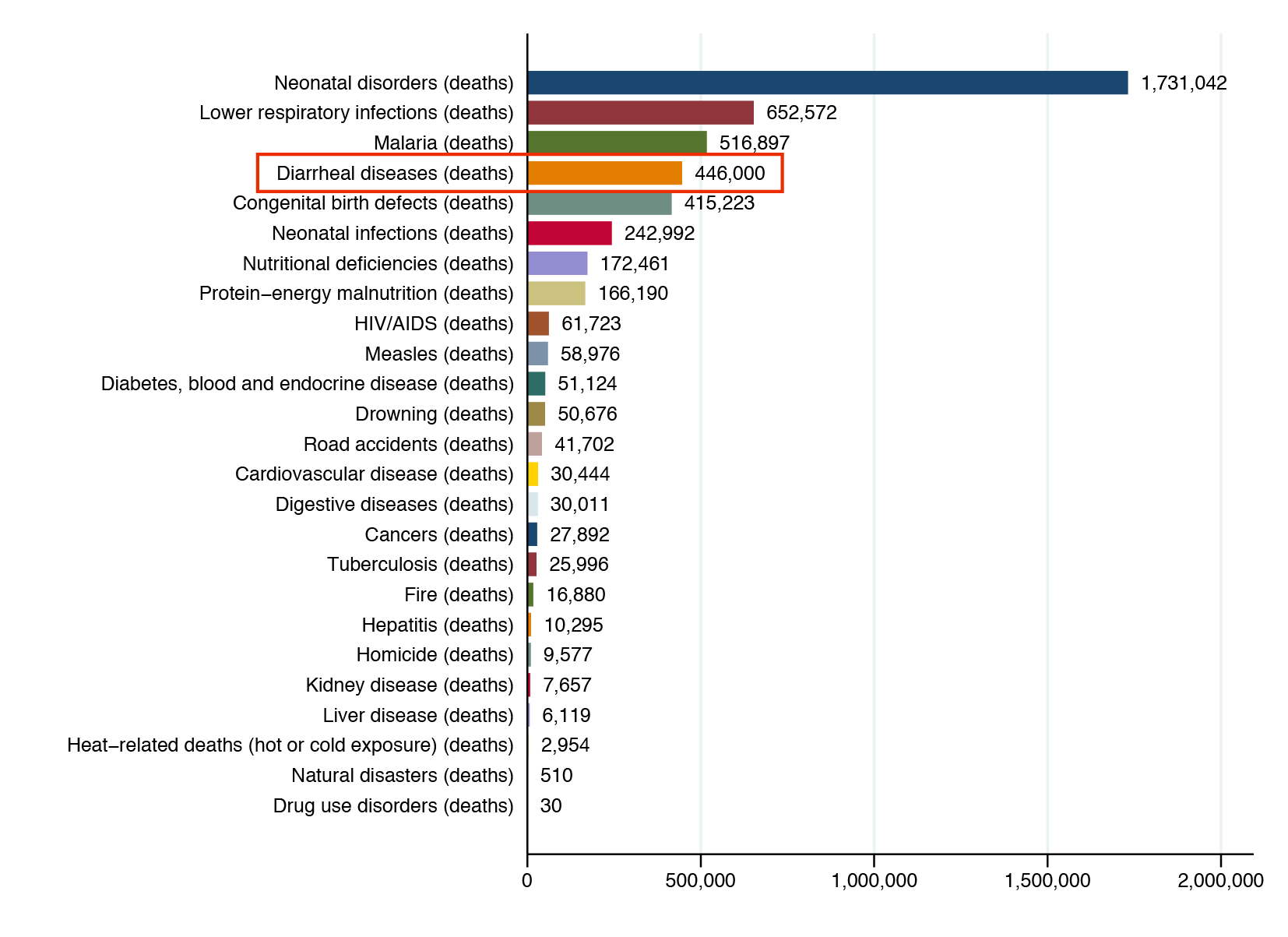

Supplement: jvab052_John_Orkin_TeachingMaterial [file jvab052_john_orkin_teachingmaterial.zip › Figures/Under5_box.png]

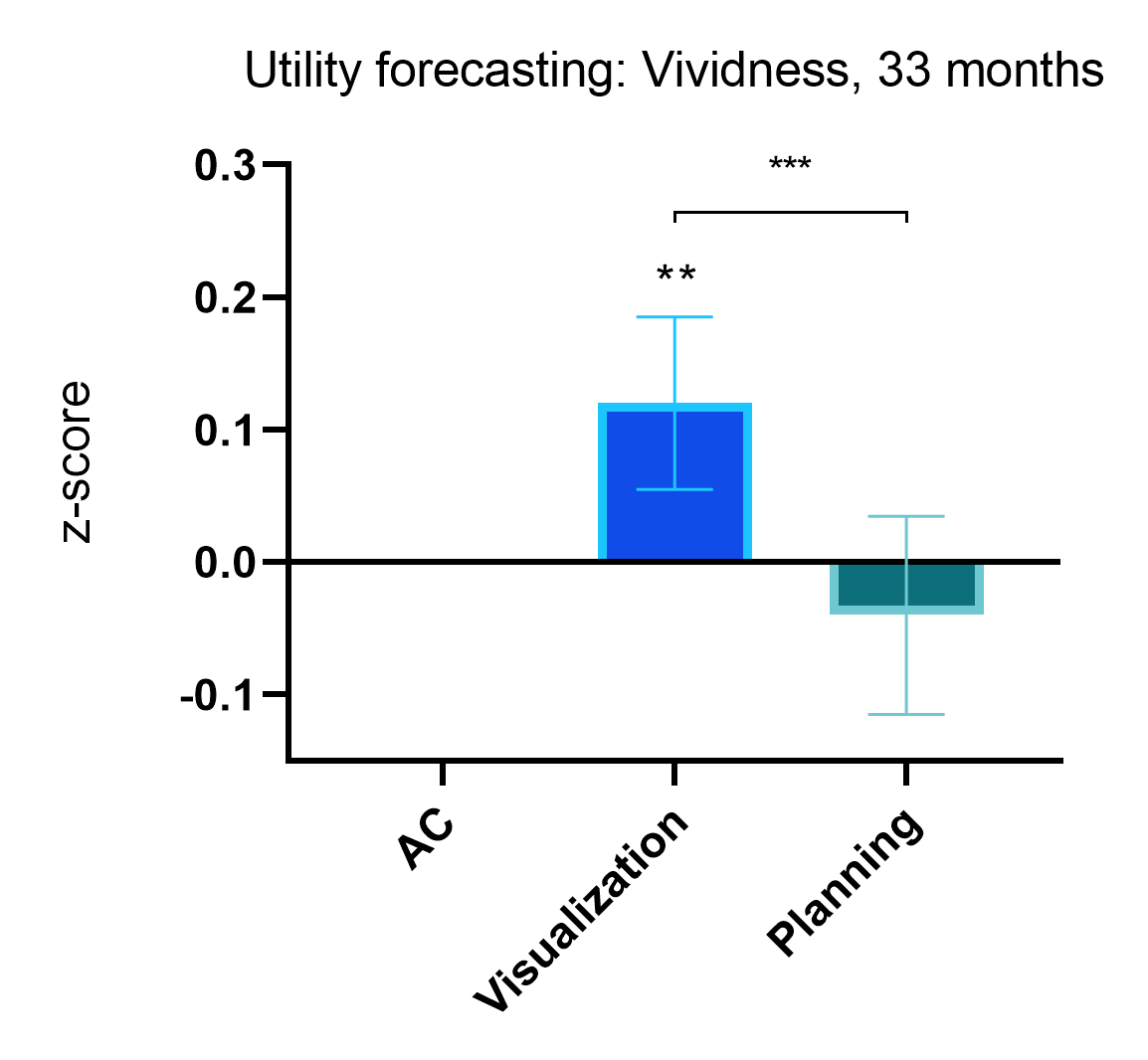

Supplement: jvab052_John_Orkin_TeachingMaterial [file jvab052_john_orkin_teachingmaterial.zip › Figures/vividness_LR.png]

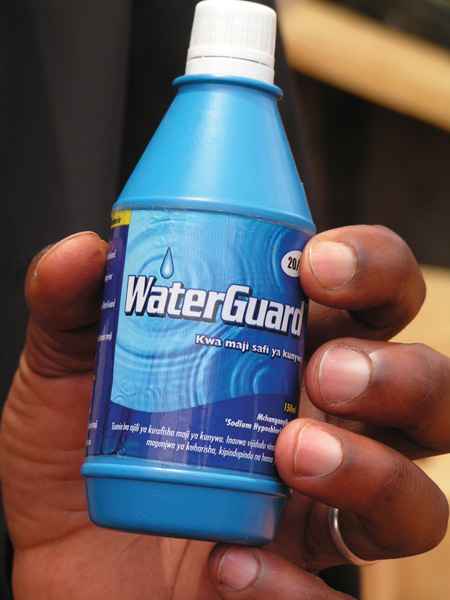

Supplement: jvab052_John_Orkin_TeachingMaterial [file jvab052_john_orkin_teachingmaterial.zip › Figures/waterguardinhand.jpg]
