# Supplementary material for: Can Simple Psychological Interventions Increase Preventive Health Investment?
Source: J Eur Econ Assoc. 2021 Nov 30;20(3):1001–47. doi: 10.1093/jeea/jvab052 (PMC9194950; doi:10.1093/jeea/jvab052)
Supplement: jvab052_John_Orkin_TeachingMaterial [file jvab052_john_orkin_teachingmaterial.zip › John_Orkin_teaching_material.pdf]

Can Simple Psychological Interventions Increase Preventive Health Investment?

Anett John and Kate Orkin

February 2022

Introduction

- Broad evidence that people forgo profitable investments
  - Health: demand for bednets, chlorinating water, deworming pills, using improved cookstoves

Introduction

- Broad evidence that people forgo profitable investments
  - Health: demand for bednets, chlorinating water, deworming pills, using improved cookstoves
- ....even when standard barriers have been removed or minimized
  - Little or no effect of information and marketing (Kremer et al 2011, Dupas 2009)
  - Even tiny fees dramatically reduce take-up for bednets, chlorine, soap (reviewed in Kremer and Glennerster 2011, Dupas and Miguel 2017)
  - Our setting: Chlorine cheap or free, info provided, minimal hassle (Null et al 2018: Adherence 23 percent after two years)

Introduction

- Broad evidence that people forgo profitable investments
  - Health: demand for bednets, chlorinating water, deworming pills, using improved cookstoves
- ....even when standard barriers have been removed or minimized
  - Little or no effect of information and marketing (Kremer et al 2011, Dupas 2009)
  - Even tiny fees dramatically reduce take-up for bednets, chlorine, soap (reviewed in Kremer and Glennerster 2011, Dupas and Miguel 2017)
  - Our setting: Chlorine cheap or free, info provided, minimal hassle (Null et al 2018: Adherence 23 percent after two years)
- Role of psychological barriers?
- This paper: Psychological workshops to target (domain-general) preferences, beliefs, cognitive constraints. Test if these lead to changes in behavior.

Under-5 mortality

Diarrheal diseases are the fourth largest cause of under 5 mortality worldwide

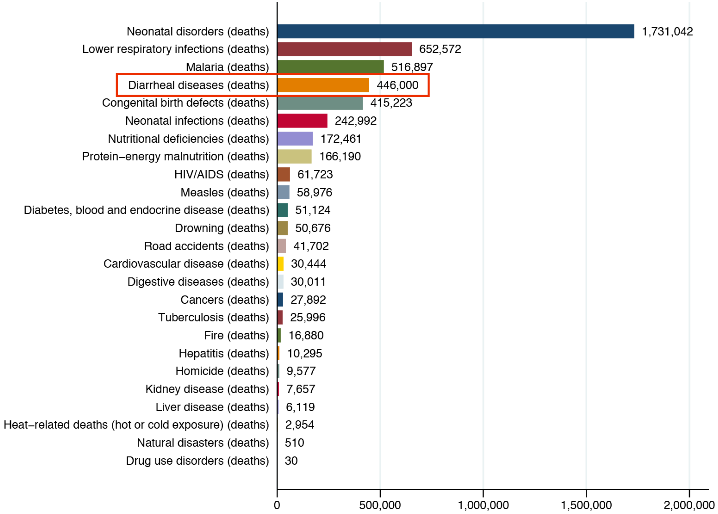

The Targeted Behaviour: Treating Water with Chlorine

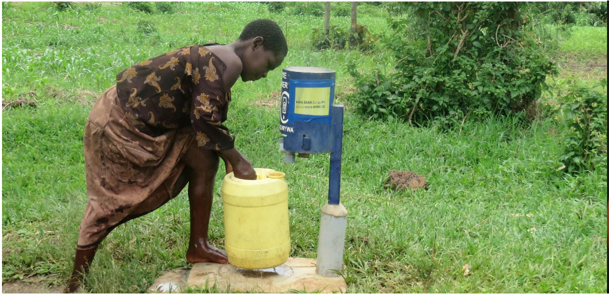

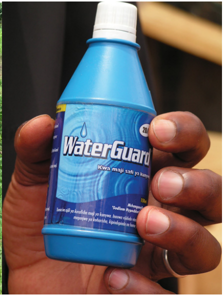

- **Low costs:** Chlorine for drinking water is cheap and readily available
  - USD 0.20 per bottle (= 1 month) (average monthly earnings: $90)
  - Interventions providing free dispensers at water sources (Kremer et al 2011, Null et al 2018) Kremer et al 2011
- **Low use:** 23% (control: 3%) even with dispensers + community health promoters (Null et al 2018)
- **Potential non-monetary cost:** time, attention, taste habituation

Psychological barriers to investment may be within...

1. **Preferences**: Might not value the future enough; costs of chlorinating today outweigh benefit of healthy children tomorrow $\rightarrow$ impatience & present bias
2. **Beliefs**: Might not believe own actions can influence outcomes (self-efficacy). Might not know benefits of chlorination (information)
3. **Constraints**: Might be unable to plan and execute chlorination (e.g. forgetting, disorganized)

This study:

- Use light-touch psychological interventions to study if
  1. (some of) these targets are malleable
  2. they drive behavior in different domains
- Randomized controlled trial ($N = 3750$) in Western Kenya.
- Cross-randomize reduction in psychological and material barriers.

This Study

- 205 villages in Western Kenya (Kakamega and Bungoma), $N = 3750$
  - Women aged $18 - 35$, $\phi\, children = 2.7$, daily wage $150 - 400$ Ksh.
- **Interventions:** Two-hour two-session group workshops.
  1. **Visualization** workshop: targets time preferences
     - ★ Idea: We are impatient because present utility is easier to imagine than future utility (Alan & Ertac 2018, Gabaix & Laibson 2017)
     - ★ Approach: Visualization of alternative realizations of the future

This Study

- 205 villages in Western Kenya (Kakamega and Bungoma), $N = 3750$
  - Women aged $18 - 35$, $\phi\, children = 2.7$, daily wage $150 - 400$ Ksh.

- **Interventions:** Two-hour two-session group workshops.
  1. **Visualization** workshop: targets time preferences
     - ★ Idea: We are impatient because present utility is easier to imagine than future utility (Alan & Ertac 2018, Gabaix & Laibson 2017)
     - ★ Approach: Visualization of alternative realizations of the future
  2. **Planning** workshop: targets planning skills (cognitive constraints)
     1. Idea: People get stuck in cycles of avoidance/inactivity and low mood
     2. Approach: Behavioural Activation (adapted from Richards & Whyte 2011)

Both workshops presume agency, and thus may affect **self-efficacy** (beliefs)

This Study

- 205 villages in Western Kenya (Kakamega and Bungoma), $N = 3750$
  - Women aged $18 - 35$, $\phi\, children = 2.7$, daily wage $150 - 400$ Ksh.

- **Interventions:** Two-hour two-session group workshops.
  1. **Visualization** workshop: targets time preferences
     - Idea: We are impatient because present utility is easier to imagine than future utility (Alan & Ertac 2018, Gabaix & Laibson 2017)
     - Approach: Visualization of alternative realizations of the future
  2. **Planning** workshop: targets planning skills (cognitive constraints)
     1. Idea: People get stuck in cycles of avoidance/inactivity and low mood
     2. Approach: Behavioural Activation (adapted from Richards & Whyte 2011)

  Both workshops presume agency, and thus may affect **self-efficacy** (beliefs)

  3. **Placebo** workshop including **information module** (also in P+V)
  4. **Pure control**

- **Cross-cut:** A random half of villages has chlorine dispensers.

Literature and Contributions

Large literature in behavioral & dev econ on psych factors which affect investment. Few studies attempt to change domain-general preferences or beliefs.

- Visualization to target intertemporal choice
  - Lots of interest in psychology (EFT, age-progressed pictures of self). Outcomes in lab *while* intervention administered, $N \leq 50$.
  - Alan & Ertac (2018) use incentivized tasks in Turkish primary schools

$\Rightarrow$ Show effects of V. on real-world behavior of adults, in multiple domains.

Literature and Contributions

Large literature in behavioral & dev econ on psych factors which affect investment. Few studies attempt to change domain-general preferences or beliefs.

- Visualization to target intertemporal choice
  - Lots of interest in psychology (EFT, age-progressed pictures of self). Outcomes in lab *while* intervention administered, $N \leq 50$.
  - Alan & Ertac (2018) use incentivized tasks in Turkish primary schools

$\Rightarrow$ Show effects of V. on real-world behavior of adults, in multiple domains.

- Behavioral activation to target planning skills
  - Psychotherapy approaches (e.g. CBT) typically intense and on subpopulations. (Blattman et al 2017, Baranov et al 2018, Heller et al 2017).
  - Implementation interventions and planning prompts targeting specific behaviours (Duckworth et al. 2013, Milkman et al 2011)

$\Rightarrow$ Show econ&health effects of light-touch, domain-general planning intervention.
$\Rightarrow$ Comparison of two interventions allows to study which psych targets are most malleable & most affect behavior.

Literature and Contributions

Large literature in behavioral & dev econ on psych factors which affect investment. Few studies attempt to change domain-general preferences or beliefs.

- Visualization to target intertemporal choice
  - Lots of interest in psychology (EFT, age-progressed pictures of self). Outcomes in lab *while* intervention administered, $N \leq 50$.
  - Alan & Ertac (2018) use incentivized tasks in Turkish primary schools

  $\Rightarrow$ Show effects of V. on real-world behavior of adults, in multiple domains.

- Behavioral activation to target planning skills
  - Psychotherapy approaches (e.g. CBT) typically intense and on subpopulations. (Blattman et al 2017, Baranov et al 2018, Heller et al 2017).
  - Implementation interventions and planning prompts targeting specific behaviours (Duckworth et al. 2013, Milkman et al 2011)

  $\Rightarrow$ Show econ&health effects of light-touch, domain-general planning intervention.
  $\Rightarrow$ Comparison of two interventions allows to study which psych targets are most malleable & most affect behavior.

Literature on increasing preventive health in low-income countries

- Information, free provision, vouchers (Ashraf et al 2010a + 2010b, many more).
  $\Rightarrow$ Show effects of light-touch, domain-general psych interventions.

Preview of Results

- After 12 weeks, visualization increases objective chlorination, reduces diarrhea among children, and increases savings.
- Effects on chlorination and savings persist after almost three years. Labor supply increases.
- Effects of the planning intervention are weaker and largely insignificant.
- Mechanisms:
  - No change in planning skills or conventional measures of time preferences
    - ⋆ Although visualization increases participants' skill in mentally forecasting their future (Gabaix and Laibson 2017)
  - Both interventions increase self-efficacy (stronger & more persistent in V)
  - Results not explained by information, risk preferences, experimenter demand, salience

Theory of Change: Visualization

- E.g. *Gabaix and Laibson (2017)*: A perfectly patient decisionmaker forms beliefs about utility from a future reward $u_t \sim \mathcal{N}(\mu, \sigma_u^2)$ by combining her prior μ with noisy & unbiased simulations of the reward s_t. ▸ Diagram
- Signals $s_t = u_t + \epsilon_t$ with $\epsilon_t \sim \mathcal{N}(0, \sigma_{\epsilon_t}^2)$ and $\sigma_{\epsilon_t}^2$ increasing in t.

Theory of Change: Visualization

- E.g. *Gabaix and Laibson (2017)*: A perfectly patient decisionmaker forms beliefs about utility from a future reward $u_t \sim \mathcal{N}(\mu, \sigma_u^2)$ by combining her prior μ with noisy & unbiased simulations of the reward s_t. ▸ Diagram
- Signals $s_t = u_t + \epsilon_t$ with $\epsilon_t \sim \mathcal{N}(0, \sigma_{\epsilon_t}^2)$ and $\sigma_{\epsilon_t}^2$ increasing in t.
- For simplicity, let $\mu = 0$. Then average posterior of u_t is $D(t)u_t$, where

$$D(t) = 1/(1 + {}^{\sigma_{\epsilon_t}^2}/_{\sigma_u^2})$$

is the *as-if* discounting function. $D(t)$ decreases in t, and is hyperbolic when $\sigma_{\epsilon_t}^2 = \sigma_\epsilon^2 t$. Result: "present-biased" behavior with preference reversals.

Theory of Change: Visualization

- E.g. *Gabaix and Laibson (2017)*: A perfectly patient decisionmaker forms beliefs about utility from a future reward $u_t \sim \mathcal{N}(\mu, \sigma_u^2)$ by combining her prior μ with noisy & unbiased simulations of the reward s_t. Diagram
- Signals $s_t = u_t + \epsilon_t$ with $\epsilon_t \sim \mathcal{N}(0, \sigma_{\epsilon_t}^2)$ and $\sigma_{\epsilon_t}^2$ increasing in t.
- For simplicity, let $\mu = 0$. Then average posterior of u_t is $D(t)u_t$, where

$$D(t) = 1/(1 + \sigma^2_{\epsilon_t}/\sigma^2_u)$$

  is the *as-if* discounting function. $D(t)$ decreases in t, and is hyperbolic when $\sigma_{\epsilon_t}^2 = \sigma_\epsilon^2 t$. Result: "present-biased" behavior with preference reversals.

- *Implication*: Any intervention which improves forecasting ability (reduces $\sigma_{\epsilon_t}^2$) will lead to more patient behavior
  - Examples: more time/effort thinking about problem (Imas et al 2020), higher imaginative capacity, higher intelligence.

Hypothesis: V leads to more precise utility forecasts. Outside model, stronger mapping: present behavior $\rightarrow$ future outcomes (self-efficacy). Result: Weigh future benefits more heavily against current costs $\rightarrow$ more patient behavior.

Theory of Change: Planning

Intervention based on psychotherapy approach "Behavioral Activation." Draws on literature on instrumental reinforcement and motivation (Lejuez et al 2011).

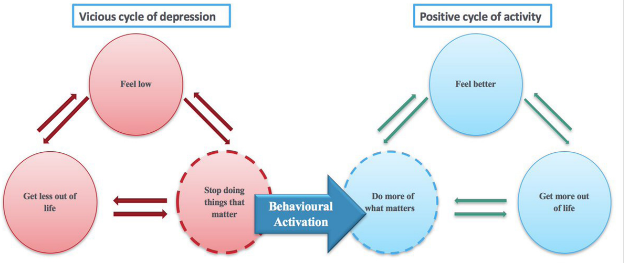

Behavioral activation teaches simple, structured skills to help people "get going" and re-engage with meaningful activities. Key: Start with easiest tasks, earn sense of accomplishment, which improves mood and increases the likelihood they persist with the task.

Hypothesis: P will increase ability to make and stick to plans. 'Mastery experiences' likely to increase self-efficacy (Bandura 1997).

Interventions

	VISUALIZATION	PLANNING	ACTIVE CONTROL
**Target problem**	Impatient behavior caused by inability to imagine the future	Inactivity cycles caused by avoidance and negative mood	
**Key conceptual reference**	Gabaix and Laibson (2017)	Lejuez et al (2011)	
**Key Content**	1. Connect present behavior to future outcomes<br>2. Visualize alternative realizations of the future depending on current behavior<br>3. Put yourself in the shoes of your future selves, imagine how they feel, and 'talk' to them.	1. Write lists of necessary, routine, and pleasurable tasks<br>2. Rate tasks from most to least difficult<br>3. Schedule in diary, starting systematically with easiest tasks<br>4. Break tasks into steps, anticipate obstacles	Placebo session: Lectures, exercises and drawings on birds and plants of Kenya
**Psychological Targets**	Time Preferences<br>Self-Efficacy	Planning Skills<br>Self-Efficacy	Not psychologically active

All treatments: Two 2h-sessions incl. interactive lectures, case stories, exercises, and drawings.

Timeline

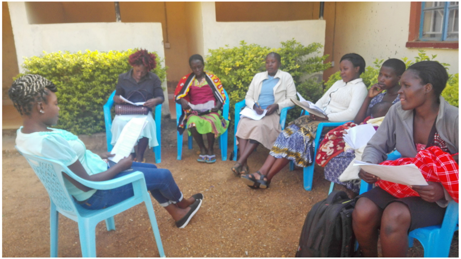

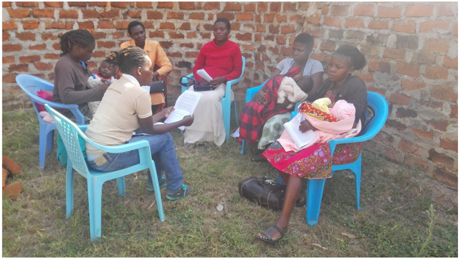

	Recruitment	Baseline + Intervention Session 1	Intervention Session 2	Endline	Chlorine Test	Long-run Follow-up
When?	(Week -2)	Week 0	Week 1	Week 10	Week 12	Week 140 (33 months)
Where?	Home	Mobile Lab	Mobile Lab	Mobile Lab	Home	Phone
Who?	V, P, AC, PC	V, P, AC	V, P, AC	V, P, AC, PC	V, P, AC, PC	V, P, AC, PC
		Oct 2017-Jan 2018		*Feb - Apr 2018*		*July-Dec 2020*

Pre-Specified Outcomes of Interest

	10-week	30-36 month
BEHAVIOR		
**Hypothesis 1: the intervention affects water chlorination**		
Presence of any chlorine in household drinking water	Primary	
General water treatment: Indicator for chlorine	Not pre-specified	Primary
**Hypothesis 1a: the intervention affects health outcomes**		
Number of diarrhea episodes per child under 15 in last 3 months	Exploratory	Primary
**Hypothesis 2: the intervention affects future investments**		
Amount saved regularly (weekly, KES)	Secondary	Secondary
Total hours of work	Secondary	Secondary
Education investment index	Secondary	Not measured
PSYCHOLOGICAL MECHANISMS		
**Hypothesis 1: the intervention affects planning ability**		
Behavioral Activation for Depression Scale - Short Form (BADS-SF)	Primary	Not measured
Tower of London task: total moves across all four rounds	Secondary	Not measured
**Hypothesis 2: the intervention affects time preferences**		
β^{Effort} (estimated from the effort discounting task)	Primary	Not measured
Utility forecasting: Vividness rating	Not measured	Primary
**Hypothesis 3: the intervention affects self-efficacy**		
Generalized Self-Efficacy (GSE) scale	Secondary	Primary

Specification Balance and Attrition

Health: Chlorination

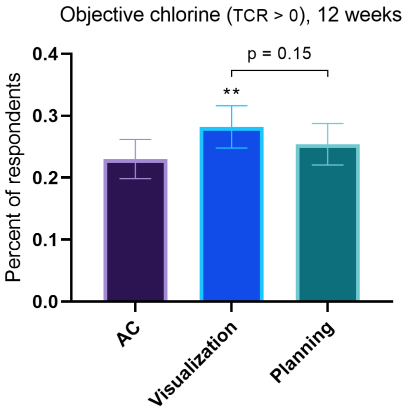

After 12 weeks, share of households who chlorinate increases by 22% (5pp) in V, not significant in P. Boiling of water increases in both V (***) and P (*).

▸ Health Table

No effect of information (active vs pure control).

No significant heterogeneity in health results by dispenser status. ▸ Dispenser Table

Chlorination: Short vs Long Run

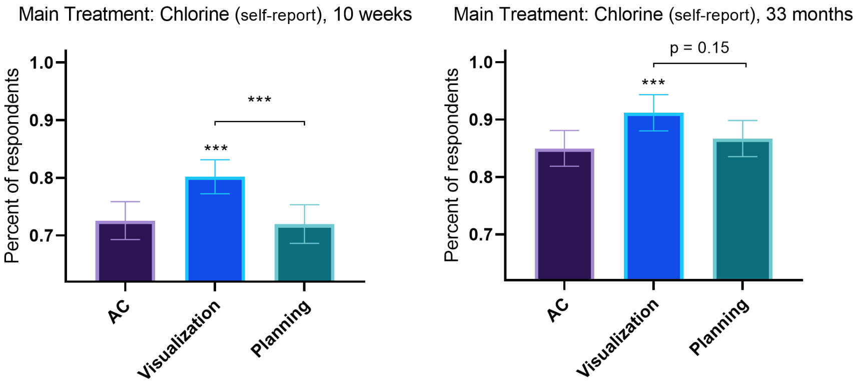

After 10 weeks, 7pp increase in V in those who report that main way to treat water is chlorine.

After 33 months, increase by 5pp in V, not significant in P.

Health: Diarrhea

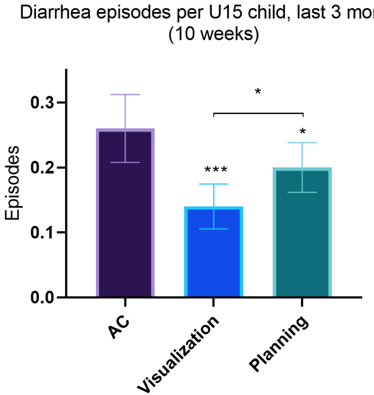

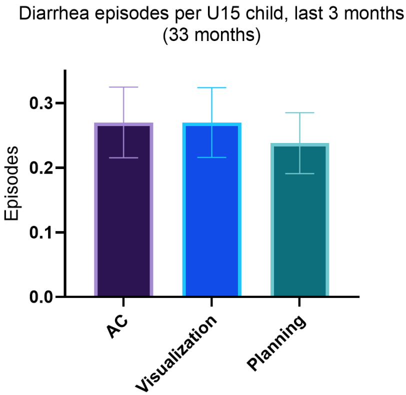

After 10 weeks, diarrhea reduced by 46 percent in V and 23 percent in P. Same for U5 children. No effect in LR, but confounded by seasonality (pos. effect for subsample surveyed in rainy season).

At cost US $4 per HH, classified "highly cost-effective" by WHO standards.

Savings

	Endline (10-12 weeks)				Follow-Up (30-36 months)			
	(1) Active Control Group Mean (SD)	(2) Visualization Treatment Effect	(3) Planning Treatment Effect	(4) Col. 2 vs. Col. 3 *p*-value	(6) Active Control Group Mean (SD)	(7) Visualization Treatment Effect	(8) Planning Treatment Effect	(9) Col. 2 vs. Col. 3 *p*-value
SAVINGS OUTCOMES								
Amount saved regularly (per week, KES)	93.96 (230.26)	24.37 (12.38)** [0.18]	3.58 (12.55) [1.00]	0.12	407.50 (605.28)	56.90 (33.78)* [0.12]	23.66 (33.29) [0.58]	0.34
Indicator: Amount saved regularly is positive	0.36 (0.48)	0.13 (0.03)*** [0.00]***	−0.02 (0.03) [1.00]	0.00***	0.78 (0.42)	0.05 (0.02)** [0.20]	0.02 (0.02) [1.00]	0.13
Number of ROSCAs [joined in last 3 months/total]	0.17 (0.44)	0.04 (0.03) [0.08]*	0.01 (0.02) [1.00]	0.24	1.08 (1.05)	0.12 (0.06)* [0.29]	0.10 (0.06) [1.00]	0.75
Weekly ROSCA savings	205.93 (304.72)	32.44 (15.73)** [0.04]**	11.22 (16.35) [1.00]	0.17	246.07 (363.71)	41.36 (22.13)* [0.12]	33.23 (21.21) [0.47]	0.72
Indicator: Saves for productive investments	0.17 (0.38)	0.11 (0.02)*** [0.00]***	−0.01 (0.02) [1.00]	0.00***	0.62 (0.49)	0.02 (0.02) [1.00]	0.01 (0.03) [1.00]	0.84
Total Savings Balance (KES)					2542.85 (6378.76)	1066.69 (428.62)** [0.20]	39.07 (404.17) [1.00]	0.01***

Both in short and long run, Visualization (but not Planning) led to significant increases in: Savings per week (↑ 26 percent), likelihood of saving regularly, savings in ROSCAs & number of ROSCAs joined, likelihood to save for productive investment, total savings balance (↑41 percent after 33 months despite Covid)

Labor Supply

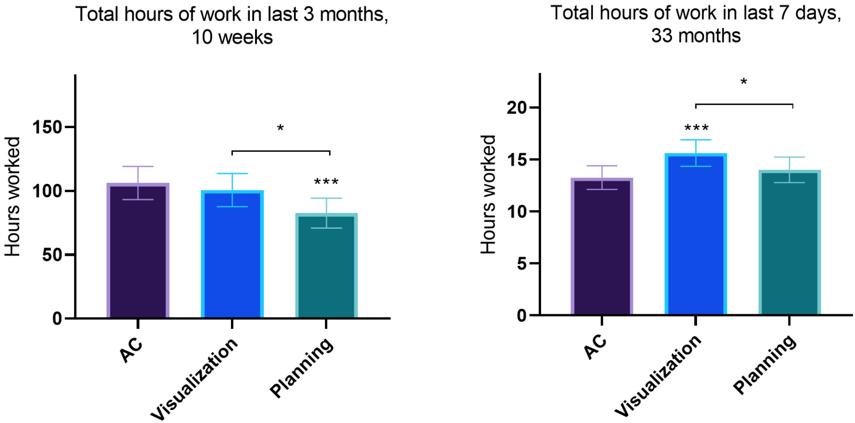

Increase of 18 percent in labor supply in Visualization in the long run. ▸ Labor Table
Labor supply reduced 22 percent in Planning in short run, no effect in long run.
No effects on earnings (all coefficients positive and insignificant).

Pre-Specified Psychological Mechanisms

Planning skills

- Measurement: Behavioral Activation scale (BADS), Tower of London task
- Result: No effects.

Measurement

Time Preferences

- Measurement: First incentivized real-effort task without access to computers or smartphones (SMS data entry in 0,1,7,8 days), plus monetary MPL
  - LR only: Utility forecasting task (à la Gabaix-Laibson)

Measurement

- Result: No effects on conventional $\beta\delta$–measures. 0.12 SD increase in forecasting vividness in V in long run.

Self-Efficacy

- Measurement: Generalized Self-Efficacy (GSE) scale.
- Result: SR increases in both V and P, LR increase only in V.

Psych Table

Self-Efficacy: Results

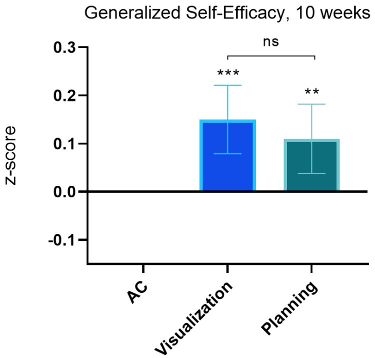

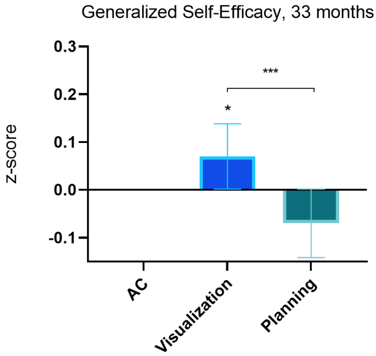

After 10 weeks, self-efficacy increases by 0.15 SD in V and 0.11 SD in P.
After 33 months, effect of V is 0.14 SD larger than P, 0.07 SD larger than AC.

Self-efficacy (but not $\beta\delta$ or planning skills) robustly correlated with chlorination and savings.

Self-Efficacy: Measurement

- General Self-Efficacy Scale (GSE) following Schwarzer/Jerusalem (1995)
- Validated and widely recognized in psych literature
  - Cronberg's alpha (internal reliability)$= 0.8$, Test-Retest $\rho = 0.62$
  - Selected based on a separate validation study

1	*I can always manage to solve difficult problems if I try hard enough.*
2	*If someone opposes me, I can find the means and ways to get what I want.*
3	*It is easy for me to stick to my aims and accomplish my goals.*
4	*I am confident that I could deal efficiently with unexpected events.*
5	*Thanks to my resourcefulness, I know how to handle unforeseen situations.*
6	*I can solve most problems if I invest the necessary effort.*
7	*When I am confronted with a problem, I can usually find several solutions.*
8	*If I am in trouble, I can usually think of a solution.*
9	*I can usually handle whatever comes my way.*

Utility forecasting: Vividness

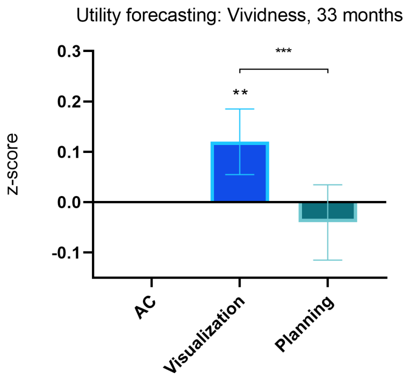

Increase of 0.12 SD after 33 months (no short-run measure). ▸ secondary measure
Vivid forecasting strongly predicts savings, but weaker for chlorination.

Utility forecasting: Measurement

- Need to find a proxy for the simulation noise $\sigma^2_{\epsilon_t}$ in Gabaix-Laibson model
  - Participants asked (and given time) to form an image in their mind of themselves and their family in the future.
  - Then asked to rate clarity and vividness of the image, from 1 (no image at all) to 7 (image as clear and vivid as real life).
  - Measure adapted from Plymouth Sensory Imagery Questionnaire (Andrade et al. 2014).

Utility forecasting: Measurement

- Need to find a proxy for the simulation noise $\sigma^2_{\epsilon_t}$ in Gabaix-Laibson model
  - Participants asked (and given time) to form an image in their mind of themselves and their family in the future.
  - Then asked to rate clarity and vividness of the image, from 1 (no image at all) to 7 (image as clear and vivid as real life).
  - Measure adapted from Plymouth Sensory Imagery Questionnaire (Andrade et al. 2014).

- *Why does utility forecasting respond, when conventional time preference measures don't?*
  - *Time horizon:* Visualization targets future in 1 year, while $\beta\delta$ measured from 0/1/7/8 days
  - *Abstract domain:* healthy kids & future visions vs. tangible SMS effort

$\rightarrow$ If model is correct, time preference measures will be sensitive to choice frame, setting, and domain.

Alternative Mechanisms

- Beliefs and knowledge about chlorination Table: Alternative Mechanisms
  - Information module included in V, P, AC. Does change beliefs relative to pure control, but no difference across active arms.
  - Information alone is ineffective: No effect on active relative to pure control.
- Risk preferences
  - No effects on incentivized Eckel-Grossman task
- Experimenter demand Exp. Demand
  - Chlorine tests unannounced, see paper for various checks.
  - Randomized demand treatments (de Quidt et al 2018) show no effect on self-reported chlorination.
- Salience and attention Salience
  - After 10 weeks, participants in V and P found it easier to remember chlorine-related words, conditional on total words remembered. Effect gets larger in V after 33 months. No effect on salience of savings.
  - Overall more consistent with reverse causality.

Conclusion

- Behavioural barriers may explain low demand for preventive health products, as well as other investment goods
- Light-touch psych interventions, esp. Visualization, can increase water chlorination, savings, and labor supply in short & long run.
  - Child diarrhea reduced in short run. Long run unclear given seasonality.
- **Policy:** Cost $4 per household, highly effective by WHO standards. Could integrate into curricula of community health workers, savings or microcredit organizations, or business training (Ashraf et al, *ongoing*).

Conclusion

- Behavioural barriers may explain low demand for preventive health products, as well as other investment goods
- Light-touch psych interventions, esp. Visualization, can increase water chlorination, savings, and labor supply in short & long run.
  - Child diarrhea reduced in short run. Long run unclear given seasonality.
- **Policy:** Cost $4 per household, highly effective by WHO standards. Could integrate into curricula of community health workers, savings or microcredit organizations, or business training (Ashraf et al, *ongoing*).
- **Takeaways and areas for future research:**
  1. Beliefs about self, as well as ability to visualize the future, appear to be (i) malleable to low-cost interventions, and (ii) powerful drivers of behavior
  2. The deeper underlying preferences and cognitive functions we consider appear less malleable. Caveat: need to better understand connections between conceptualizations of time preferences ($\beta\delta$ inherently unstable?).
  3. Need more work on mechanisms behind visualization.

Kremer et al (2011): Chlorine dispensers in Kenya

Figure 4: Chlorine Take-up Rates (in Sample 2)

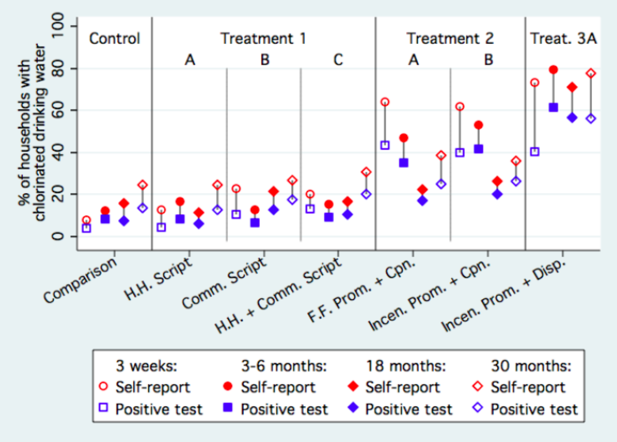

Notes: Bands depicted in graph above are not confidence intervals, but rather reflect an upper and lower bound measure of take-up (self-reported chlorination and positive chlorine tests, respectively). A positive chlorine test result is defined conservatively as sodium hypochlorite of at least 0.1 mg/L with pink color or 0.2 mg/L or greater regardless of color. See section 2.3 for a full description of the treatment arms: 0=Comparison (no intervention), 1A=Household persuasion script, 1B=Community persuasion script, 1C=Both household and community persuasion scripts, 2A=Flat-fee promoter plus one coupon for free WaterGuard per surveyed household, 2B=Incentivized promoter plus one coupon for free WaterGuard per surveyed household, 3=Incentivized promoter plus unlimited supply of free chlorine via a point-of-collection dispenser.

The Setting

- Eligibility: Women aged 18-35, $N = 3750$ in 205 villages

Baseline Data

- Mean age = 26
- Mean #children = 2.7 [93% have children, 73% of 18-19yo's]
- Occupations:
  - 60% subsistence farming, 19% no work, self-employed 16%, employed 3%
- Education: 64% Primary, 28% Secondary. Daily wage 150-400 Ksh.
- Water Source
  - 32% stream, 23% public tap, 20% private well, 16% public well
- Water treatment
  - 66% report having used chlorine at some point last month
  - but only 17% always chlorinate (5% always boil)
- 2014 Census: 15% of under-5's in Kenya had diarrhea in last two weeks, 26% are stunted

Specification

$$y_{i1} = \alpha + \beta_1 T_{1i} + \beta_2 T_{2i} + \delta y_{i0} + \Phi \mathrm{X}_i + \gamma_v + \theta_w + \varepsilon_i \quad (1)$$

- β_j = treatment effects
- $\mathrm{y_{i0}}$ = baseline outcome measure
- $\Phi \mathrm{X_i}$ = time invariant individual controls: age, education, marital status, employment status
- γ_v= village of residence fixed effect
- θ_w= indicator for above median wealth
- Standard errors clustered by intervention group
- Specification above is for comparison to active control group, and restricted to compliers ($N = 2175$)
- FDR q-value correction across groups of main outcomes (Anderson, 2008)

Balance and Attrition

- Balanced on demographic characteristics at baseline
- Conditional on attending baseline, attrition of 8, 12, and 10 percent in 10-week endline, 12-week chlorine test, and 33-months follow-up.
  - Balanced across active treatment arms
  - Neg. predicted by age, but not in interaction with treatment
- Non-participation in pure control group (24 percent) harder to interpret, as most chose not to participate in first place $\neq$ attrition
- Compliance: of recruited sample, 78 percent attended baseline & first intervention, 74 percent attended both sessions

Balance and Attrition

	Comparison with active control (AC+INF)					Comparison with pure control (PC)				
	(1) Active Control Group Mean (SD)	(2) Visualization Treatment Effect	(3) Planning Treatment Effect	(4) Col. 2 vs. Col. 3 *p*-value	(5) *N*	(6) Pure Control Mean (SD)	(7) V+INF Treatment Effect	(8) P+INF Treatment Effect	(9) AC+INF Treatment Effect	(10) *N*
Balance on recruitment census variables										
Age	26.57	−0.02	0.00	0.93	2337	26.62	−0.42	−0.36	−0.31	3750
	(4.52)	(0.22)	(0.22)			(4.69)	(0.22)*	(0.22)	(0.22)	
Married or cohabiting	0.89	−0.00	0.00	0.76	2337	0.90	−0.02	−0.01	−0.02	3750
	(0.31)	(0.02)	(0.02)			(0.30)	(0.02)	(0.01)	(0.02)	
Education level	5.85	0.01	0.08	0.19	2337	5.93	−0.08	0.00	−0.05	3750
	(1.22)	(0.06)	(0.06)			(1.08)	(0.05)	(0.05)	(0.05)	
High wealth index	0.55	−0.02	−0.03	0.67	2337	0.52	0.00	−0.01	0.02	3750
	(0.50)	(0.02)	(0.02)			(0.50)	(0.02)	(0.02)	(0.02)	
Village of residence	87.59	0.46	0.42	0.99	2337	83.31	0.80	−0.24	−0.07	3750
	(54.51)	(6.11)	(6.05)			(56.43)	(4.18)	(4.14)	(3.97)	
Survey participation										
Did not participate in endline (10w)	0.08	0.02	0.03	0.39	2337	0.24	−0.06	−0.04	−0.06	3750
	(0.27)	(0.02)	(0.02)*			(0.43)	(0.02)***	(0.02)*	(0.02)***	
Did not participate in chlorine test (12w)	0.12	0.01	0.03	0.33	2337	0.26	−0.04	−0.02	−0.04	3750
	(0.33)	(0.02)	(0.02)			(0.44)	(0.02)*	(0.02)	(0.02)**	
Did not participate in follow-up (33m)	0.10	0.02	0.02	0.85	2337	0.17	−0.01	−0.02	−0.01	3750
	(0.31)	(0.02)	(0.02)			(0.37)	(0.02)	(0.02)	(0.02)	
Delay variables										
Days between endline and baseline	67.73	0.62	1.80	0.23	2116	68.73	1.82	2.12	1.23	2984
	(20.63)	(1.00)	(0.93)*			(24.07)	(1.06)*	(1.01)**	(1.00)	
Days between chlorine test and baseline	79.19	0.73	2.62	0.16	2007	81.58	0.75	1.57	−0.01	2832
	(26.42)	(1.35)	(1.31)**			(27.27)	(1.21)	(1.17)	(1.16)	
Chlorine test was conducted on first day in village	0.68	0.01	−0.00	0.69	2009	0.67	−0.01	−0.01	−0.01	2839
	(0.47)	(0.02)	(0.02)			(0.47)	(0.02)	(0.02)	(0.02)	
Days between follow-up and baseline	980.47	2.76	2.02	0.70	2066	987.06	−1.16	−2.35	−4.36	2828
	(33.29)	(1.98)	(1.86)			(36.04)	(1.91)	(1.82)	(1.84)**	
Compliance										
Completed baseline & both interventions	0.74	0.01	−0.02	0.35	2975	–	–	–	–	-
	(0.44)	(0.02)	(0.02)			–	–	–	–	-
Completed baseline & first intervention	0.78	0.01	0.01	0.85	2975	–	–	–	–	-
	(0.41)	(0.02)	(0.02)			–	–	–	–	-

Treatment Effects: Health

	Endline (10-12 weeks)				Follow-Up (30-36 months)			
	(1) Active Control Group Mean (SD)	(2) Visualization Treatment Effect	(3) Planning Treatment Effect	(4) Col. 2 vs. Col. 3 *p*-value	(6) Active Control Group Mean (SD)	(7) Visualization Treatment Effect	(8) Planning Treatment Effect	(9) Col. 2 vs. Col. 3 *p*-value
HEALTH OUTCOMES								
Objective measure: chlorine present in water (TCR)	0.23	0.05	0.02	0.15				
	(0.42)	(0.02)**	(0.02)					
Objective measure: chlorine sufficient to be safe (FCR)	0.21	0.04	0.01	0.16				
	(0.40)	(0.02)**	(0.02)					
		[0.04]**	[1.00]					
Main Treatment: Chlorine (self-report)	0.73	0.07	−0.00	0.00***	0.85	0.05	0.02	0.15
	(0.45)	(0.02)***	(0.02)		(0.35)	(0.02)***	(0.02)	
		[0.00]***	[1.00]			[0.03]**	[0.39]	
Main Treatment: Boil (self-report)	0.35	0.07	0.05	0.46	0.63	0.03	−0.01	0.19
	(0.48)	(0.03)***	(0.03)*		(0.48)	(0.03)	(0.03)	
		[0.01]**	[0.39]			[1.00]	[1.00]	
Diarrhea incidences per child u15, last 3 months	0.26	−0.12	−0.06	0.09*	0.27	0.00	−0.04	0.26
	(0.69)	(0.03)***	(0.03)*		(0.73)	(0.04)	(0.03)	
		[0.00]***	[0.39]			[0.33]	[0.39]	
Diarrhea incidences per child u5, last 3 months	0.34	−0.16	−0.06	0.02**	0.39	0.01	−0.01	0.70
	(0.86)	(0.05)***	(0.05)		(1.04)	(0.07)	(0.06)	
		[0.00]***	[1.00]			[1.00]	[1.00]	
Proportion of children taken for healthcare check-up	0.21	−0.04	−0.02	0.34	0.36	−0.02	−0.01	0.88
	(0.34)	(0.02)**	(0.02)		(0.37)	(0.02)	(0.02)	
		[0.03]**	[1.00]			[1.00]	[1.00]	
Proportion of children u15 vaccinated, last 3 months	0.22	0.00	−0.01	0.49				
	(0.35)	(0.02)	(0.02)					
		[0.38]	[1.00]					
Number of ANC visits, last 3 months (if pregnant)	1.26	−0.10	0.21	0.45				
	(1.19)	(0.49)	(0.36)					
		[0.38]	[1.00]					

Heterogeneous Treatment Effects by Dispenser Status

	Village has no chlorine dispenser				Village has chlorine dispenser				Comparison	
	(1) Active Control Mean (SD)	(2) V+INF Treatment Effect	(3) P+INF Treatment Effect	(4) *N*	(5) Active Control Mean (SD)	(6) V+INF Treatment Effect	(7) P+INF Treatment Effect	(8) *N*	(9) V+INF Interaction *p*-value	(10) P+INF Interaction *p*-value
ENDLINE: 10 - 12 WEEKS										
Objective measure: chlorine present in water (TCR)	0.19	0.05	0.04	1082	0.29	0.04	−0.00	930	[0.87]	[0.34]
	(0.39)	(0.03)*	(0.03)		(0.45)	(0.03)	(0.03)			
Objective measure: chlorine sufficient to be safe (FCR)	0.16	0.05	0.03	1082	0.27	0.03	−0.01	930	[0.60]	[0.32]
	(0.36)	(0.03)**	(0.03)		(0.44)	(0.03)	(0.03)			
Main Treatment: Chlorine (self-report)	0.65	0.09	0.02	1129	0.82	0.06	−0.01	987	[0.34]	[0.49]
	(0.48)	(0.03)***	(0.03)		(0.39)	(0.03)*	(0.03)			
Main Treatment: Boiling (self-report)	0.37	0.06	0.04	1129	0.33	0.09	0.07	987	[0.52]	[0.56]
	(0.48)	(0.04)	(0.04)		(0.47)	(0.04)**	(0.04)*			
Diarrhea incidences per child u15, last 3 months	0.26	−0.10	−0.04	1066	0.25	−0.13	−0.09	938	[0.69]	[0.37]
	(0.67)	(0.05)**	(0.05)		(0.71)	(0.05)**	(0.05)*			
Diarrhea incidences per child u5, last 3 months	0.37	−0.15	−0.05	908	0.30	−0.15	−0.06	774	[0.97]	[0.93]
	(0.88)	(0.06)**	(0.07)		(0.83)	(0.06)**	(0.07)			
FOLLOW-UP: 30 - 36 MONTHS										
Main Treatment: Chlorine (self-report)	0.81	0.05	0.02	1103	0.90	0.04	0.03	970	[0.78]	[0.78]
	(0.39)	(0.03)*	(0.03)		(0.30)	(0.02)**	(0.02)			
Main Treatment: Boiling (self-report)	0.63	0.04	0.02	1103	0.62	0.02	−0.03	970	[0.67]	[0.37]
	(0.48)	(0.04)	(0.04)		(0.49)	(0.04)	(0.04)			
Diarrhea incidences per child u15, last 3 months	0.29	0.01	−0.10	1088	0.25	−0.04	−0.02	957	[0.48]	[0.23]
	(0.71)	(0.06)	(0.05)**		(0.76)	(0.06)	(0.05)			
Diarrhea incidences per child u5, last 3 months	0.46	−0.02	−0.09	867	0.32	0.02	0.06	745	[0.74]	[0.15]
	(1.12)	(0.09)	(0.09)		(0.93)	(0.09)	(0.08)			
Chlorine source: Bottle	0.47	0.06	0.04	1103	0.31	0.02	0.06	970	[0.48]	[0.77]
	(0.50)	(0.04)	(0.04)		(0.46)	(0.04)	(0.04)			
Chlorine source: Dispenser	0.21	−0.04	−0.04	1103	0.54	−0.01	−0.05	970	[0.57]	[0.93]
	(0.41)	(0.03)	(0.03)		(0.50)	(0.04)	(0.04)			
Reports working dispenser at main source	0.34	−0.03	0.01	1103	0.70	0.03	−0.04	970	[0.17]	[0.33]
	(0.47)	(0.04)	(0.04)		(0.46)	(0.03)	(0.03)			
Reports working dispenser within 30min walk	0.49	−0.01	−0.02	1103	0.82	0.01	−0.01	970	[0.57]	[0.77]
	(0.50)	(0.04)	(0.04)		(0.38)	(0.03)	(0.03)			

Treatment Effects: Labor Supply

	Endline (10-12 weeks)				Follow-Up (30-36 months)			
	(1) Active Control Group Mean (SD)	(2) Visualization Treatment Effect	(3) Planning Treatment Effect	(4) Col. 2 vs. Col. 3 *p*-value	(6) Active Control Group Mean (SD)	(7) Visualization Treatment Effect	(8) Planning Treatment Effect	(9) Col. 2 vs. Col. 3 *p*-value
LABOR OUTCOMES								
Total hours of work [last 3 months/ last 7 days]	106.11 (174.61)	−5.38 (9.54) [0.85]	−23.39 (8.93)*** [0.03]**	0.05*	13.25 (15.41)	2.36 (0.88)*** [0.06]*	0.89 (0.87) [0.47]	0.09*
Total days of work, last 3 months	21.22 (30.09)	−0.31 (1.62) [0.38]	−3.70 (1.58** [0.37]	0.04**				
Earnings, cash and in-kind [monthly/last 7 days]	1094.50 (2865.35)	0.17 (147.14) [0.40]	10.16 (163.57) [1.00]	0.94	677.26 (1343.83)	104.62 (77.27) [0.94]	101.91 (80.34) [1.00]	0.97
OTHER BEHAVIOURAL OUTCOMES								
Index of investment in children's education (z-score)	0.00 (1.00)	−0.02 (0.06) [0.85]	0.00 (0.07) [1.00]	0.68				

Pre-Specified Psychological Mechanisms

	Endline (10-12 weeks)				Follow-Up (30-36 months)			
	(1) Active Control Group Mean (SD)	(2) Visualization Treatment Effect	(3) Planning Treatment Effect	(4) Column 2 vs. Column 3 *p*-value	(6) Active Control Group Mean (SD)	(7) Visualization Treatment Effect	(8) Planning Treatment Effect	(9) Column 2 vs. Column 3 *p*-value
PLANNING SKILLS								
BADS score (z-score)	0.00<br>(1.00)	−0.03<br>(0.05)<br>[0.83]	0.05<br>(0.05)<br>[0.91]	0.21				
Tower of London (z-score)	0.00<br>(1.00)	0.02<br>(0.05)<br>[0.49]	−0.04<br>(0.05)<br>[0.39]	0.25				
TIME PREFERENCES								
β^{Effort}	0.982<br>(0.005)	0.007<br>(0.006)<br>[0.83]	0.005<br>(0.007)<br>[0.91]	0.33				
δ^{Effort}	0.999<br>(0.001)	−0.001<br>(0.001)<br>[0.26]	−0.002<br>(0.001)**<br>[0.08]*	0.16				
Utility Forecasting: Vividness (z-score)					0.00<br>(1.00)	0.12<br>(0.05)**<br>[0.04]**	−0.04<br>(0.06)<br>[0.72]	0.00***
Utility Forecasting: Practice (z-score)					−0.00<br>(1.00)	0.10<br>(0.05)*<br>[0.17]	0.12<br>(0.05)**<br>[0.09]*	0.68
SELF-EFFICACY								
General Self-Efficacy Score (GSE) (z-score)	0.00<br>(1.00)	0.15<br>(0.05)***<br>[0.01]***	0.11<br>(0.05)**<br>[0.08]*	0.45	−0.00<br>(1.00)	0.07<br>(0.05)<br>[0.09]*	−0.07<br>(0.05)<br>[0.72]	0.01***

Alternative Mechanisms

	Endline (10-12 weeks)				Follow-Up (30-36 months)			
	(1) Active Control Group Mean (SD)	(2) Visualization Treatment Effect	(3) Planning Treatment Effect	(4) Column 2 vs. Column 3 *p*-value	(6) Active Control Group Mean (SD)	(7) Visualization Treatment Effect	(8) Planning Treatment Effect	(9) Column 2 vs. Column 3 *p*-value
BELIEFS AND KNOWLEDGE								
Belief: Diarrhea avoided through chlorination (z-score)	0.00	0.08	0.05	0.56				
	(1.00)	(0.05)	(0.05)					
Chlorine knowledge score (z-score)	0.00	0.07	0.00	0.22				
	(1.00)	(0.05)	(0.05)					
ANC/PNC knowledge score (z-score)	0.00	0.04	−0.05	0.09*				
	(1.00)	(0.05)	(0.05)					
Risk Aversion Measure (z-score)	0.00	−0.04	−0.08	0.52				
	(1.00)	(0.06)	(0.06)					
SALIENCE TASK								
Chlorine word remembered	0.40	0.06	0.03	0.095*	0.42	0.10	0.04	0.028**
	(0.49)	(0.01)***	(0.01)**		(0.49)	(0.03)**	(0.03)	
Savings word remembered	0.45	−0.02	0.00	0.124	0.45	0.04	-0.01	0.069*
	(0.50)	(0.01)	(0.01)		(0.50)	(0.02)	(0.02)	
Total words remembered	4.23	−0.09	0.04	0.105	4.45	0.28	0.20	0.336
	(1.64)	(0.07)	(0.07)		(1.61)	(0.09)**	(0.09)*	

Planning Skills: Measurement

- Planning skills measured with the incentivized *Tower of London task* (Shallice, 1982; Phillips et al. 2001)
  - measures a participant's ability to plan ahead in sequential strategies
  - Selected based on a separate validation study (*SOBC1*)

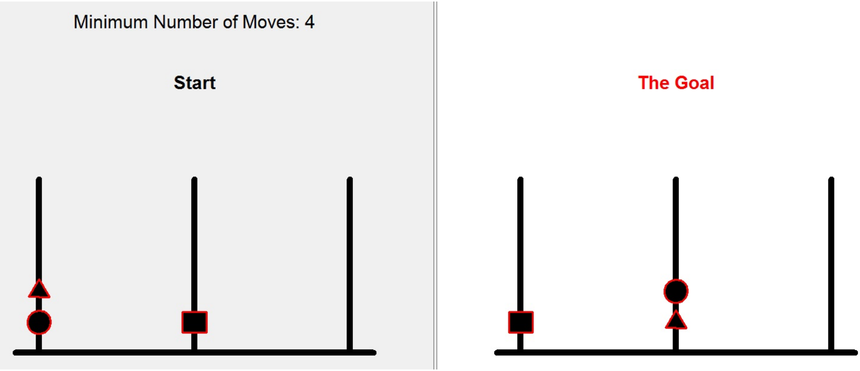

Time Preferences over Effort: Measurement

Based on Augenblick (2017):

- "*How many tasks do you want to do at time X, at piece rate Y?*"
  - where $X = \{\text{same evening, tomorrow, 7 days, 8 days}\}$ and $Y = \{2,\ 6,\ 10\}$ Kenyan shillings
- One question randomly selected to "count"

Effort task in Kenya: SMS Data entry task (30 digits ≈2min)

Task 1	SMS1- 969 228 853 496 963 294 743 281 619 446
Task 2	SMS2- 151 575 320 519 150 525 175 694 371 897

Structural estimation:

- Variation in Y allows estimation of convex cost of effort γ, variation in X estimates $\beta\delta-$discount function

Time Preferences over Effort: Structural Estimation

- Follows Augenblick (2017) and DellaVigna and Pope (2017):
  - Power cost of effort function γ, quasi-linear utility
  - Quasi-hyperbolic discounting ($\beta\delta$)
  - Non-monetary reward s ($\approx$ intrinsic motivation)
  - We add weekday dummies d_w for different opportunity cost of time
- The optimal level of effort is then given by

$$e* = argmax\ (s + D_m(14) \cdot \phi \cdot w) \cdot e - \beta^{I(t>0)} \cdot \delta^t \cdot (\frac{1}{\gamma} e^\gamma + d_w \cdot e)$$

where $w = \{2, 6, 10\}$ is the piece rate,
$D_m(14)$ is monetary discounting of the payment in 14 days
t is the time of effort provision
$\gamma > 1$ captures convex costs of effort,
ϕ is a slope parameter, and d_w are weekday indicators

- We estimate additive treatment effects of V, P, and AC on the parameters β, δ, s, and γ.

Time Preferences: Raw Effort Responses

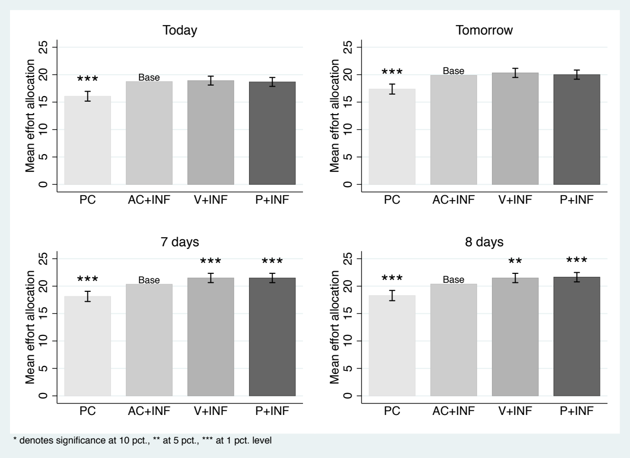

- Same effort across AC - P - V for 0 and 1 days
- Significantly higher effort in P and V at 7 and 8 days
- Pure control always supplies less effort (they haven't done the task before)

Utility forecasting: Practice

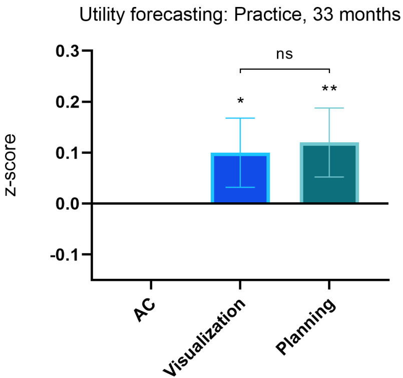

Agreement with "When I have to make an decision, I try to paint a clear picture of the consequences of that decision." Likely confounded by self-efficacy.

Possible interpretation: Effects on behavior are driven by forecasting *ability*, rather than just regular practice of imagining consequences (thus no effects in P).

Gabaix and Laibson (2017): Simulating Future Utility

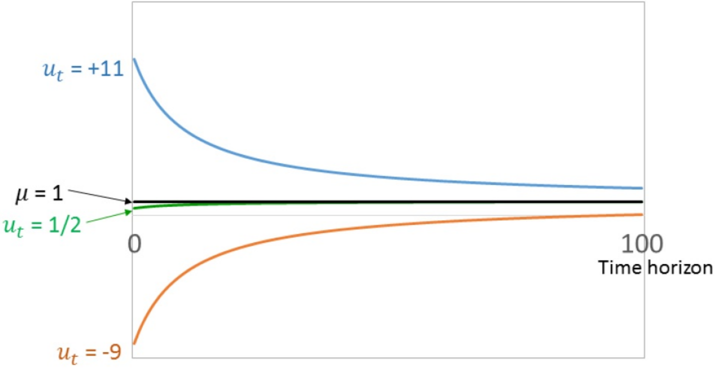

Figure 1: Plot of the average perceived value $\overline{u}_t$, given for three different true utilities u_t ($u_t \in \{-9, 1/2, 11\}$), as a function of the time horizon t. This average perceived value is: $\overline{u}_t = \mu + \frac{u_t - \mu}{1 + \frac{\sigma_\varepsilon^2}{\sigma_u^2} t}$. The figure uses $\sigma_\varepsilon^2 / \sigma_u^2 = 0.1$.

Experimenter Demand

LR survey included randomized experimenter demand treatments (de Quidt et al 2018). Respondents randomly assigned to a group A (B).

Told "*We hypothesize that people who participated in this study and received the same treatment as you will give higher (lower) responses to these questions than others.*" Then asked how often they added chlorine to water in the last 7 days.

	$\alpha^{+}(\zeta)$ Mean(SD)	$\alpha^{-}(\zeta)$ Mean (SD)	Test: $\alpha^{-}(\zeta) = \alpha^{+}(\zeta)$ p-value
Visualization	2.337	2.264	0.539
	(1.773)	(1.695)	
Planning	2.269	2.149	0.394
	(2.375)	(1.649)	
Active Control	2.087	2.291	0.089*
	(1.742)	(1.734)	
Pure Control	2.163	2.174	0.929
	(1.644)	(1.595)	
All Treatment Groups Combined	2.217	2.223	0.923
	(1.924)	(1.674)	
Obs	1556	1616	3,172

Responses used to obtain bounds $a^{+}(\zeta)$ and $a^{-}(\zeta)$ for the impact of experimenter demand effects on self-reports.

Salience

- Our psych interventions may have differentially increased the salience of water chlorination
  - V and P scripts domain general, but chlorine used as example
- We measure salience of three future-oriented behaviors (chlorination, savings, and farm investment)
- Salience test:
  - SR (LR): Enumerators read out 3 (1) lists of 9 words. Each list contained one word relating to chlorine, savings, farm investment, plus six filler words
  - Conceptually similar to audio word search task in Lichand and Mani (2016).
- Test for differential recall of future-oriented words, controlling for #total words remembered

Salience

- SR: Participants in V (P) were 6pp*** (3pp**) more likely to remember the chlorine word in a given word list. Table
- LR: Effect in V increases to 10pp**, disappears in P.
- Salience of savings unaffected in SR or LR.

Three possibilities:

1. Salience as a driver of treatment effects
2. Reverse causality: More chlorination → more salience
3. Changes in salience unrelated to changes in behavior

Evidence:

- References to chlorination in V and P were identical, but effects persist only in V → 2.
- Salience effects appear to increase over time → likely 2.
- No correlation between salience of chlorination and chlorination → 3.
- Savings changes but salience of savings does not → 3.
